# Novel Pyrone-Based Biofilm Inhibitors against Azole-Resistant *Candida albicans*

**DOI:** 10.1021/acsomega.5c04793

**Published:** 2025-08-11

**Authors:** Ji-eun Yang, Jin-Hyung Lee, Bharath Reddy Boya, Yong-Guy Kim, Youngjoo Byun, Jintae Lee

**Affiliations:** † College of Pharmacy, 65692 Korea University, 2511 Sejong-ro, Sejong 30019, Republic of Korea; ‡ School of Chemical Engineering, 35032 Yeungnam University, Gyeongsan, Kyeongbuk 38541, Republic of Korea; § Interdisciplinary Major Program in Innovative Pharmaceutical Sciences, 65692 Korea University, Sejong 30019, Republic of Korea

## Abstract

*Candida albicans* is an opportunistic fungus that is pathogenic in immunocompromised patients with life-threatening diseases such as HIV and cancer. *C. albicans* is the most common fungal species isolated from biofilms formed on implanted medical devices or on human tissue. Biofilm development of *C. albicans* is mainly driven by a transition from yeast to hyphal form involving core proteins such as HWP and ALS. We designed and synthesized novel α-pyrone-based analogues to investigate their potential in inhibiting biofilm formation and hyphal development of *C. albicans* . Among the synthesized compounds, three compounds (**6f**, **6j**, and **6n**) significantly inhibited *C. albicans* biofilm formation and reduced cell aggregation and hyphal formation in a dose-dependent manner. These compounds had minimal effects on planktonic cell growth while significantly reducing biofilm formation at 20–50 μg/mL, suggesting novel candidate compounds for managing drug-resistant strains of *C. albicans* . The three compounds may represent promising therapeutic options with potential synergistic effects when combined with existing antifungal agents.

## Graphical abstract

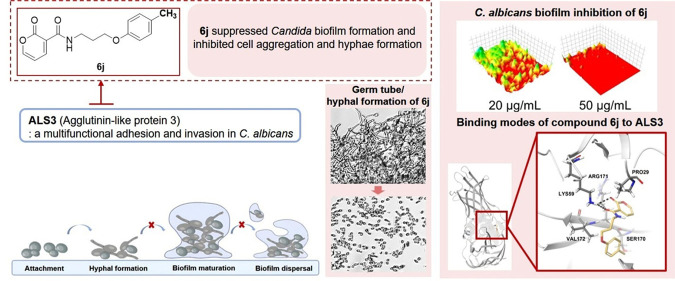

## 1 Introduction

*Candida albicans* is an opportunistic fungal organism that frequently inhabits the normal human microbiota, particularly in the oral, gastrointestinal, and vaginal regions.^1,2^ Although it generally remains nonpathogenic in immunocompetent hosts, it can cause serious infections, such as invasive candidiasis, in populations with compromised immune systems (e.g., patients with cancer, HIV/AIDS, or those receiving immunosuppressive therapy).^3,4^ These infections can present with a variety of clinical manifestations, including candidemia, oral candidiasis, and vaginal candidiasis, and can place a significant burden on healthcare infrastructure, especially in severely ill or long-term hospitalized patients, with a high mortality rate. Indeed, 1.7 million deaths worldwide are recorded each year due to fungal infections, with a significant number of these attributed to *C. albicans* and other related *Candida* species.^2,5^

A pivotal virulence determinant of *C. albicans* is its capacity to develop biofilms.^6^ Biofilms constitute intricate three-dimensional structures comprising a matrix of polysaccharides, proteins, and nucleic acids that confer protection to the encapsulated microorganisms from external stresses or antifungal interventions, in addition to promoting intercellular communication and nutrient acquisition.^7,8^ The progression of biofilm formation in *C. albicans* typically unfolds through four principal stages, characterized by a transition from the yeast phenotype to the hyphal phenotype: (1) adherence of yeast cells to a substrate to create a foundational layer, (2) proliferation and establishment of a securely attached layer, (3) maturation during which intense synthesis of extracellular matrix results in a resilient three-dimensional architecture, and (4) dispersal, wherein a fraction of yeast cells detaches from the biofilm to colonize new niches.^9–11^

Importantly, the hyphal form serves an important structural function within the biofilm, facilitating deeper penetration into medical devices (e.g., catheters and prosthetics) and host tissues, thereby impeding the accessibility of antifungal agents.^12,13^ As a result, biofilm-associated *C. albicans* exhibits significantly higher levels of drug resistance than their planktonic (free-living) counterparts, and frequently acquires resistance to standard antifungal agents (e.g., fluconazole), resulting in recurrent or chronic infections.^7,14,15^

In *C. albicans* , various proteins are associated with the process of hyphal formation and then biofilm development. Particularly, hyphal wall protein 1 and 2 (HWP1 and HWP2),^16,17^ agglutinin-like sequence 1 and 3 (ALS1 and ALS3) positively regulate hyphal and biofilm formation.^18–20^ These proteins are indispensable not only for the transition from yeast to hyphae phenotype but also for ensuring robust adherence and structural integrity within the biofilm.^21^ Hence, the identification of molecules or compounds that inhibit the expression or functionality of these two proteins provides a promising avenue for mitigating biofilm formation and antifungal resistance of *C. albicans* . Indeed, mechanisms that target such proteins possess the potential to function synergistically with existing antifungal agents in the context of combination therapy.^22^

α-Pyrone derivatives, defined by their six-membered lactone framework, have been documented in the scientific literature to exhibit a variety of biological activities, including but not limited to cytotoxicity, antibacterial, and antifungal effects.^23–26^ For instance, α-pyrone derivatives such as pseudopyronine A, B, and C, originating from *Pseudomonas* species, are known to inhibit biofilm formation in Gram-positive *Staphylococcus aureus* and significantly reduce mature biofilms.^27^ Additionally, pyrone analogs have been found to inhibit the binding of OdDHL (*N*-3-(oxododecanoyl)-L-homoserine lactone) to the LasR in *Pseudomonas aeruginosa* , thereby suppressing the quorum sensing (QS) process and concurrently inhibiting the formation of biofilms.^28^ Recent studies by Borkar and colleagues have shown that β-pyrone derivatives not only exhibit antimicrobial activity against *Mycobacterium smegmatis* but also demonstrate substantial inhibitory effects on biofilm formation, indicating their potential application as antimycobacterial agents.^29^ Furthermore, coumarin compounds with an α-pyrone skeleton have also been reported to effectively control biofilm formation and regulate QS in various pathogenic microorganisms, suggesting that the broader class of pyrone derivatives may be widely applicable as biofilm inhibitors.^30^ In addition to using the α-pyrone scaffold due to its reported biofilm-inhibitory potential,^28^ we selected substituents on the phenoxypropyl moiety to explore the effects of electron-donating and electron-withdrawing groups, as well as their positional isomerism (*ortho*, *meta*, and *para*). These modifications were intended to systematically investigate the influence of steric and electronic factors on antibiofilm activity and to establish a structure–activity relationship (SAR).

Based on these properties, in this study, we designed and synthesized novel inhibitors using the α-pyrone scaffold that specifically interact with an important hyphae-related protein ALS3 or pathways involved in biofilm formation and hyphal transition of *C. albicans* . To evaluate these compounds, we tested their antibiofilm activity at a concentration of 20–50 μg/mL against fluconazole-resistant *C. albicans* DAY185 using crystal violet staining, assessed the inhibition of hyphal formation and cell aggregation via microscopic observation and molecular docking of ALS3 protein, thereby possessing the potential as novel biofilm inhibitors of *C. albicans* .

## 2 Results and Discussion

### 2.1 Chemistry

By introducing various substituents on the α-pyrone core scaffold, we aimed to identify novel biofilm inhibitors and establish the structure–activity relationship (SAR) governing biofilm formation. Novel compounds for inhibiting *C. albicans* biofilm were designed and synthesized using pyrone derivatives as the starting materials. The overall synthetic strategy is summarized in Scheme 1. The process included: (1) Boc protection of alkylamines and subsequent coupling with phenolic derivatives, (2) Boc deprotection to obtain free amines, and (3) amide bond formation between the amine intermediates and acid chloride compounds with α-pyrone scaffold. A total of 20 derivatives were synthesized (Scheme 1). The chemical structure of the final compounds was analyzed by NMR, HPLC and HRMS.

**Scheme 1:**
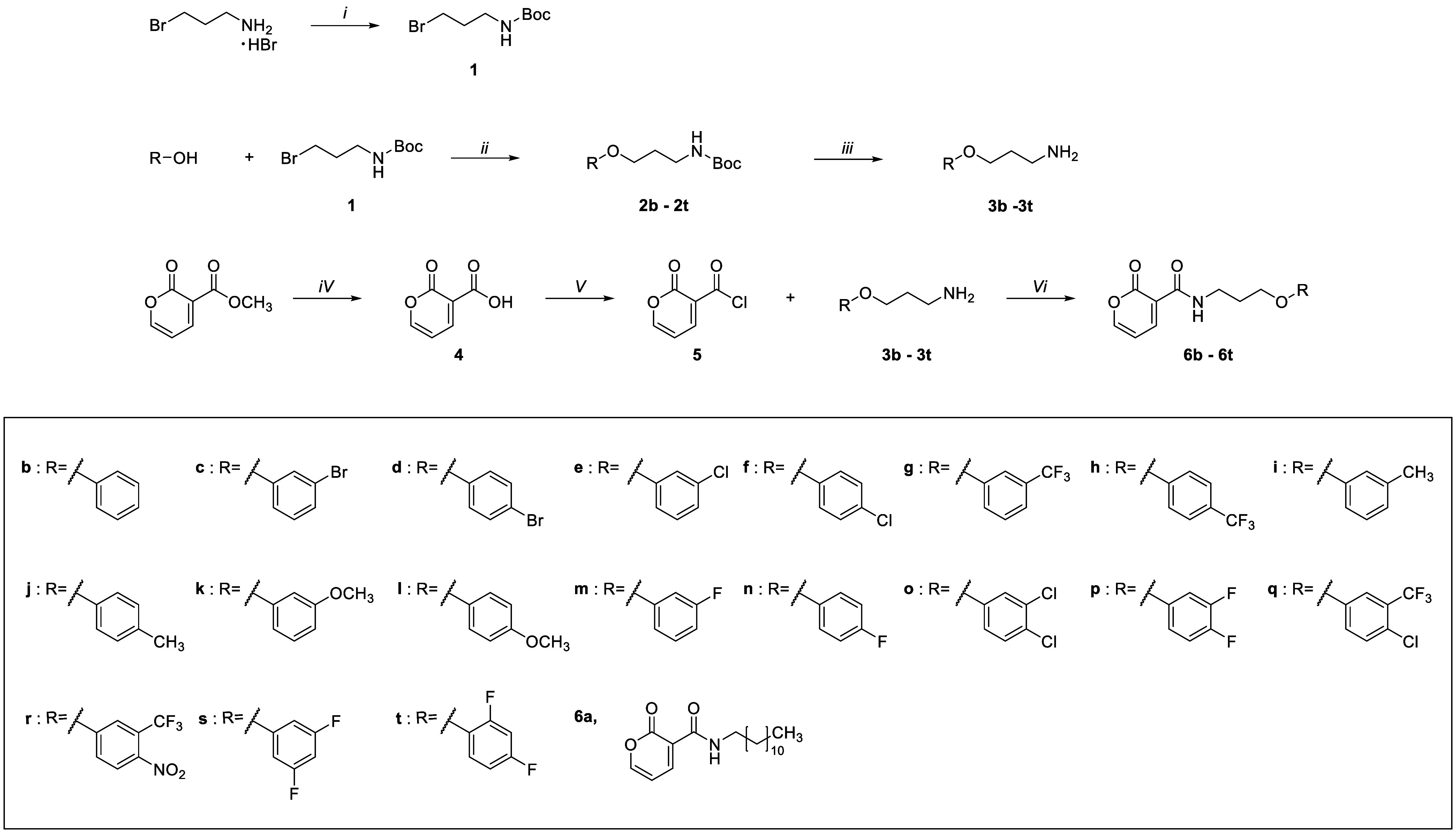
Synthesis of Pyrone Derivatives^a^

a Reagents and conditions: (*i*) Boc_2_O, MeOH, rt, 6 h, 61–88% yield; (*ii*) Cs_2_CO_3_, DMF, 90 °C, 24 h, 70–80% yield; (*iii*) TFA, CH_2_Cl_2_, rt, 3 h, 57–74% yield; (*iv*) *c-*HCl, 45 °C, 24 h, 50% yield; (*v*) (COCl)_2_, *cat*. DMF, CH_2_Cl_2_, r.t, 3 h; (*vi*) TEA, DCM, r.t, 24 h, 8–26% yield.

Briefly, commercial 3-bromopropylamine hydrobromide was reacted with di-*tert*-butyl dicarbonate (Boc_2_O) and triethylamine (TEA) in methanol to afford *tert*-butyl 3-bromopropylcarbamate (**1**). Subsequently, compound **1** was *O*-alkylated with various phenol derivatives having diverse substituents in the presence of potassium carbonate (K_2_CO_3_) in DMF to give ether intermediates (**2b–2t**) in yields ranging from 55 to 89%. For the compounds bearing fluoro (−F) group in the *meta* or *para* position (**2o–2t**), the initial yields were considerably low because the strong electron-withdrawing substituents hindered the formation of the phenolate anion or the steric hindrance reduced the accessibility of the nucleophile to the reaction site. To solve the low yield problem, K_2_CO_3_ was replaced by cesium carbonate (Cs_2_CO_3_), which can effectively stabilize the phenolate anion in DMF, to increase the reaction yield to the range of 60–80%. Subsequent deprotection of the Boc protecting group was achieved by treatment with trifluoroacetic acid (TFA) in dichloromethane, affording the free amine intermediates (**3b–3t**). These intermediates (**3b–3t**) were not isolated or purified and were directly used in the subsequent reaction.

To prepare the α-pyrone scaffold, methyl 2-oxo-2H-pyran-3-carboxylate was heated in the presence of concentrated HCl to hydrolyze the methoxy group to give compound **4** in 49% yield. This intermediate was then converted to the corresponding acid chloride (**5**) using oxalyl chloride and a catalytic amount of DMF. The amine precursors (**3a**–**3t**) were reacted with compound **5** to give the final amide compounds (**6a**–**6t**) in the yield range of 8–26%.

### 2.2 Antibiofilm Activities of Pyrone Derivatives

The final compounds (**6a–6t**) were evaluated for their antibiofilm activity against *C. albicans* . Initially, the antibiofilm activity of 20 pyrone derivatives was evaluated against fluconazole-resistant *C. albicans* DAY185 at a concentration of 20 and 50 μg/mL (Figure 1A). Notably, the 20 derivatives showed significant differences in antibiofilm potency. A total of 15 compounds at 50 μg/mL were found to significantly inhibit biofilm formation. It is noteworthy that compounds **6b–6l** significantly reduced *C. albicans* biofilm formation by more than 84%. These initial findings prompted further investigation at a concentration of 20 μg/mL, compared with antifungal agents butoconazole, ketoconazole, and amphotericin B in the previous report.^45^ It appears that **6b–6l** showed better antibiofilm activity than ketoconazole but less active than butoconazole, and amphotericin B. Compounds **6s** and **6t** also demonstrated meaningful antibiofilm activity at the tested concentrations. In the second screening round, derivatives **6f**, **6j**, and **6n** were selected for further study due to their prominent activities. Subsequent detailed biofilm assays revealed that these three compounds inhibited *Candida* biofilm formation in a dose-dependent manner (Figure 1B–D). Specifically, at a concentration of 20 μg/mL, each of the three compounds inhibited biofilm formation by more than 72%.

**Figure 1:**
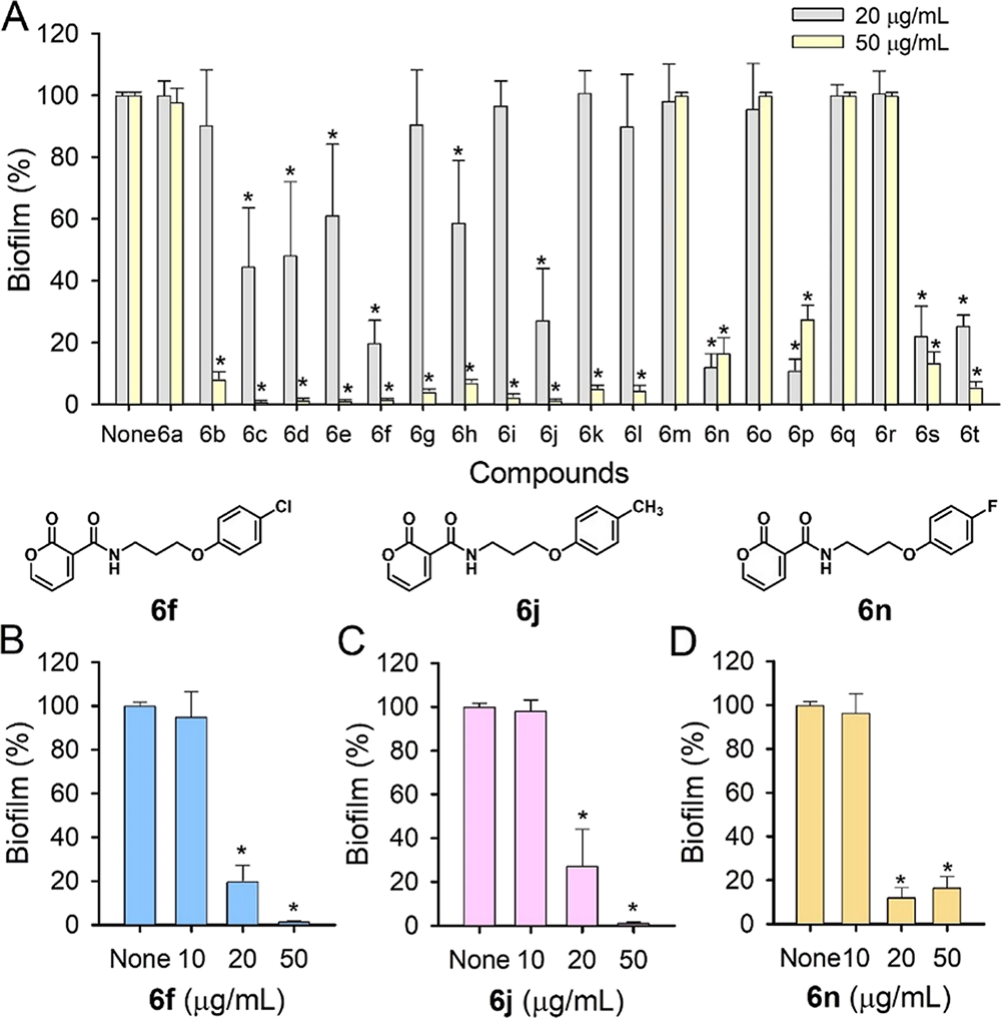
Antibiofilm screening of 20 pyrone derivatives at 20 and 50 μg/mL (A) and dose-dependent antibiofilm evaluation against *C. albicans* (B–D). Asterisks (*) indicate significant differences of biofilm formation (*p* < 0.05), and error bars display the standard deviation.

### 2.3 Structure–Activity Relationship (SAR) Analysis

The *C. albicans* biofilm inhibitory activity of the synthesized compounds (Scheme 1) was compared with the structural characteristics of the substituents, the following structure–activity relationships were observed. First, with respect to the substitution positions, the *para*-substituted compounds (**6f, 6h, 6j**) generally exhibited stronger inhibitory activities than their corresponding *meta*-substituted counterparts (**6e, 6g, 6k**). This suggests that para-substitution may enhance biofilm inhibition, potentially due to the difference in substitution position and electronic effects.

Interestingly, a simple classification of substituents into electron-donating (e.g., −OCH_3_, −CH_3_) and electron-withdrawing (e.g., −CF_3_, −F, −Cl, −Br) groups did not reveal a strong correlation with antibiofilm activity. In contrast, substituent size and halogen electronegativity appear to be more determining factors. Compounds with smaller substituents tend to exhibit relatively higher biofilm inhibition activity, regardless of the electronic effectiveness of the substituents. For example, compounds substituted with fluoro-, chloro-, or methyl groups (**6f, 6j, 6n**) exhibited stronger inhibition activity than compounds substituted with bromo-, trifluoromethyl-, or methoxy substituents (**6d, 6h, 6l**).

In particular, the fluoro-substituted compound (**6n**), characterized by high electronegativity and smaller atomic radius, showed excellent inhibitory activity among the halogen-substituted compounds. This result may be due to the minimization of steric hindrance and the more efficient involvement of the fluoro group in the internal electron distribution of the molecule. Overall, considering both substituent size and electronic properties, smaller substituents with higher electronegativity are advantageous for *C. albicans* biofilm inhibition in α-pyrone derivatives.

### 2.4 Microscopic Observation of Biofilm, Aggregation, and Hyphae Inhibition

Microscopic visualization confirmed that the three compounds significantly inhibited *Candida* biofilm formation (Figure 2A). In the nontreated control, a dense biofilm was observed, indicated by a blue color. Treatment with the three compounds at concentrations of 20 and 50 μg/mL reduced biofilm formation, as depicted by the transition to green, yellow, and red colors in the 3D representation. Additionally, the effects of the three active compounds on planktonic cell growth were investigated over a 24-h period. The minimum inhibitory concentrations (MICs) of the three compounds were found to be above 400 μg/mL, and they inhibited planktonic cell growth in a dose-dependent manner (Figure 2B–D). Specifically, compound 6f at concentrations up to 200 μg/mL only slightly delayed cell growth, whereas compounds **6j** and **6n** more significantly inhibited growth. Notably, at concentrations of 20 and 50 μg/mL, the three compounds had only minor effects on planktonic cell growth but significantly inhibited biofilm formation in the fluconazole-resistant *C. albicans* strain. These findings suggest that while the compounds may not completely eradicate *Candida* cells, they are effective in inhibiting drug-resistant biofilms, potentially reducing the risk of developing drug resistance.

**Figure 2:**
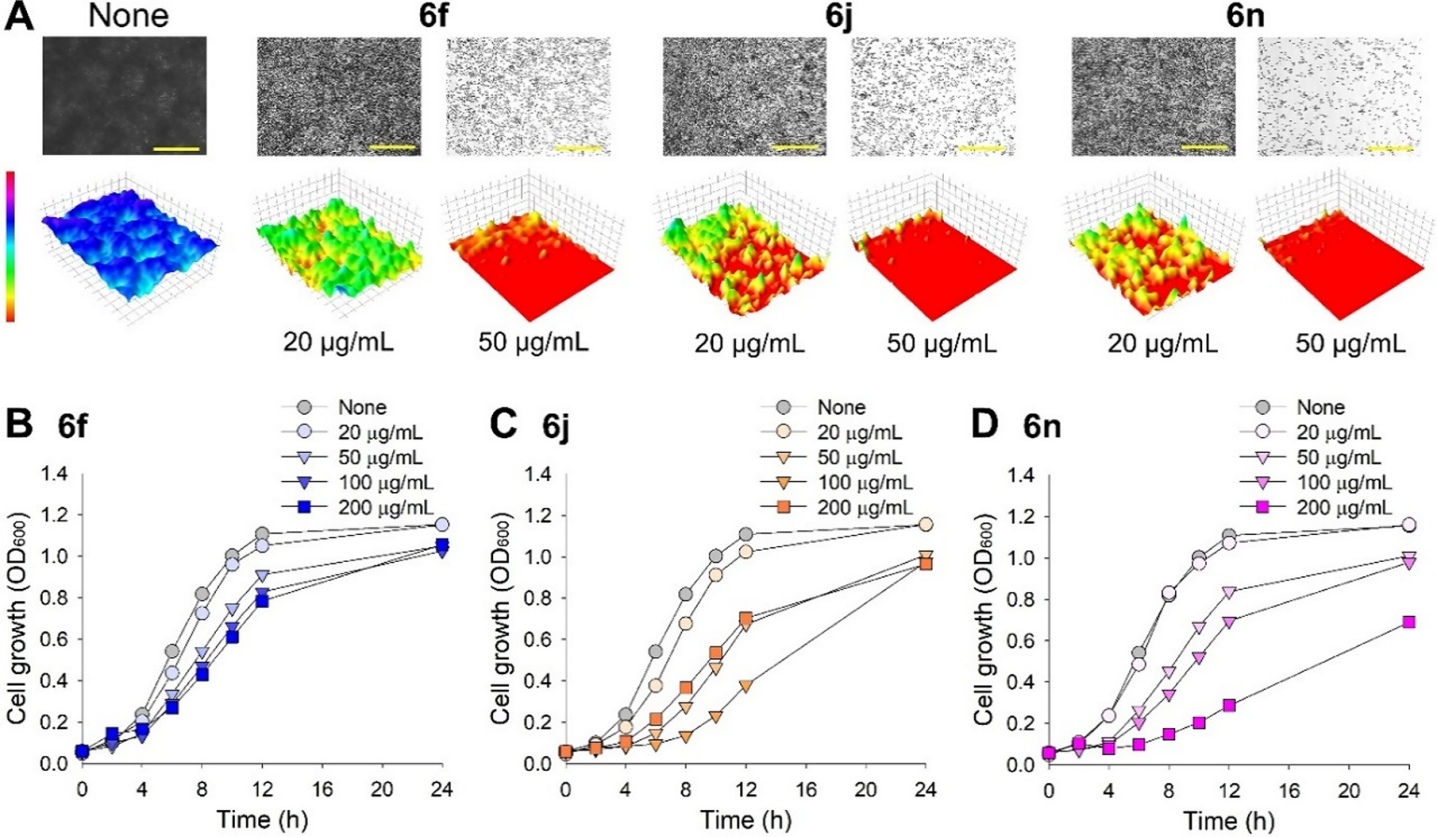
Effects of pyrone derivatives on biofilm inhibition (A) and planktonic cell growth of *C. albicans* with **6f**, **6j**, and **6n** (B–D).

Cell aggregation and the yeast-to-hyphal transition are critical prerequisites for biofilm formation in *Candida*.^31,32^ Furthermore, hyphal formation is strongly linked with increased virulence and resistance to antifungal agents.^33,34^ Therefore, the impact of pyrone derivatives on cell aggregation and hyphal formation was examined (Figure 3). As anticipated, the three active compounds dose-dependently reduced both cell aggregation (Figure 3A) and hyphal production at concentrations of 20 and 50 μg/mL (Figure 3B). These findings demonstrate that sub-MIC levels of the three pyrone derivatives effectively inhibit biofilm formation by suppressing both cell aggregation and hyphal development.

**Figure 3:**
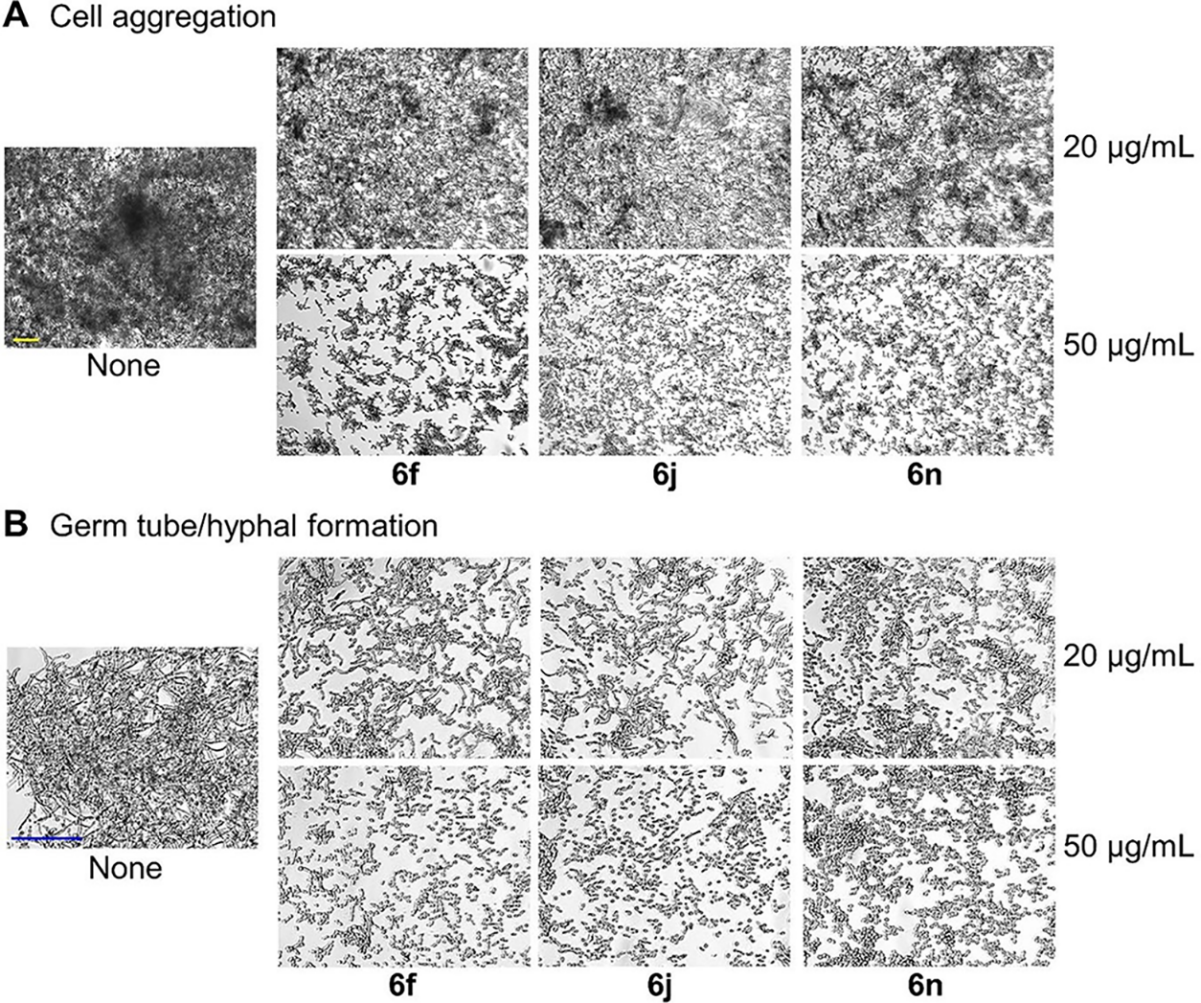
Inhibition of *C. albicans* on cell aggregation (A) and hyphae/germ tube development (B) with **6f**, **6j**, and **6n.** Both yellow and blue scale bars represent 100 μm.

### 2.5 In Silico Molecular Docking Study

ALS3, a member of the agglutinin-like sequence (ALS) family of proteins, plays a critical role in cell adhesion and biofilm formation.^35–37^ Hence, molecular docking was used to predict an interaction between the compounds and a potential molecular target—specifically, the ALS3 protein—of the synthesized pyrone derivatives, while not directly assessing cell aggregation or hyphal inhibition. The protein docking studies involving ALS3 and synthesized derivatives revealed favorable binding energies for most compounds, comparable to those of positive controls (Table 1). Specifically, derivatives **6f**, **6j**, and **6n** exhibited binding energies of −4.27, −4.37, and −4.10 kcal/mol, respectively. These values are on par with structurally similar positive controls such as atropine (−4.64 kcal/mol), diazepam (−5.48 kcal/mol), and etomidate (−4.28 kcal/mol) (Table 1) that were reported as known antifungal and antibiofilm agents.^38–42^ The three lead compounds demonstrated interactions with key residues Lys59 and Ser170, or their vicinity, which are crucial for host-peptide binding (Figure 4). This suggests that these ligands may effectively inhibit ALS3-mediated adhesion. The secondary amine group in the hit derivatives formed strong hydrogen bonds with residues Tyr23, Lys59, and Ser170. Additionally, the ketone group in the pyrone rings of the derivatives established hydrogen bonds with Asn22, Val172, and Lys59 (Figure 4). These interactions were further stabilized by the π-stacking of the phenoxy ring, as well as alkyl and halogen bonds formed by the methyl and halogen substitutions on the phenoxy ring. This interaction pattern mirrors that of the positive controls, where the keto group of the scaffold formed hydrogen bonds with Val172 or Lys59, and the phenyl ring stabilized interactions through π-stacking (Figure 4D). These findings highlight the potential of the synthesized derivatives to inhibit ALS3 function by mimicking the binding mechanisms of known positive controls.

**Table 1:** Docking Results Sorted Based on Lowest Binding Energy^a^

name	binding energy (kcal/mol)	biofilm formation (50 μg/mL, %)	biofilm formation (20 μg/mL, %)	name	binding energy (kcal/mol)	biofilm formation (50 μg/mL, %)	biofilm formation (20 μg/mL, %)
**digitalin**	–6.32	N/A	N/A	**lawsoniaside**	–4.27	n/A	n/A
**6r**	–5.72	100	100	**6b**	–4.24	8	90
**6v**	–5.63			**6j**	–4.24	2	27
**rutin**	–5.59	N/A	N/A	**etomidate**	–4.21	N/A	N/A
**diazepam**	–5.48	N/A	N/A	**6t**	–4.15	5	24
**6u**	–5.17			**6q**	–4.12	100	100
**6o**	–4.87	100	58	**6k**	–4.11	5	100
**6d**	–4.83	2	50	**6n**	–4.1	17	12
**epigallocatechin**	–4.73	N/A	N/A	**6m**	–4.02	100	50
**atropine**	–4.64	N/A	N/A	**6s**	–3.97	18	22
**6a**	–4.47	98	100	**6g**	–3.94	4	90
**6l**	–4.44	5	90	**6p**	–3.89	28	10
**6i**	–4.37	3	95	**6h**	–3.7	7	60
**6c**	–4.33	1	45	**limonene**	–3.4	N/A	N/A
**6w**	–4.32			**linalool**	–3.21	N/A	N/A
**6e**	–4.31	1	60	**eicosane**	–2.48	N/A	N/A
**6f**	–4.27	2	20				

a Diazepam, atropine, and etomidate were used as positive controls.

**Figure 4:**
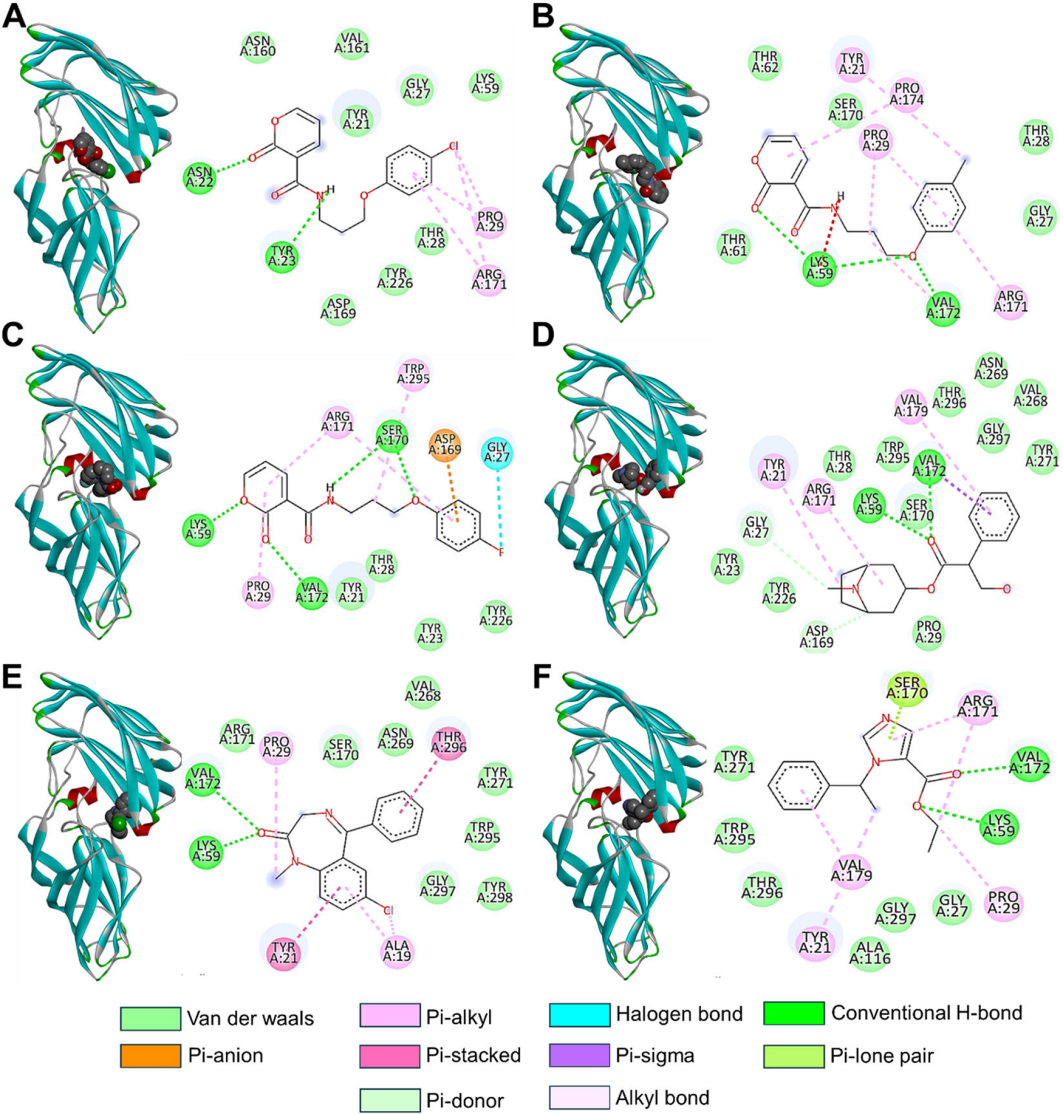
3D and 2D ALS3 protein–ligand interactions of **6f** (A), **6j** (B), **6n** (C), atropine (D), diazepam (E), and etomidate (F). Diazepam, atropine, and etomidate were used as positive controls.

Biofilm and cell growth assays revealed that derivatives **6f, 6j** and **6n** exhibit strong antibiofilm and antihyphal activities without impacting planktonic cell growth (Figure 1). This suggests that these derivatives may target pathways specific to biofilm and hyphae proliferation. Pyrones have been shown to inhibit biofilm formation in few pathogens (e.g., *S. aureus*, *P. aeruginosa* ) by disrupting adhesion mechanism.^27,43^ Similarly, pyrone-containing compounds such as flavonoids and coumarins have been reported to inhibit biofilm formation and hyphal proliferation in *C. albicans* by targeting the ALS3 adhesin.^44^ Recent studies have demonstrated that pyrrolo­[2,3-d]­pyrimidine derivatives also exhibit antibiofilm activity against *C. albicans* , suggesting their potential to interfere with adhesion-related mechanisms.^45^ Based on these findings and above references, we hypothesized that the novel pyrone derivatives may target ALS3. Molecular docking analysis predicted that these derivatives may bind to the N-terminal peptide-binding cavity of the ALS3 protein, interacting with key residues Lys59 and Ser170 with favorable binding energy (Figure 4 and Table 1). This indicates their potential to competitively inhibit the binding of host peptides to ALS3. Structural modifications to the pyrone ring, such as the incorporation of secondary amine groups, phenoxy rings, and methyl or halogen substitutions, contributed to enhanced binding stability. While these findings highlight the potential of pyrone derivatives as ALS3 inhibitors, further in vitro studies are necessary to confirm their binding and validate their mechanism of action.^46^

## 3 Conclusions

In this study, we systematically investigated a series of novel α-pyrone derivatives (**6a**–**6t**) that were designed to inhibit *C. albicans* biofilm formation and hyphal transition. Microscopic observations and antifungal assays revealed that at a concentration of 20 μg/mL, compounds **6f**, **6j**, and **6n** suppressed approximately 80, 73, and 88% of biofilm formation, respectively. Notably, at 50 μg/mL, these derivatives reduced biofilm formation by more than 83% while inhibiting planktonic cell growth by less than 38%. This profile suggests potential clinical utility for managing drug-resistant *C. albicans* strains. Molecular docking results indicated that the compounds **6f**, **6j**, and **6n** interact with key amino acids such as Lys59 and Ser170 within the ALS3 protein. These residues are crucial for the binding of host peptides, and the interactions of the derivatives primarily involved hydrogen bonding and π-stacking. Overall, these α-pyrone derivatives (**6f, 6j, 6n**) provide a promising approach to overcome the limitations associated with existing antifungal monotherapies by targeting fundamental factors of *C. albicans* biofilm formation and hyphal development. Future studies will include a comprehensive statistical evaluation of the molecular descriptors. Specifically, correlation analyses or principal component analysis can assess which structural features contribute most significantly to antibiofilm activity. Further in vitro and in vivo studies are necessary to validate the interaction of the lead compounds with ALS3 and to investigate their efficacy in animal models of *C. albicans* infection. Additionally, structure-based optimization of the pyrone scaffold and the evaluation of potential synergistic effects with conventional antifungals are suggested as follow-up studies.

## 4 Experimental Section

All reagents and analytical-grade solvents were procured from commercial suppliers and utilized without additional purification unless explicitly stated otherwise. The progress of the reaction was monitored through thin-layer chromatography (TLC), which was conducted on precoated 60F_254_ (Merck; Darmstadt, Germany) silica gel plates and subsequently visualized under ultraviolet light at wavelengths of 254 or 365 nm. The nuclear magnetic resonance (NMR) spectra of the compounds, specifically ^1^H NMR and ^13^C NMR, were obtained at ambient temperature utilizing a Bruker Ultrashield 600 MHz Plus spectrometer. Chemical shifts (δ) are reported in parts per million (ppm), with respect to tetramethylsilane or the residual peaks of the corresponding deuterated solvents. (CDCl_3_: δ 7.26 for ^1^H NMR, δ 77.16 for ^13^C NMR; DMSO-*d*_6_: δ 2.50 for 1H NMR, δ 39.52 for ^13^C NMR) The abbreviations employed to denote signal multiplicities encompass s (singlet), brs (broad singlet), d (doublet), t (triplet), q (quartet), m (multiplet), dd (doublet of doublets), among others. High-resolution mass spectra (HRMS) were obtained on an Agilent 6530 Accurate Mass Q-TOF LC/MS spectrometer. The purity of all final compounds was measured by analytical RP-HPLC on an Agilent 1260 Infinity (Agilent) with C18 column (Phenomenex,150 mm × 4.6 mm, 3 μm, 110 Å) using water (containing 0.1% TFA) and acetonitrile (ACN; containing 0.1% TFA) as the mobile phase. All compounds were monitored at a UV detector: 220 or 245 nm. The purities of the analyzed compounds were determined to be greater than 95%.

### 4.1 *tert-*Butyl (3-bromopropyl)­carbamate (**1**)

To a stirred solution of 3-bromopropylamine hydrobromide (5.0 g, 22.8 mmol) in MeOH (50 mL) at 0 °C, Boc_2_O (1.2 equiv) was added dropwise at the same temperature. The reaction mixture was vigorously stirred at room temperature until TLC analysis indicated complete conversion (typically 6 h). The mixture was then concentrated under reduced pressure and extracted with EtOAc (3 × 50 mL). The organic layer was washed with saturated brine, dried over MgSO_4_, filtered, and further concentrated under reduced pressure. Without any further purification, compound **1** was obtained as a colorless oil (4.98 g, yield: 85.0%).^1^H NMR (600 MHz, CDCl_3_) δ 4.67 (s, 1H), 3.43 (t, *J* = 6.5 Hz, 2H), 3.26 (d, *J* = 6.5 Hz, 2H), 2.07–2.00 (m, 2H), 1.43 (s, 9H).

### 4.2 General Procedure A for the Preparation of **2b–2w**

To a solution of the corresponding phenols (1.0 equiv) in DMF, K_2_CO_3_ or Cs_2_CO_3_ (2.0 equiv) was added at room temperature. After stirring for 10 min, *tert*-butyl (3-bromopropyl)­carbamate (1.5 equiv) was slowly added, and the mixture was stirred for 24 h at 60 °C. After the reaction was completed (by TLC), the mixture was extracted with EtOAc (3 × 50 mL), washed with saturated brine, and dried over MgSO_4_. Upon solvent concentration in vacuo, the residue was purified by column chromatography (Hexane/EtOAc = 9.5/0.5 to 9/1, v/v) to yield the corresponding solid.

#### 4.2.1 *tert-*Butyl (3-phenoxypropyl)­carbamate (**2b**)

Compound **2b** (300 mg, 3.18 mmol) was prepared in 53.8% yield as a colorless oil, by following the same method as described in the general procedure A with K_2_CO_3_. *R*_f_ = 0.3 (Hexane/EtOAc = 9/1, v/v).^1^H NMR (600 MHz, CDCl_3_) δ 7.30–7.27 (m, 2H), 6.95 (t, *J* = 7.3 Hz, 1H), 6.91–6.87 (m, 2H), 4.77 (s, 1H), 4.02 (t, *J* = 6.0 Hz, 2H), 3.33 (d, *J* = 6.5 Hz, 2H), 1.98 (t, *J* = 6.3 Hz, 2H), 1.44 (s, 9H).

#### 4.2.2 *tert-*Butyl (3-(3-bromophenoxy)­propyl)­carbamate (**2c**)

Compound **2c** (300 mg, 1.73 mmol) was prepared in 68.6% yield as a colorless oil, by following the same method as described in the general procedure A with K_2_CO_3_. *R*_f_ = 0.40 (Hexane/EtOAc = 9/1, v/v). ^1^H NMR (600 MHz, CDCl_3_) δ 7.13 (t, *J* = 8.1 Hz, 1H), 7.09–7.03 (m, 2H), 6.83–6.80 (m, 1H), 4.76 (s, 1H), 3.99 (d, *J* = 6.0 Hz, 2H), 3.32 (d, *J* = 6.5 Hz, 2H), 2.00–1.93 (m, 2H), 1.44 (s, 9H).

#### 4.2.3 *tert-*Butyl (3-(4-bromophenoxy)­propyl)­carbamate (**2d**)

Compound **2d** (300 mg, 1.73 mmol) was prepared in 68.6% yield as a colorless oil, by following the same method as described in the general procedure A with K_2_CO_3_. *R*_f_ = 0.58 (Hexane/EtOAc = 9/1, v/v). ^1^H NMR (600 MHz, CDCl_3_) δ 7.36 (d, *J* = 6.5 Hz, 2H), 6.77 (d, *J* = 6.5 Hz, 2H), 4.74 (s, 1H), 4.02–3.92 (m, 2H), 3.32 (d, *J* = 6.8 Hz, 2H), 1.97 (s, 2H), 1.44 (d, *J* = 2.4 Hz, 9H).

#### 4.2.4 *tert-*Butyl (3-(3-chlorophenoxy)­propyl)­carbamate (**2e**)

Compound **2e** (300 mg, 2.33 mmol) was prepared in 89.4% yield as a white solid, by following the same method as described in the general procedure A with K_2_CO_3_. *R*_f_ = 0.58 (Hexane/EtOAc = 9/1, v/v). ^1^H NMR (600 MHz, CDCl_3_) δ 7.19 (t, *J* = 8.1 Hz, 1H), 6.93 (dt, *J* = 8.1, 0.9 Hz, 1H), 6.89 (t, *J* = 2.2 Hz, 1H), 6.80–6.75 (m, 1H), 4.00 (t, *J* = 6.0 Hz, 2H), 3.32 (q, *J* = 6.5 Hz, 2H), 1.97 (p, *J* = 6.4 Hz, 2H), 1.44 (s, 9H).

#### 4.2.5 *tert-*Butyl (3-(4-chlorophenoxy)­propyl)­carbamate (**2f**)

Compound **2f** (300 mg, 02.33 mmol) was prepared in 82.5% yield as a white solid, by following the same method as described in the general procedure A with K_2_CO_3_. *R*_f_ = 0.48 (Hexane/EtOAc = 9/1, v/v). ^1^H NMR (600 MHz, CDCl_3_) δ 7.22 (d, *J* = 8.9 Hz, 2H), 6.81 (d, *J* = 8.9 Hz, 2H), 4.73 (s, 1H), 3.98 (t, *J* = 6.0 Hz, 2H), 3.32 (d, *J* = 8.2 Hz, 2H), 1.97 (p, *J* = 6.5 Hz, 2H), 1.44 (s, 9H).

#### 4.2.6 *tert-*Butyl (3-(3-(trifluoromethyl)­phenoxy)­propyl)­carbamate (**2g**)

Compound **2g** (300 mg, 0.94 mmol) was prepared in 83.4% yield as a white solid, by following the same method as described in the general procedure A with K_2_CO_3_. *R*_f_ = 0.50 (Hexane/EtOAc = 9/1, v/v). ^1^H NMR (600 MHz, CDCl_3_) δ 7.38 (t, *J* = 8.0 Hz, 1H), 7.22–7.18 (m, 1H), 7.12 (s, 1H), 7.06 (dd, *J* = 8.5, 2.5 Hz, 1H), 4.71 (s, 1H), 4.05 (t, *J* = 6.0 Hz, 2H), 3.33 (s, 2H), 2.00 (d, *J* = 6.4 Hz, 2H), 1.44 (s, 9H).

#### 4.2.7 *tert-*Butyl (3-(4-(trifluoromethyl)­phenoxy)­propyl)­carbamate (**2h**)

Compound **2h** (339.8 mg, 1.06 mmol) was prepared in 57.5% yield as a colorless oil, by following the same method as described in the general procedure A with K_2_CO_3_. *R*_f_ = 0.58 (Hexane/EtOAc = 9/1, v/v). ^1^H NMR (600 MHz, CDCl_3_) δ 7.54 (d, *J* = 8.7 Hz, 2H), 6.95 (d, *J* = 8.7 Hz, 2H), 4.71 (s, 1H), 4.06 (t, *J* = 6.0 Hz, 2H), 3.33 (d, *J* = 6.5 Hz, 2H), 2.00 (t, *J* = 6.4 Hz, 2H), 1.44 (s, 9H).

#### 4.2.8 *tert-*Butyl (3-(m-tolyloxy)­propyl)­carbamate (**2i**)

Compound **2i** (401.5 mg, 1.51 mmol) was prepared in 54.5% yield as a white solid, by following the same method as described in the general procedure A with K_2_CO_3_. *R*_f_ = 0.10 (Hexane/EtOAc = 9.5/0.5, v/v). ^1^H NMR (600 MHz, CDCl_3_) δ 7.16 (t, *J* = 7.8 Hz, 1H), 6.78–6.75 (m, 1H), 6.72 (s, 1H), 6.70 (d, *J* = 8.1 Hz, 1H), 4.78 (s, 1H), 4.00 (t, *J* = 6.0 Hz, 2H), 3.32 (d, *J* = 6.4 Hz, 2H), 2.32 (s, 3H), 1.99–1.94 (m, 2H), 1.44 (s, 9H).

#### 4.2.9 *tert-*Butyl (3-(p-tolyloxy)­propyl)­carbamate (**2j**)

Compound **2j** (463.9 mg, 1.74 mmol) was prepared in 63.0% yield as a white solid, by following the same method as described in the general procedure A with K_2_CO_3_. *R*_f_ = 0.10 (Hexane/EtOAc = 9.5/0.5, v/v). ^1^H NMR (600 MHz CDCl_3_) δ 7.07 (d, *J* = 8.3 Hz, 2H), 6.79 (d, *J* = 8.3 Hz, 2H), 4.78 (s, 1H), 3.99 (s, 2H), 3.32 (d, *J* = 6.5 Hz, 2H), 2.28 (s, 3H), 2.00–1.91 (m, 2H), 1.44 (s, 9H).

#### 4.2.10 *tert-*Butyl (3-(3-methoxyphenoxy)­propyl)­carbamate (**2k**)

Compound **2k** (493.1 mg, 1.75 mmol) was prepared in 83.4% yield as a white solid, by following the same method as described in the general procedure A with K_2_CO_3_. *R*_f_ = 0.45 (Hexane/EtOAc = 9/1, v/v). ^1^H NMR (600 MHz, CDCl_3_) δ 7.17 (t, *J* = 8.2 Hz, 1H), 6.50 (ddd, *J* = 10.1, 7.9, 2.3 Hz, 2H), 6.46 (t, *J* = 2.3 Hz, 1H), 4.76 (s, 1H), 4.00 (t, *J* = 6.0 Hz, 2H), 3.79 (s, 3H), 3.32 (d, *J* = 6.5 Hz, 2H), 1.97 (t, *J* = 6.4 Hz, 2H), 1.44 (s, 9H).

#### 4.2.11 *tert-*Butyl (3-(4-methoxyphenoxy)­propyl)­carbamate (**2l**)

Compound **2l** (434.4 mg, 1.54 mmol) was prepared in 63.8% yield as a white solid, by following the same method as described in the general procedure A with K_2_CO_3_. *R*_f_ = 0.20 (Hexane/EtOAc = 9/1, v/v). ^1^H NMR (600 MHz, CDCl_3_) δ 6.83 (s, 4H), 4.77 (s, 1H), 3.97 (t, *J* = 5.9 Hz, 2H), 3.77 (s, 3H), 3.32 (q, *J* = 6.5 Hz, 2H), 1.95 (q, *J* = 6.5 Hz, 2H), 1.44 (s, 9H).

#### 4.2.12 *tert-*Butyl (3-(3-fluorophenoxy)­propyl)­carbamate (**2m**)

Compound **2m** (938.1 mg, 3.48 mmol) was prepared in 78.1% yield as a white solid, by following the same method as described in the general procedure A with Cs_2_CO_3_. *R*_f_ = 0.58 (Hexane/EtOAc = 9/1, v/v). ^1^H NMR (600 MHz, CDCl_3_) δ 7.20 (td, *J* = 8.2, 6.8 Hz, 1H), 6.65 (qd, *J* = 8.4, 2.4 Hz, 2H), 6.60 (dt, *J* = 10.9, 2.4 Hz, 1H), 4.75 (s, 1H), 3.99 (t, *J* = 6.0 Hz, 2H), 3.32 (d, *J* = 6.5 Hz, 2H), 2.00–1.92 (m, 2H), 1.44 (s, 9H).

#### 4.2.13 *tert-*Butyl (3-(4-fluorophenoxy)­propyl)­carbamate (**2n**)

Compound **2n** (827.5 mg, 03.07 mmol) was prepared in 68.9% yield as a white solid, by following the same method as described in the general procedure A with Cs_2_CO_3_. *R*_f_ = 0.58 (Hexane/EtOAc = 9/1, v/v). ^1^H NMR (600 MHz, CDCl_3_) δ 6.96 (dd, *J* = 9.2, 8.2 Hz, 2H), 6.82 (dd, *J* = 9.2, 4.3 Hz, 2H), 4.75 (s, 1H), 3.97 (t, *J* = 6.0 Hz, 2H), 3.32 (d, *J* = 6.5 Hz, 2H), 1.96 (t, *J* = 6.4 Hz, 2H), 1.44 (s, 9H).

#### 4.2.14 *tert-*Butyl (3-(3,4-dichlorophenoxy)­propyl)­carbamate (**2o**)

Compound **2o** (689.3 mg, 2.15 mmol) was prepared in 70.1% yield as a white solid, by following the same method as described in the general procedure A with Cs_2_CO_3_. Rf = 0.20 (Hexane/EtOAc = 9/1, v/v). ^1^H NMR (600 MHz, CDCl_3_) δ 7.29 (d, *J* = 8.9 Hz, 1H), 6.97 (d, *J* = 3.0 Hz, 1H), 6.73 (dd, *J* = 8.9, 3.0 Hz, 1H), 4.75 (s, 1H), 3.96 (t, *J* = 6.0 Hz, 2H), 3.29 (t, *J* = 6.7 Hz, 2H), 1.98–1.91 (m, 2H), 1.43 (s, 9H).

#### 4.2.15 *tert-*Butyl (3-(3,4-difluorophenoxy)­propyl)­carbamate (**2p**)

Compound **2p** (740.8 mg, 2.58 mmol) was prepared in 67.0% yield as a white solid, by following the same method as described in the general procedure A with Cs_2_CO_3_. *R*_f_ = 0.20 (Hexane/EtOAc = 9/1, v/v). ^1^H NMR (600 MHz, CDCl_3_) δ 7.05 (q, *J* = 9.3 Hz, 1H), 6.70 (ddd, *J* = 12.0, 6.6, 3.0 Hz, 1H), 6.58 (dd, *J* = 9.3, 1.8 Hz, 1H), 4.70 (s, 1H), 3.96 (t, *J* = 6.0 Hz, 2H), 3.31 (q, *J* = 6.6 Hz, 2H), 1.98–1.92 (m, 2H), 1.44 (s, 9H).

#### 4.2.16 *tert-*Butyl (3-(4-chloro-3-(trifluoromethyl)­phenoxy)­propyl)­carbamate (**2q**)

Compound **2q** (558.0 mg, 1.58 mmol) was prepared in 62.7% yield as a white solid, by following the same method as described in the general procedure A with Cs_2_CO_3_. *R*_f_ = 0.20 (Hexane/EtOAc = 9/1, v/v). ^1^H NMR (600 MHz, CDCl_3_) δ 7.37 (d, *J* = 8.8 Hz, 1H), 7.19 (d, *J* = 3.0 Hz, 1H), 6.97 (dd, *J* = 8.8, 3.0 Hz, 1H), 4.70 (s, 1H), 4.02 (t, *J* = 6.0 Hz, 2H), 3.31 (s, 2H), 1.98 (q, *J* = 6.4 Hz, 2H), 1.43 (s, 9H).

#### 4.2.17 *tert-*Butyl (3-(4-nitro-3-(trifluoromethyl)­phenoxy)­propyl)­carbamate (**2r**)

Compound **2r** (635.5 mg, 1.74 mmol) was prepared in 72.3% yield as a white solid, by following the same method as described in the general procedure A with Cs_2_CO_3_. *R*_f_ = 0.20 (Hexane/EtOAc = 9/1, v/v). ^1^H NMR (600 MHz, CDCl_3_) δ 8.01 (d, *J* = 9.0 Hz, 1H), 7.29 (d, *J* = 2.7 Hz, 1H), 7.10 (dd, *J* = 9.0, 2.7 Hz, 1H), 4.67 (s, 1H), 4.14 (t, *J* = 6.1 Hz, 2H), 3.34 (d, *J* = 6.6 Hz, 2H), 2.04 (q, *J* = 6.4 Hz, 2H), 1.44 (s, 9H).

#### 4.2.18 *tert-*Butyl (3-(3,5-difluorophenoxy)­propyl)­carbamate (**2s**)

Compound **2s** (973.6 mg, 3.39 mmol) was prepared in 88.1% yield as a white solid, by following the same method as described in the general procedure A with Cs_2_CO_3_. *R*_f_ = 0.20 (Hexane/EtOAc = 9/1, v/v). ^1^H NMR (600 MHz, CDCl_3_) δ 6.43–6.36 (m, 3H), 4.69 (s, 1H), 3.98 (t, *J* = 6.0 Hz, 2H), 3.31 (d, *J* = 6.6 Hz, 2H), 1.97 (t, *J* = 6.3 Hz, 2H), 1.44 (s, 9H).

#### 4.2.19 *tert-*Butyl (3-(2,4-difluorophenoxy)­propyl)­carbamate (**2t**)

Compound **2t** (956.2 mg, 3.33 mmol) was prepared in 86.6% yield as a colorless oil, by following the same method as described in the general procedure A with Cs_2_CO_3_. *R*_f_ = 0.40 (Hexane/EtOAc = 9/1, v/v). ^1^H NMR (600 MHz, CDCl_3_) δ 6.90 (td, *J* = 9.2, 5.3 Hz, 1H), 6.84 (ddd, *J* = 11.2, 8.3, 3.0 Hz, 1H), 6.79–6.73 (m, 1H), 4.86 (s, 1H), 4.04 (t, *J* = 6.0 Hz, 2H), 3.33 (d, *J* = 6.5 Hz, 2H), 1.98 (t, *J* = 6.3 Hz, 2H), 1.43 (s, 9H).

### 4.3 General Procedure B for the Preparation of **3b–3w**

To a stirred solution of compounds **2a–2w** in DCM (5 mL), TFA (5 equiv) was added. The reaction mixture was stirred for 6 h at room temperature. After the reaction was completed, as indicated by TLC, it was concentrated under reduced pressure. Without any further purification, compounds **3b–3w** were directly used in the subsequent reaction.

#### 4.3.1 3-Phenoxypropan-1-amine (**3b**)

Compound **3b** (0.56 mmol) was generated in situ as a colorless oil, following the method described in general procedure B with **2b**. *R*_f_ = 0.05 (Hexane/EtOAc = 7/3, v/v). ^1^H NMR (600 MHz, CDCl_3_) δ 7.31 (dq, *J* = 16.5, 7.5 Hz, 3H), 7.03 (dq, *J* = 15.4, 7.5 Hz, 1H), 6.91 (dt, *J* = 17.3, 8.3 Hz, 2H), 4.18 (dq, *J* = 17.3, 5.9 Hz, 2H), 3.36 (tt, *J* = 11.9, 6.9 Hz, 2H), 2.21 (dq, *J* = 11.9, 6.9 Hz, 2H).

#### 4.3.2 3-(3-Bromophenoxy)­propan-1-amine (**3c**)

Compound **3c** (0.99 mmol) was generated in situ as a colorless oil, following the method described in general procedure B with **2c**. *R*_f_ = 0.05 (Hexane/EtOAc = 9/1, v/v). ^1^H NMR (600 MHz, CDCl_3_) δ 7.14–7.10 (m, 2H), 7.04–7.02 (m, 1H), 6.81 (dq, *J* = 8.2, 2.8 Hz, 1H), 4.10 (t, *J* = 5.5 Hz, 2H), 3.30 (h, *J* = 5.8 Hz, 2H), 2.22–2.15 (m, 2H).

#### 4.3.3 3-(4-Bromophenoxy)­propan-1-amine (**3d**)

Compound **3d** (0.93 mmol) was generated in situ as a colorless oil, following the method described in general procedure B with **2d**. *R*_f_ = 0.02 (Hexane/EtOAc = 3/7, v/v). ^1^H NMR (600 MHz, CDCl_3_) δ 7.39–7.35 (m, 2H), 6.78–6.73 (m, 2H), 4.09 (t, *J* = 5.5 Hz, 2H), 3.30 (h, *J* = 6.0 Hz, 2H), 2.18 (p, *J* = 6.0 Hz, 2H).

#### 4.3.4 3-(3-chlorophenoxy)­propan-1-amine (**3e**)

Compound **3e** (1.12 mmol) was generated in situ as a colorless oil, following the method described in general procedure B with **2e**. *R*_f_ = 0.02 (Hexane/EtOAc = 3/7, v/v). ^1^H NMR (600 MHz, CDCl_3_) δ 7.19 (t, *J* = 8.2 Hz, 1H), 6.97 (dd, *J* = 8.0, 1.9 Hz, 1H), 6.88 (t, *J* = 2.2 Hz, 1H), 6.76 (dd, *J* = 8.2, 2.5 Hz, 1H), 4.11 (t, *J* = 5.5 Hz, 2H), 3.32 (h, *J* = 5.9 Hz, 2H), 2.19 (p, *J* = 6.0 Hz, 2H).

#### 4.3.5 3-(4-Chlorophenoxy)­propan-1-amine (**3f**)

Compound **3f** (1.12 mmol) was generated in situ as a colorless oil, following the method described in general procedure B with **2f**. *R*_f_ = 0.02 (Hexane/EtOAc = 3/7, v/v). ^1^H NMR (600 MHz, CDCl_3_) δ 7.23 (d, *J* = 8.9 Hz, 2H), 6.80 (d, *J* = 8.9 Hz, 2H), 4.10 (t, *J* = 5.5 Hz, 2H), 3.33 (q, *J* = 6.0 Hz, 2H), 2.21–2.15 (m, 2H).

#### 4.3.6 3-(3-(Trifluoromethyl)­phenoxy)­propan-1-amine (**3g**)

Compound **3g** (1.09 mmol) was generated in situ as a colorless oil, following the method described in general procedure B with **2g**. *R*_f_ = 0.02 (Hexane/EtOAc = 3/7, v/v). ^1^H NMR (600 MHz, CDCl_3_) δ 7.37 (t, *J* = 8.0 Hz, 1H), 7.23 (d, *J* = 7.7 Hz, 1H), 7.09 (s, 1H), 7.03 (dd, *J* = 8.4, 2.6 Hz, 1H), 4.13 (t, *J* = 5.5 Hz, 2H), 3.33–3.25 (m, 2H), 2.20 (p, *J* = 6.1 Hz, 2H).

#### 4.3.7 3-(4-(Trifluoromethyl)­phenoxy)­propan-1-amine (**3h**)

Compound **3h** (1.03 mmol) was generated in situ as a colorless oil, following the method described in general procedure B with **2h**. *R*_f_ = 0.02 (Hexane/EtOAc = 3/7, v/v). ^1^H NMR (600 MHz, CDCl_3_) δ 7.54 (d, *J* = 8.6 Hz, 2H), 6.94 (d, *J* = 8.6 Hz, 2H), 4.18 (t, *J* = 5.5 Hz, 2H), 3.34 (q, *J* = 6.1 Hz, 2H), 2.26–2.20 (m, 2H).

#### 4.3.8 3-(*m*-Tolyloxy)­propan-1-amine (**3i**)

Compound **3i** (1.13 mmol) was generated in situ as a colorless oil, following the method described in general procedure B with **2i**. *R*_f_ = 0.02 (Hexane/EtOAc = 3/7, v/v). ^1^H NMR (600 MHz, CDCl_3_) δ 7.16 (d, *J* = 7.8 Hz, 1H), 6.83 (d, *J* = 7.5 Hz, 1H), 6.72 (t, *J* = 2.0 Hz, 1H), 6.68 (dd, *J* = 8.2, 2.6 Hz, 1H), 4.15 (t, *J* = 5.4 Hz, 2H), 3.35 (q, *J* = 5.9 Hz, 2H), 2.31 (s, 3H), 2.21–2.15 (m, 2H).

#### 4.3.9 3-(*p*-Tolyloxy)­propan-1-amine (**3j**)

Compound **3j** (1.13 mmol) was generated in situ as a colorless oil, following the method described in general procedure B with **2j**. *R*_f_ = 0.02 (Hexane/EtOAc = 3/7, v/v). ^1^H NMR (600 MHz, CDCl_3_) δ 7.08 (d, *J* = 8.6 Hz, 2H), 6.78 (d, *J* = 8.6 Hz, 2H), 4.15–4.08 (m, 2H), 3.34 (dd, *J* = 6.1, 2.8 Hz, 2H), 2.28 (s, 3H), 2.20–2.13 (m, 2H).

#### 4.3.10 3-(3-Methoxyphenoxy)­propan-1-amine (**3k**)

Compound **3k** (1.10 mmol) was generated in situ as a colorless oil, following the method described in general procedure B with **2k**. *R*_f_ = 0.02 (Hexane/EtOAc = 3/7, v/v). ^1^H NMR (600 MHz, CDCl_3_) δ 7.18 (t, *J* = 8.3 Hz, 1H), 6.58–6.56 (m, 1H), 6.49–6.47 (m, 1H), 6.45 (s, 1H), 4.14 (t, *J* = 5.5 Hz, 2H), 3.77 (d, *J* = 0.9 Hz, 3H), 3.35 (q, *J* = 5.9 Hz, 2H), 2.19 (p, *J* = 5.7 Hz, 2H).

#### 4.3.11 3-(4-Methoxyphenoxy)­propan-1-amine (**3l**)

Compound **3l** (1.10 mmol) was generated in situ as a colorless oil, following the method described in general procedure B with **2l**. *R*_f_ = 0.02 (Hexane/EtOAc = 4/6, v/v). ^1^H NMR (600 MHz, CDCl_3_) δ 6.83 (s, 4H), 4.12 (t, *J* = 5.5 Hz, 2H), 3.77 (s, 3H), 3.36 (q, *J* = 5.9 Hz, 2H), 2.17 (p, *J* = 5.7 Hz, 2H).

#### 4.3.12 3-(3-Fluorophenoxy)­propan-1-amine (**3m**)

Compound **3m** (1.11 mmol) was generated in situ as a colorless oil l, following the method described in general procedure B with **2m**. *R*_f_ = 0.02 (Hexane/EtOAc = 4/6, v/v). ^1^H NMR (600 MHz, CDCl_3_) δ 7.22 (td, *J* = 8.3, 6.7 Hz, 1H), 6.69 (td, *J* = 8.3, 2.4 Hz, 1H), 6.66 (dd, *J* = 8.3, 2.5 Hz, 1H), 6.59 (dt, *J* = 10.7, 2.4 Hz, 1H), 4.11 (t, *J* = 5.5 Hz, 2H), 3.32 (q, *J* = 6.0 Hz, 2H), 2.23–2.12 (m, 2H).

#### 4.3.13 3-(4-Fluorophenoxy)­propan-1-amine (**3n**)

Compound **3n** (1.11 mmol) was generated in situ as a colorless oil following the method described in general procedure B with **2n**. *R*_f_ = 0.02 (Hexane/EtOAc = 3/7, v/v). ^1^H NMR (600 MHz, CDCl_3_) δ 6.96 (dd, *J* = 9.1, 8.1 Hz, 2H), 6.82 (dd, *J* = 9.1, 4.2 Hz, 2H), 4.09 (t, *J* = 5.5 Hz, 2H), 3.31 (q, *J* = 5.9 Hz, 2H), 2.21–2.12 (m, 2H).

#### 4.3.14 3-(3,4-Dichlorophenoxy)­propan-1-amine (**3o**)

Compound **3o** (1.09 mmol) was generated in situ as a colorless oil, following the method described in general procedure B with **2o**. *R*_f_ = 0.02 (Hexane/EtOAc = 3/7, v/v). ^1^H NMR (600 MHz, CDCl_3_) δ 7.31 (d, *J* = 8.8 Hz, 1H), 6.98 (d, *J* = 2.9 Hz, 1H), 6.73 (dd, *J* = 8.8, 2.9 Hz, 1H), 4.06 (s, 2H), 3.23 (s, 2H), 2.21–2.13 (m, 2H).

#### 4.3.15 3-(3,4-Difluorophenoxy)­propan-1-amine (**3p**)

Compound **3p** (1.09 mmol) was generated in situ as a colorless oil, following the method described in general procedure B with **2p**. *R*_f_ = 0.02 (Hexane/EtOAc = 3/7, v/v). ^1^H NMR (600 MHz, CDCl_3_) δ 7.05 (q, *J* = 9.3 Hz, 1H), 6.70 (dq, *J* = 9.5, 3.3 Hz, 1H), 6.58 (dd, *J* = 9.3, 2.2 Hz, 1H), 4.08 (t, *J* = 5.5 Hz, 2H), 3.35–3.26 (m, 2H), 2.20–2.17 (m, 2H).

#### 4.3.16 3-(4-Chloro-3-(trifluoromethyl)­phenoxy)­propan-1-amine (**3q**)

Compound **3q** (1.36 mmol) was generated in situ as a colorless oil, following the method described in general procedure B with **2q**. *R*_f_ = 0.02 (Hexane/EtOAc = 3/7, v/v). ^1^H NMR (600 MHz, CDCl_3_) δ 7.40 (d, *J* = 8.9 Hz, 1H), 7.20 (d, *J* = 3.0 Hz, 1H), 6.98 (dd, *J* = 8.9, 3.0 Hz, 1H), 4.15 (t, *J* = 5.5 Hz, 2H), 3.33 (s, 2H), 2.26–2.19 (m, 2H).

#### 4.3.17 3-(4-Nitro-3-(trifluoromethyl)­phenoxy)­propan-1-amine (**3r**)

Compound **3r** (1.09 mmol) was generated in situ as a colorless oil, following the method described in general procedure B with **2r**. *R*_f_ = 0.02 (Hexane/EtOAc = 3/7, v/v). ^1^H NMR (600 MHz, DMSO) δ 8.21 (d, *J* = 9.0 Hz, 1H), 7.48 (d, *J* = 2.7 Hz, 1H), 7.44 (dd, *J* = 9.0, 2.7 Hz, 1H), 4.28 (t, *J* = 6.0 Hz, 2H), 3.00 (q, *J* = 6.5 Hz, 2H), 2.06 (p, *J* = 6.5 Hz, 2H).

#### 4.3.18 3-(3,5-Difluorophenoxy)­propan-1-amine (**3s**)

Compound **3s** (1.13 mmol) was generated in situ as a colorless oil, following the method described in general procedure B with **2s**. *R*_f_ = 0.02 (Hexane/EtOAc = 3/7, v/v). ^1^H NMR (600 MHz, CDCl_3_) δ 6.48–6.34 (m, 3H), 4.09 (t, *J* = 5.5 Hz, 2H), 3.30 (s, 2H), 2.20 (p, *J* = 6.1 Hz, 2H).

#### 4.3.19 3-(2,4-Difluorophenoxy)­propan-1-amine (**3t**)

Compound **3t** (1.11 mmol) was generated in situ as a colorless oil, following the method described in general procedure B with **2s**. *R*_f_ = 0.58 (Hexane/EtOAc = 8/1, v/v). ^1^H NMR (600 MHz, CDCl_3_) δ 6.92 (td, *J* = 9.1, 5.2 Hz, 1H), 6.85 (ddd, *J* = 11.1, 8.2, 3.0 Hz, 1H), 6.80 (dddd, *J* = 9.1, 7.8, 3.0, 1.7 Hz, 1H), 4.20 (t, *J* = 5.4 Hz, 2H), 3.39 (q, *J* = 5.8 Hz, 2H), 2.24–2.19 (m, 2H).

#### 4.3.20 2-Oxo-2H-pyran-3-carboxylic Acid (**4**)

To a stirred solution of methyl 2-oxo-2H-pyran-3-carboxylate (1.00 g, 6.49 mmol) in concentrated HCl (15 mL) at room temperature, the reaction mixture was stirred at 45 °C for 24 h while monitoring the progress of the reaction by TLC. The reaction was then quenched with cold water. Subsequently, the mixture was extracted with ethyl acetate (3 × 50 mL), washed with saturated brine, and dried over MgSO_4_. The crude residue was decanted from hexane to yield compound **4** (446 mg, 45% yield). ^1^H NMR (600 MHz, CDCl_3_) δ 8.56 (dd, *J* = 6.8, 2.2 Hz, 1H), 7.82 (dd, *J* = 5.0, 2.2 Hz, 1H), 6.66 (dd, *J* = 6.8, 5.0 Hz, 1H).

#### 4.3.21 2-Oxo-2H-pyran-3-carbonyl chloride (**5**)

To a solution of compound **4** (1.0 equiv) in DCM, (COCl)_2_ (2.0 equiv) was added at room temperature. Subsequently, DMF (catalytic amount) was slowly added. The mixture was stirred for 3 h at room temperature. After the completion of the reaction, as confirmed by TLC, it was used in the subsequent reaction without any further purification.

### 4.4 General Procedure C for the Preparation of **6a**–**6w**

To a stirred solution of compounds **3b–3w** or dodecylamine in DCM (5 mL), TEA (1.5 −3.0 equiv) was added at 0 °C. Compound **5** was dissolved in DCM and added dropwise at the same temperature. The mixture was then stirred at room temperature for 24 h. After the reaction was completed (by TLC), the mixture was extracted with DCM (3 × 50 mL), washed with saturated brine, and dried over MgSO_4_. Upon solvent concentration *in vacuo*, the residue was purified by column chromatography to yield the corresponding solid.

#### 4.4.1 *N*-Dodecyl-2-oxo-2H-pyran-3-carboxamide (**6a**)

Compound **6a** (42.4 mg, 0.14 mmol) was prepared as a white solid in 19.3% yield by following the method described in general procedure C with dodecylamine (198.2 mg, 1.07 mmol). *R*_f_ = 0.58 (Hexane/EtOAc = 8/1, v/v). ^1^H NMR (600 MHz, CDCl_3_) δ 8.67 (s, 1H), 8.52 (dd, *J* = 6.8, 2.3 Hz, 1H), 7.66 (dd, *J* = 5.0, 2.3 Hz, 1H), 6.50 (dd, *J* = 6.8, 5.0 Hz, 1H), 1.63–1.57 (m, 2H), 1.36 (d, *J* = 8.2 Hz, 2H), 1.25 (s, 18H), 0.88 (t, *J* = 7.0 Hz, 3H). ^13^C NMR (150 MHz, CDCl_3_): δ162.34, 161.48, 154.42, 147.50, 119.48, 114.64, 107.40, 39.98, 32.05, 29.78, 29.76, 29.72, 29.66, 29.50, 29.48, 29.42, 27.14, 22.82, 14.25. HRMS *m*/*z* calculated for C_18_H_29_NO_3_ [M + H]^+^: 307.2147; found: 308.2244. > 98% purity (as determined by RP-HPLC, *t*_R_ = 9.97 min).

#### 4.4.2 2-Oxo-*N*-(3-phenoxypropyl)-2H-pyran-3-carboxamide (**6b**)

Compound **6b** (11.2 mg, 0.36 mmol) was prepared as a white solid in 11.5% yield by following the method described in general procedure C with **3b** (80.7 mg, 0.53 mmol). *R*_f_ = 0.4 (Hexane/EtOAc = 6/4, v/v). ^1^H NMR (600 MHz, CDCl_3_) δ 8.92 (s, 1H), 8.52 (dd, *J* = 6.9, 2.3 Hz, 1H), 7.67 (dd, *J* = 5.0, 2.3 Hz, 1H), 7.30–7.27 (m, 2H), 6.97–6.91 (m, 3H), 6.51 (dd, *J* = 6.8, 5.0 Hz, 1H), 4.07 (t, *J* = 5.9 Hz, 2H), 3.65 (q, *J* = 6.4 Hz, 2H), 2.11 (p, *J* = 6.3 Hz, 2H). ^13^C NMR (150 MHz, CDCl_3_): δ162.21, 161.72, 158.92, 154.56, 147.62, 129.59, 120.95, 119.37, 114.64, 107.38, 66.01, 37.64, 29.21. HRMS *m*/*z* calculated for C_15_H_15_NO_4_ [M + H]^+^: 273.1001; found: 274.1054. > 95% purity (as determined by RP-HPLC, *t*_R_ = 6.25 min).

#### 4.4.3 *N*-(3-(3-Bromophenoxy)­propyl)-2-oxo-2H-pyran-3-carboxamide (**6c**)

Compound **6c** (90 mg, 0.64 mmol) was prepared as a white solid in 12.6% yield by following the method described in general procedure C with **3c** (221.6 mg, 0.98 mmol). *R*_f_ = 0.45 (Hexane/EtOAc = 4/6, v/v). ^1^H NMR (600 MHz, CDCl_3_) δ 8.90 (s, 1H), 8.52 (dd, *J* = 6.9, 2.3 Hz, 1H), 7.68 (dd, *J* = 5.0, 2.3 Hz, 1H), 7.13 (t, *J* = 8.0 Hz, 1H), 7.10–7.05 (m, 2H), 6.91–6.86 (m, 1H), 6.51 (dd, *J* = 6.8, 5.0 Hz, 1H), 4.04 (t, *J* = 5.9 Hz, 2H), 3.63 (q, *J* = 6.4 Hz, 2H), 2.10 (p, *J* = 6.3 Hz, 2H). ^13^C NMR (150 MHz, CDCl_3_): δ162.23, 161.75, 159.69, 154.61, 147.68, 130.68, 124.05, 122.91, 119.28, 117.79, 113.81, 107.40, 66.34, 37.45, 29.08. HRMS *m*/*z* calculated for C_15_H_14_BrNO_4_ [M + H]^+^: 351.0106; found: 352.0142. > 98% purity (as determined by RP-HPLC, *t*_R_ = 4.05 min).

#### 4.4.4 *N*-(3-(4-Bromophenoxy)­propyl)-2-oxo-2H-pyran-3-carboxamide (**6d**)

Compound **6d** (35.5 mg, 0.61 mmol) was prepared as a white solid in 19.6% yield by following the method described in general procedure C with **3d** (209.1 mg, 0.91 mmol). *R*_f_ = 0.45 (Hexane/EtOAc = 4/6, v/v). ^1^H NMR (600 MHz, CDCl_3_) δ 8.91 (s, 1H), 8.52 (dd, *J* = 6.9, 2.3 Hz, 1H), 7.68 (dd, *J* = 5.0, 2.2 Hz, 1H), 7.39–7.34 (m, 2H), 6.86–6.80 (m, 2H), 6.51 (dd, *J* = 6.9, 5.0 Hz, 1H), 4.03 (t, *J* = 5.9 Hz, 2H), 3.64 (q, *J* = 6.4 Hz, 2H), 2.10 (p, *J* = 6.3 Hz, 2H). ^13^C NMR (150 MHz, CDCl_3_): δ162.21, 161.71, 158.02, 154.60, 147.66, 132.37, 119.28, 116.44, 113.11, 107.39, 66.43, 37.53, 29.08. HRMS *m*/*z* calculated for C_15_H_14_BrNO_4_ [M + H]^+^: 351.0106; found: 352.0145. > 98% purity (as determined by RP-HPLC, *t*_R_ = 8.33 min).

#### 4.4.5 *N*-(3-(3-Chlorophenoxy)­propyl)-2-oxo-2H-pyran-3-carboxamide (**6e**)

Compound **6e** (36.3 mg, 0.71 mmol) was prepared as a white solid in 16.5% yield by following the method described in general procedure C with **3e** (198.5 mg, 1.1 mmol). *R*_f_ = 0.45 (Hexane/EtOAc = 4/6, v/v). ^1^H NMR (600 MHz, CDCl_3_) δ 8.90 (s, 1H), 8.52 (dd, *J* = 6.9, 2.2 Hz, 1H), 7.68 (dd, *J* = 5.0, 2.2 Hz, 1H), 7.19 (t, *J* = 8.3 Hz, 1H), 6.95–6.90 (m, 2H), 6.84 (dd, *J* = 8.7, 2.2 Hz, 1H), 6.51 (dd, *J* = 6.9, 5.0 Hz, 1H), 4.05 (t, *J* = 5.9 Hz, 2H), 3.64 (q, *J* = 6.4 Hz, 2H), 2.10 (p, *J* = 6.3 Hz, 2H). ^13^C NMR (150 MHz, CDCl_3_): δ162.23, 161.75, 159.66, 154.61, 147.68, 134.97, 130.35, 121.13, 119.29, 114.93, 113.30, 107.40, 66.35, 37.46, 29.09. HRMS *m*/*z* calculated for C_15_H_14_ClNO_4_ [M + H]^+^: 307.0611; found: 308.0709. > 98% purity (as determined by RP-HPLC, *t*_R_ = 6.46 min).

#### 4.4.6 *N*-(3-(4-Chlorophenoxy)­propyl)-2-oxo-2H-pyran-3-carboxamide (**6f**)

Compound **6f** (56.7 mg, 0.71 mmol) was prepared as a white solid in 25.8% yield by following the method described in general procedure C with **3f** (198.5 mg, 1.07 mmol). *R*_f_ = 0.40 (Hexane/EtOAc = 4/6, v/v. ^1^H NMR (600 MHz, CDCl_3_) δ 8.91 (s, 1H), 8.52 (dd, *J* = 6.8, 2.3 Hz, 1H), 7.68 (dd, *J* = 5.0, 2.2 Hz, 1H), 7.23 (d, *J* = 8.9 Hz, 2H), 6.87 (d, *J* = 8.9 Hz, 2H), 6.51 (dd, *J* = 6.8, 5.0 Hz, 1H), 4.03 (t, *J* = 5.9 Hz, 2H), 3.64 (q, *J* = 6.4 Hz, 2H), 2.10 (p, *J* = 6.2 Hz, 2H). ^13C^ NMR (150 MHz, CDCl_3_): δ162.33, 161.82, 157.63, 154.70, 147.77, 129.54, 125.91, 119.40, 116.03, 116.01, 107.50, 66.60, 37.66, 29.21. HRMS *m*/*z* calculated for C_15_H_14_ClNO_4_ [M + H]^+^: 307.0611; found: 308.0713. > 97% purity (as determined by RP-HPLC, *t*_R_ = 6.52 min).

#### 4.4.7 2-Oxo-*N*-(3-(4-(trifluoromethyl)­phenoxy)­propyl)-2H-pyran-3-carboxamide (**6g**)

Compound **6g** (56.3 mg, 0.99 mmol) was prepared as a white solid in an 23.1% yield by following the method described in general procedure C with **3g** (234.4 mg, 1.07 mmol). *R*_f_ = 0.45 (Hexane/EtOAc = 4/6, v/v). ^1^H NMR (600 MHz, CDCl_3_) δ 8.92 (s, 1H), 8.52 (dd, *J* = 6.9, 2.3 Hz, 1H), 7.68 (dd, *J* = 5.0, 2.3 Hz, 1H), 7.38 (t, *J* = 8.0 Hz, 1H), 7.20 (d, *J* = 7.7 Hz, 1H), 7.15 (s, 1H), 7.14–7.11 (m, 1H), 6.51 (dd, *J* = 6.8, 5.0 Hz, 1H), 4.10 (t, *J* = 5.9 Hz, 3H), 3.67–3.62 (m, 2H), 2.13 (p, *J* = 6.3 Hz, 2H). ^13^C NMR (150 MHz, CDCl_3_) δ 162.25, 161.77, 159.03, 154.63, 147.70, 132.03, 131.82, 130.10, 125.01, 123.21, 119.28, 118.29, 117.66, 117.64, 111.29, 111.26, 107.40, 66.42, 37.47, 29.08. HRMS *m*/*z* calculated for C_15_H_14_F_3_NO_4_ [M + H]^+^: 341.0875; found: 342.0940. > 99% purity (as determined by RP-HPLC, *t*_R_ = 5.87 min).

#### 4.4.8 2-Oxo-*N*-(3-(3-(trifluoromethyl)­phenoxy)­propyl)-2H-pyran-3-carboxamide (**6h**)

Compound **6h** (34.2 mg, 0.10 mmol) was prepared as a white solid in 14.8% yield by following the method described in general procedure C with **3h** (222.9 mg, 1.02 mmol). *R*_f_ = 0.30 (Hexane/EtOAc = 4/6, v/v). ^1^H NMR (600 MHz, CDCl_3_) δ 8.92 (s, 1H), 8.52 (dd, *J* = 6.8, 2.3 Hz, 1H), 7.68 (dd, *J* = 5.0, 2.3 Hz, 1H), 7.54 (d, *J* = 8.5 Hz, 2H), 7.01 (d, *J* = 8.5 Hz, 2H), 6.52 (dd, *J* = 6.8, 5.0 Hz, 1H), 4.11 (t, *J* = 5.9 Hz, 2H), 3.65 (q, *J* = 6.4 Hz, 2H), 2.13 (q, *J* = 6.3 Hz, 2H ^13^C NMR (151 MHz, CDCl_3_) δ 162.35, 161.87, 161.45, 154.74, 147.81, 127.15, 127.13, 127.10, 127.08, 125.58, 123.31, 123.09, 119.36, 114.70, 107.51, 66.52, 37.58, 29.14. HRMS *m*/*z* calculated for C_15_H_14_F_3_NO_4_ [M + H]^+^: 341.0875; found: 342.0964. > 99% purity (as determined by RP-HPLC, *t*_R_ = 5.89 min).

#### 4.4.9 2-Oxo-*N*-(3-(p-tolyloxy)­propyl)-2H-pyran-3-carboxamide (**6i**)

Compound **6i** (41.1 mg, 0.14 mmol) was prepared as a white solid in 20.0% yield by following the method described in general procedure C with **3i** (176.7 mg, 1.07 mmol). *R*_f_ = 0.40 (Hexane/EtOAc = 4/6, v/v). ^1^H NMR (600 MHz, CDCl_3_) δ 8.89 (s, 1H), 8.52 (dd, *J* = 6.9, 2.2 Hz, 1H), 7.67 (dd, *J* = 5.0, 2.2 Hz, 1H), 7.16 (t, *J* = 7.9 Hz, 1H), 6.77–6.73 (m, 3H), 6.50 (dd, *J* = 6.9, 5.0 Hz, 1H), 4.05 (t, *J* = 6.0 Hz, 2H), 3.66–3.62 (m, 2H), 2.33 (s, 3H), 2.09 (p, *J* = 6.4 Hz, 2H). ^13^C NMR (150 MHz, CDCl_3_): δ162.29, 161.81, 159.03, 154.65, 147.69, 129.41, 121.85, 119.45, 115.52, 111.69, 107,46, 65.96, 37.66, 29.33, 21.77. HRMS *m*/*z* calculated for C_16_H_17_NO_4_ [M + H]^+^: 287.1158; found: 288.1205. > 99% purity (as determined by RP-HPLC, *t*_R_ = 4.67 min).

#### 4.4.10 2-Oxo-*N*-(3-(m-tolyloxy)­propyl)-2H-pyran-3-carboxamide (**6j**)

Compound **6j** (32.9 mg, 0.11 mmol) was prepared as a white solid in 16.0% yield by following the method described in general procedure C with **3j** (176.7 mg, 1.07 mmol). *R*_f_ = 0.30 (Hexane/EtOAc = 4/6, v/v). ^1^H NMR (600 MHz, CDCl_3_) δ 8.93 (s, 1H), 8.54 (dd, *J* = 6.9, 2.2 Hz, 1H), 7.69 (dd, *J* = 5.0, 2.3 Hz, 1H), 7.10 (d, *J* = 8.4 Hz, 2H), 6.87 (d, *J* = 8.4 Hz, 2H), 6.53 (dd, *J* = 6.9, 5.0 Hz, 1H), 4.06 (t, *J* = 5.9 Hz, 2H), 3.69–3.63 (m, 2H), 2.31 (s, 3H), 2.11 (p, *J* = 6.3 Hz, 2H). ^13^C NMR (150 MHz, CDCl_3_): δ162.26, 161.78, 156.88, 154.62, 147.67, 130.09, 119.44, 114.57, 107,44, 66.23, 37.72, 29.30, 20.68. HRMS *m*/*z* calculated for C_16_H_17_NO_4_ [M + H]^+^: 287.1158; found: 288.1201. > 99% purity (as determined by RP-HPLC, *t*_R_ = 4.89 min).

#### 4.4.11 *N*-(3-(4-Methoxyphenoxy)­propyl)-2-oxo-2H-pyran-3-carboxamide (**6k**)

Compound **6k** (49.4 mg, 0.16 mmol) was prepared as a white solid in 22.8% yield by following the method described in general procedure C with **3k** (193.8 mg, 1.07 mmol). *R*_f_ = 0.30 (Hexane/EtOAc = 4/6, v/v). ^1^H NMR (600 MHz, CDCl_3_) δ 8.91 (s, 1H), 8.52 (dd, *J* = 6.8, 2.3 Hz, 1H), 7.67 (dd, *J* = 5.0, 2.2 Hz, 1H), 7.17 (t, *J* = 8.5 Hz, 1H), 6.55–6.52 (m, 2H), 6.50 (dd, *J* = 6.8, 5.0 Hz, 2H), 4.05 (t, *J* = 5.9 Hz, 2H), 3.79 (s, 3H), 3.64 (q, *J* = 6.4 Hz, 2H), 2.10 (p, *J* = 6.3 Hz, 2H). ^13^C NMR (150 MHz, CDCl_3_): δ162.17, 161.71, 160.96, 160.18, 154.55, 147.61, 129.99, 119.35, 107.36, 106.71, 106.67, 101.13, 66.12, 55.41, 37.64, 29.16. HRMS *m*/*z* calculated for C_18_H_17_NO_5_ [M + H]^+^: 303.1107; found: 304.1185. > 98% purity (as determined by RP-HPLC, *t*_R_ = 4.01 min).

#### 4.4.12 *N*-(3-(3-Methoxyphenoxy)­propyl)-2-oxo-2H-pyran-3-carboxamide (**6l**)

Compound **6l** (31.6 mg, 0.10 mmol) was prepared as a white solid in 14.6% yield by following the method described in general procedure C with **3l** (193.8 mg, 1.07 mmol). *R*_f_ = 0.30 (Hexane/EtOAc = 4/6, v/v). ^1^H NMR (600 MHz, CDCl_3_) δ 8.92 (s, 1H), 8.52 (dd, *J* = 6.9, 2.3 Hz, 1H), 7.67 (dd, *J* = 5.0, 2.3 Hz, 1H), 6.89 (d, *J* = 9.1 Hz, 2H), 6.83 (d, *J* = 9.1 Hz, 2H), 6.50 (dd, *J* = 6.9, 5.0 Hz, 1H), 4.02 (t, *J* = 5.9 Hz, 2H), 3.77 (s, 3H), 3.64 (q, *J* = 6.5 Hz, 2H), 2.08 (p, *J* = 6.3 Hz, 2H). ^13^C NMR (150 MHz, CDCl_3_): δ 162.19, 161.68, 160.96, 154.54, 154.02, 153.09, 147.59, 119.36, 115.57, 114.78, 107.36, 66.77, 55.87, 37.66, 29.25. HRMS *m*/*z* calculated for C_18_H_17_NO_5_ [M + H]^+^: 303.1107; found: 304.1189. > 99% purity (as determined by RP-HPLC, *t*_R_ = 3.70 min).

#### 4.4.13 *N*-(3-(3-Fluorophenoxy)­propyl)-2-oxo-2H-pyran-3-carboxamide (**6m**)

Compound **6m** (16.4 mg, 0.06 mmol) was prepared as a white solid in 7.9% yield by following the method described in general procedure C with **3m** (180.9 mg, 1.07 mmol). *R*_f_ = 0.60 (Hexane/EtOAc = 4/6, v/v). ^1^H NMR (600 MHz, CDCl_3_) δ 8.91 (s, 1H), 8.52 (dd, *J* = 6.9, 2.3 Hz, 1H), 7.68 (dd, *J* = 5.0, 2.3 Hz, 1H), 7.22–7.18 (m, 1H), 6.75–6.71 (m, 1H), 6.65 (dd, *J* = 9.4, 1.7 Hz, 2H), 6.51 (dd, *J* = 6.9, 5.0 Hz, 1H), 4.05 (t, *J* = 5.9 Hz, 2H), 3.64 (q, *J* = 6.4 Hz, 2H), 2.10 (p, *J* = 6.2 Hz, 2H). ^13^C NMR (150 MHz, CDCl_3_) δ 164.57, 162.95, 162.23, 161.77, 160.32, 160.25, 154.62, 147.69, 130.35, 130.28, 119.29, 110.45, 110.44 107.81, 107.67, 107.40, 102.42, 102.26, 66.41, 37.52, 29.07. HRMS *m*/*z* calculated for C_15_H_14_FNO_4_ [M + H]^+^: 291.0907; found: 292.0992. > 99% purity (as determined by RP-HPLC, *t*_R_ = 6.89 min).

#### 4.4.14 *N*-(3-(4-Fluorophenoxy)­propyl)-2-oxo-2H-pyran-3-carboxamide (**6n**)

Compound **6n** (17.0 mg, 0.06 mmol) was prepared as a white solid in 8.0% yield by following the method described in general procedure C with **3n** (180.9 mg, 1.07 mmol). *R*_f_ = 0.60 (Hexane/EtOAc = 4/6, v/v). ^1^H NMR (600 MHz, CDCl_3_) δ 8.93 (s, 1H), 8.52 (dd, *J* = 6.8, 2.3 Hz, 1H), 7.68 (dd, *J* = 5.0, 2.3 Hz, 1H), 6.97 (t, *J* = 8.6 Hz, 2H), 6.88 (dd, *J* = 9.2, 4.3 Hz, 2H), 6.51 (dd, *J* = 6.9, 5.0 Hz, 1H), 4.03 (t, *J* = 5.9 Hz, 2H), 3.65–3.60 (m, 2H), 2.09 (p, *J* = 6.2 Hz, 2H). ^13^C NMR (150 MHz, CDCl_3_) δ 162.23, 161.73, 158.24, 156.66, 155.05, 154.60, 147.68, 119.32, 115.99, 115.84, 115.62, 115.57, 107.40, 66.81, 37.62, 29.18. HRMS *m*/*z* calculated for C_15_H_14_FNO_4_ [M + H]^+^: 291.0907; found: 292.0992. > 99% purity (as determined by RP-HPLC, *t*_R_ = 7.09 min).

#### 4.4.15 *N*-(3-(3,4-Dichlorophenoxy)­propyl)-2-oxo-2H-pyran-3-carboxamide (**6o**)

Compound **6o** (24.9 mg, 0.07 mmol) was prepared as a white solid in 10.1% yield by following the method described in general procedure C with **3o** (235.4 mg, 1.07 mmol). *R*_f_ = 0.50 (Hexane/EtOAc = 4/6, v/v). ^1^H NMR (600 MHz, CDCl_3_) δ 8.93 (s, 1H), 8.54 (d, *J* = 6.9 Hz, 1H), 7.72–7.66 (m, 1H), 7.34 (dd, *J* = 8.8, 1.3 Hz, 1H), 7.05 (dd, *J* = 3.0, 1.1 Hz, 1H), 6.84 (ddd, *J* = 8.8, 3.0, 1.1 Hz, 1H), 6.54 (ddd, *J* = 6.0, 4.5, 1.3 Hz, 1H), 4.09–4.02 (m, 2H), 3.66 (q, *J* = 6.2 Hz, 2H), 2.13 (q, *J* = 6.1 Hz, 2H). ^13^C NMR (150 MHz, CDCl_3_): δ162.36, 161.87, 158.08, 154.74, 147.83, 133.05, 130.92, 124.30 119.36, 116.48, 114.96, 107.52, 66.88, 37.51, 29.13. HRMS *m*/*z* calculated for C_15_H_13_Cl_2_NO_4_ [M + H]^+^: 341.0222; found: 342.0279. > 99% purity (as determined by RP-HPLC, *t*_R_ = 6.29 min).

#### 4.4.16 *N*-(3-(3,4-Difluorophenoxy)­propyl)-2-oxo-2H-pyran-3-carboxamide (**6p**)

Compound **6p** (28.5 mg, 0.09 mmol) was prepared as a white solid in 12.6% yield by following the method described in general procedure C with **3p** (200.1 mg, 1.07 mmol). *R*_f_ = 0.50 (Hexane/EtOAc = 6/4, v/v). ^1^H NMR (600 MHz, CDCl_3_) δ 8.92 (s, 1H), 8.52 (dd, *J* = 6.9, 2.3 Hz, 1H), 7.68 (dd, *J* = 5.0, 2.3 Hz, 1H), 7.05 (dt, *J* = 10.2, 9.1 Hz, 1H), 6.77 (ddd, *J* = 12.0, 6.6, 3.0 Hz, 1H), 6.66–6.63 (m, 1H), 6.52 (dd, *J* = 6.9, 5.0 Hz, 1H), 4.01 (t, *J* = 5.9 Hz, 2H), 3.66–3.60 (m, 2H), 2.09 (p, *J* = 6.2 Hz, 2H). ^13^C NMR (150 MHz, CDCl_3_) δ 162.11, 161.61, 155.13, 155.08, 154.51, 151.32, 151.22, 149.68, 149.58, 147.58, 145.91, 145.83, 144.32, 144.24, 119.14, 117.24, 117.11, 109.81, 109.79, 109.77, 109.75, 107.28, 104.17, 104.04, 66.87, 37.36, 28.92. HRMS *m*/*z* calculated for C_15_H_13_F_2_NO_4_ [M + H]^+^: 309.8013; found: 310.0874. > 99% purity (as determined by RP-HPLC, *t*_R_ = 4.48 min).

#### 4.4.17 *N*-(3-(3-Chloro-4-(trifluoromethyl)­phenoxy)­propyl)-2-oxo-2H-pyran-3-carboxamide (**6q**)

Compound **6q** (21.3 mg, 0.06 mmol) was prepared as a white solid in 7.9% yield by following the method described in general procedure C with **3q** (271.2 mg, 1.07 mmol). *R*_f_ = 0.50 (Hexane/EtOAc = 6/4, v/v). ^1^H NMR (600 MHz, CDCl_3_) δ 8.92 (s, 1H), 8.52 (dd, *J* = 6.9, 2.3 Hz, 1H), 7.68 (dd, *J* = 5.0, 2.2 Hz, 1H), 7.38 (d, *J* = 8.7 Hz, 1H), 7.24 (d, *J* = 3.0 Hz, 1H), 7.04 (dd, *J* = 8.7, 3.0 Hz, 1H), 6.52 (dd, *J* = 6.9, 5.0 Hz, 1H), 4.08 (t, *J* = 5.9 Hz, 2H), 3.64 (q, *J* = 6.3 Hz, 2H), 2.15–2.09 (m, 2H). ^13^C NMR (151 MHz, CDCl_3_) δ 162.14, 161.66, 157.22, 154.54, 147.63, 132.32, 129.14, 128.93, 123.55, 123.36, 121.74, 119.10, 118.77, 113.92, 113.89, 113.85, 113.81, 107.29, 66.73, 37.26, 28.88. 13C NMR (150 MHz, CDCl3) δ 162.14, 161.66, 157.22, 154.54, 147.63, 132.32, 129.14, 128.93, 123.55, 123.36, 121.74, 119.10, 118.77, 113.92, 113.89, 113.85, 113.81, 107.29, 66.73, 37.26, 28.88. HRMS *m*/*z* calculated for C_16_H_13_ClF_3_NO_4_ [M + H]^+^: 375.0485; found: 376.0540. > 99% purity (as determined by RP-HPLC, *t*_R_ = 6.72 min).

#### 4.4.18 *N*-(3-(3-Nitro-4-(trifluoromethyl)­phenoxy)­propyl)-2-oxo-2H-pyran-3-carboxamide (**6r**)

Compound **6r** (20.0 mg, 0.02 mmol) was prepared as a white solid in 7.5% yield by following the method described in general procedure C with **3r** (282.6 mg, 1.07 mmol). *R*_f_ = 0.58 (Hexane/EtOAc = 4/6, v/v). ^1^H NMR (600 MHz, CDCl_3_) δ 8.94 (s, 1H), 8.52 (dd, *J* = 6.9, 2.2 Hz, 1H), 8.01 (d, *J* = 9.0 Hz, 1H), 7.69 (dd, *J* = 5.0, 2.2 Hz, 1H), 7.35 (d, *J* = 2.8 Hz, 1H), 7.16 (dd, *J* = 9.0, 2.8 Hz, 1H), 6.53 (dd, *J* = 6.9, 5.0 Hz, 1H), 4.19 (d, *J* = 5.9 Hz, 2H), 3.66 (q, *J* = 6.3 Hz, 2H), 2.17 (dd, *J* = 6.9, 5.8 Hz, 2H). ^13^C NMR (150 MHz, CDCl_3_) δ 162.31, 161.97,161.89, 154.79, 147.89, 141.11, 128.27, 128.25, 126.62, 126.39, 126.16, 124.66, 122.85, 121.03, 119.22, 119.10, 116.92, 114.95, 114.91, 114.87, 114.83, 107.47, 67.53, 37.19, 28.92. HRMS *m*/*z* calculated for C_16_H_13_F_3_N_2_O_6_ [M + H]^+^: 386.0726; found: 387.0772. > 99% purity (as determined by RP-HPLC, *t*_R_ = 5.256 min).

#### 4.4.19 *N*-(3-(3,5-Difluorophenoxy)­propyl)-2-oxo-2H-pyran-3-carboxamide (**6s**)

Compound **6s** (48.5 mg, 0.15 mmol) was prepared as a white solid in 21.9% yield by following the method described in general procedure C with **3s** (274 mg, 0.75 mmol). *R*_f_ = 0.50 (Hexane/EtOAc = 4/6, v/v). ^1^H NMR (600 MHz, CDCl_3_) δ 8.90 (s, 1H), 8.52 (dd, *J* = 6.9, 2.3 Hz, 1H), 7.68 (dd, *J* = 5.0, 2.3 Hz, 1H), 6.52 (dd, *J* = 6.9, 5.0 Hz, 1H), 6.47 (dd, *J* = 9.0, 2.2 Hz, 2H), 6.40 (tt, *J* = 9.0, 2.2 Hz, 1H), 4.02 (t, *J* = 5.9 Hz, 2H), 3.63 (q, *J* = 6.4 Hz, 2H), 2.10 (p, *J* = 6.2 Hz, 2H). ^13^C NMR (150 MHz, CDCl_3_) δ 164.65, 164.55, 163.02, 162.92, 162.25, 161.79, 160.93, 160.84, 160.74, 154.67, 147.73, 119.24, 107.41, 98.56, 98.51, 98.40, 98.36, 96.73, 96.56, 96.39, 66.79, 37.38, 28.95. HRMS *m*/*z* calculated for C_15_H_13_F_2_NO_4_ [M + H]^+^: 309.0813; found: 310.0865. > 99% purity (as determined by RP-HPLC, *t*_R_ = 4.07 min).

#### 4.4.20 *N*-(3-(2,4-Difluorophenoxy)­propyl)-2-oxo-2H-pyran-3-carboxamide (**6t**)

Compound **6t** (28.5 mg, 0.02 mmol) was prepared as a white solid in 10.9% yield by following the method described in general procedure C with **3t** (274 mg, 0.75 mmol). *R*_f_ = 0.50 (Hexane/EtOAc = 4/6, v/v). ^1^H NMR (600 MHz, CDCl_3_) δ 8.87 (s, 1H), 8.54 (dd, *J* = 6.9, 2.2 Hz, 1H), 7.70 (dd, *J* = 5.0, 2.3 Hz, 1H), 6.96 (td, *J* = 9.2, 5.3 Hz, 1H), 6.87 (ddd, *J* = 11.2, 8.4, 3.0 Hz, 1H), 6.80 (td, *J* = 8.1, 2.3 Hz, 1H), 6.54 (dd, *J* = 6.9, 5.0 Hz, 1H), 4.11 (d, *J* = 6.0 Hz, 2H), 3.67 (q, *J* = 6.5 Hz, 2H), 2.14 (p, *J* = 6.4 Hz, 2H). ^13^C NMR (150 MHz, CDCl_3_) δ 162.27, 161.86, 157.66, 155.99, 154.62, 153.78, 153.69, 152.12, 147.67, 143.52, 143.50, 143.46, 119.28, 116.49, 116.47, 116.42, 116.40, 110.60, 110.57, 110.45, 110.43, 107.40, 105.20, 105.06, 105.03, 104.88, 68.31, 37.02, 29.36. HRMS *m*/*z* calculated for C_15_H_13_F_2_NO_4_ [M + H]^+^: 309.0813; found: 310.0857. > 99% purity (as determined by RP-HPLC, *t*_R_ = 4.68 min).

### 4.5 Strain, Reagents, and Cell Growth

The *C. albicans* DAY185 (fluconazole-resistant) strain was obtained from the Korean Culture Centre for Microorganisms (KCCM). The strain was cultured on solid potato dextrose agar (PDA) plates by streaking from a glycerol stock (15% glycerol) stored at −80 °C to promote colony growth. For subsequent experiments, liquid potato dextrose broth (PDB) was used. After a 2-day incubation of the PDA plates at 37 °C, a single colony was selected and inoculated into 25 mL of PDB in 250 mL Erlenmeyer flasks, where it was cultured for 48 h at 37 °C. Dimethyl sulfoxide (DMSO) and crystal violet were obtained from Sigma-Aldrich (St. Louis, USA), and the synthesized compounds were dissolved in DMSO. The DMSO concentration in the cell cultures was maintained below 0.1% (v/v), ensuring no impact on the growth or biofilm formation of *C. albicans* . Planktonic cell growth was assessed by measuring absorbance at 620 nm using Multiskan SkyHigh microplate reader (Thermo Fisher Scientific, Waltham, USA) after 24 h of incubation at 37 °C. Two independent experiments were conducted, each with six replicates at every concentration.

### 4.6 Biofilm Assays

*C. albicans* biofilms were formed on 96-well plates as described previously.^45^ Overnight cultures of *C. albicans* cells were subinoculated into fresh PDB (300 μL) at an optical density of 0.1 at 600 nm in 96-well plates. The cells were incubated with or without synthesized compounds for 24 h at 37 °C without agitation under dark condition. After incubation, planktonic cells and spent medium were removed, and the plates were washed three times with water to remove nonbiofilm cells. The biofilm cells on the well surfaces were stained with 0.1% (w/v) crystal violet (Sigma-Aldrich) for 20 min, followed by three washes with water. The crystal violet was then solubilized using 95% ethanol, and the absorbance was measured at 570 nm using a spectrophotometer (Multiskan SkyHigh microplate reader; Thermo Fisher Scientific, Waltham, USA) after vigorous shaking for 90 s. Two independent experiments, each with six replicates per concentration, were conducted.

### 4.7 Microscopic Assays

*C. albicans* DAY185 biofilms were grown for 24 h at 37 °C following the previously described procedure. Afterward, nonbiofilm cells and spent medium were gently removed by washing the wells three times with water. The biofilm cells were then hydrated with 30 μL of PBS and visualized using live cell imaging microscopy with the iRiS Digital Cell Imaging System (Logos BioSystems, Anyang, Republic of Korea). Color-coded 2D and 3D images were generated using ImageJ (https://imagej.nih.gov/ij).^45^ Four independent experiments with six replicates per concentration were performed.

### 4.8 Molecular Docking of the Derivatives with ALS3 Protein

The high-resolution structure of ALS3, resolved at 1.4 Å, is available under PDB ID 4LEB. This structure includes a hepta-threonine molecule as chain B. Ligands were prepared by converting their SMILES strings into 3D SDF files using the NovaPro web application (https://www.novoprolabs.com/tools/smiles2pdb; accessed January 23, 2024). Molecular docking was carried out with Calici’s Pharmaco-Net 2.0 (https://app.pharmaco-net.org/; accessed January 23, 2024). The 4LEB structure was uploaded to the Pharmaco-Net server, and ligand files served as inputs. Using the Pocket Finder module, chain B (hepta-threonine) was removed, and the protein was repaired to address missing residues and optimize energy. The docking grid and key residues in chain A, which corresponds to the N-terminal peptide binding cavity of ALS3 adhesin, were identified. A cubic docking grid of 155 units was generated, with its center at coordinates *X*: 1.8, *Y*: 5.0, and *Z*: −13.3. The presence of a binding pocket within the grid coordinates was confirmed using the castP server (https://sts.bioe.uic.edu/; accessed January 23, 2024). Docking simulations were performed with the AI-Dock Plus module, which evaluated the binding energies of ligands docked to the defined grid. The resulting docked poses and protein–ligand interactions were visualized using Discovery Studio Visualizer (Dassault Systèmes, France).

### 4.9 Statistics

The number of replicates for the assays is provided above, and the results are expressed as mean ± standard deviation. Statistical analysis was performed using one-way ANOVA, followed by Dunnett’s test, in SPSS version 23 (SPSS Inc., Chicago, USA). Statistical significance was defined as a *p*-value below 0.05, with asterisks indicating notable differences between treated and untreated samples.

## Supplementary Material





## Notes

Molecular formula strings and biological data for final compounds (CSV)
Analytical data (^1^H NMR and ^13^C NMR) on key intermediates and final compounds (PDF)

## Abbreviations

ALS1: agglutinin-like sequence 1
ALS3: agglutinin-like sequence 3
DMF: dimethylformamide
DMSO: dimethyl sulfoxide
DCM: dichloromethane
TLC: thin-layer chromatography
TEA: triethylamine
TFA: trifluoroacetic acid
PBS: phosphate-buffered saline
SAR: structure–activity relationship
