# Supplementary material for: Novel Pyrone-Based Biofilm Inhibitors against Azole-Resistant Candida albicans
Source: ACS Omega. 2025 Aug 11;10(32):36441–54. doi: 10.1021/acsomega.5c04793 (PMC12368727; doi:10.1021/acsomega.5c04793)

Supporting Information for

Novel Pyrone-based Biofilm Inhibitors Against Azole-resistant *Candida albicans*

Ji-eun Yang[1,#] Jin-Hyung Lee[2,#] Bharath Reddy Boya[2], Yong-Guy Kim[2], Youngjoo Byun[1,3,*] Jintae Lee[2,*]

[1]College of Pharmacy, Korea University, 2511 Sejong-ro, Sejong 30019, Republic of Korea

[2]School of Chemical Engineering, Yeungnam University, Gyeongsan, , Kyeongbuk 38541, Republic of Korea

[3]Interdisciplinary Major Program in Innovative Pharmaceutical Sciences, Korea University, Sejong 30019, Republic of Korea

[#] Equal contribution

*Corresponding authors

Youngjoo Byun, Ph.D.

College of Pharmacy, Korea University, 2511 Sejong-ro, Jochiwon-eup, Sejong 30019, Republic of Korea

Tel.: +82-44-860-1619; Fax: +82-44-860-1607; E-mail: yjbyun1@korea.ac.kr

Jintae Lee, Ph.D.

School of Chemical Engineering, Yeungnam University, Gyeongsan, 38541, Republic of Korea

Tel.: +82-53-810-2533; Fax: +82-53-810-4631; E-mail: jtlee@ynu.ac.kr

Table of contents

**Content**	**page**
^{1}H NMR (A) and ^{13}C NMR (B) spectra of compound **6a**	S3
^{1}H NMR (A) and ^{13}C NMR (B) spectra of compound **6b**	S4
^{1}H NMR (A) and ^{13}C NMR (B) spectra of compound **6c**	S5
^{1}H NMR (A) and ^{13}C NMR (B) spectra of compound **6d**	S6
^{1}H NMR (A) and ^{13}C NMR (B) spectra of compound **6e**	S7
^{1}H NMR (A) and ^{13}C NMR (B) spectra of compound **6f**	S8
^{1}H NMR (A) and ^{13}C NMR (B) spectra of compound **6g**	S9
^{1}H NMR (A) and ^{13}C NMR (B) spectra of compound **6h**	S10
^{1}H NMR (A) and ^{13}C NMR (B) spectra of compound **6i**	S11
^{1}H NMR (A) and $_{13}C$ NMR (B) spectra of compound **6j**	S12
^{1}H NMR (A) and ^{13}C NMR (B) spectra of compound **6k**	S13
^{1}H NMR (A) and ^{13}C NMR (B) spectra of compound **6l**	S14
^{1}H NMR (A) and ^{13}C NMR (B) spectra of compound **6m**	S15
^{1}H NMR (A) and ^{13}C NMR (B) spectra of compound **6n**	S16
^{1}H NMR (A) and ^{13}C NMR (B) spectra of compound **6o**	S17
^{1}H NMR (A) and ^{13}C NMR (B) spectra of compound **6p**	S18
^{1}H NMR (A) and ^{13}C NMR (B) spectra of compound **6q**	S19
^{1}H NMR (A) and ^{13}C NMR (B) spectra of compound **6r**	S20
^{1}H NMR (A) and ^{13}C NMR (B) spectra of compound **6s**	S21
^{1}H NMR (A) and ^{13}C NMR (B) spectra of compound **6t**	S22
HPLC trace of compound (A) **6a** (B) **6b** (C) **6c** (D) **6d**	S23
HPLC trace of compound (A) **6e** (B) **6f** (C) **6g** (D) **6h**	S24
HPLC trace of compound (A) **6i** (B) **6j** (C) **6k** (D) **6l**	S25
HPLC trace of compound (A) **6m** (B) **6n** (C) **6o** (D) **6p**	S26
HPLC trace of compound (A) **6q** (B) **6r** (C) **6s** (D) **6t**	S27

(A) ^{1}H NMR spectra of compound **6a** measured in $CDCl_3$ at 600 MHz

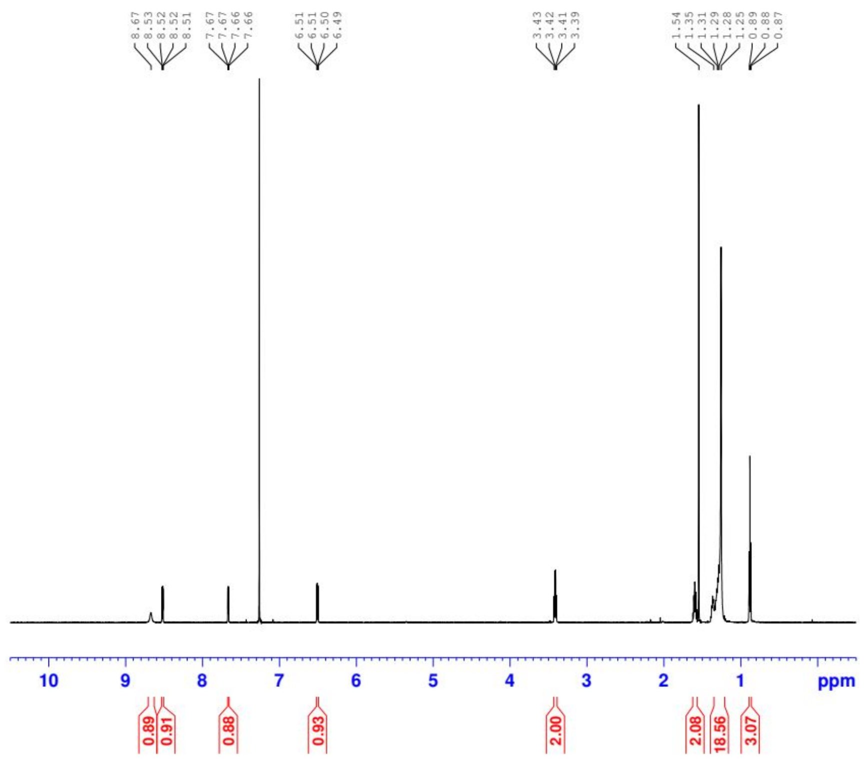

(B) ^{13}C NMR spectra of compound **6a** measured in $CDCl_3$ at 150 MHz

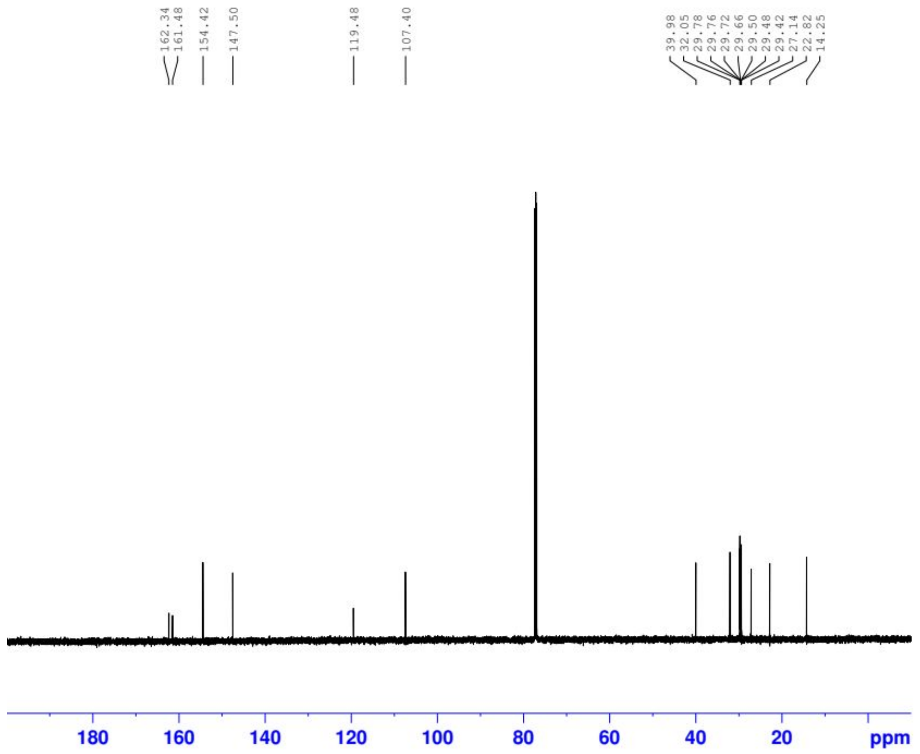

^{1}H NMR (A) and ^{13}C NMR (B) spectra of compound **6a**

(A) ^{1}H NMR spectra of compound **6b** measured in $CDCl_3$ at 600 MHz

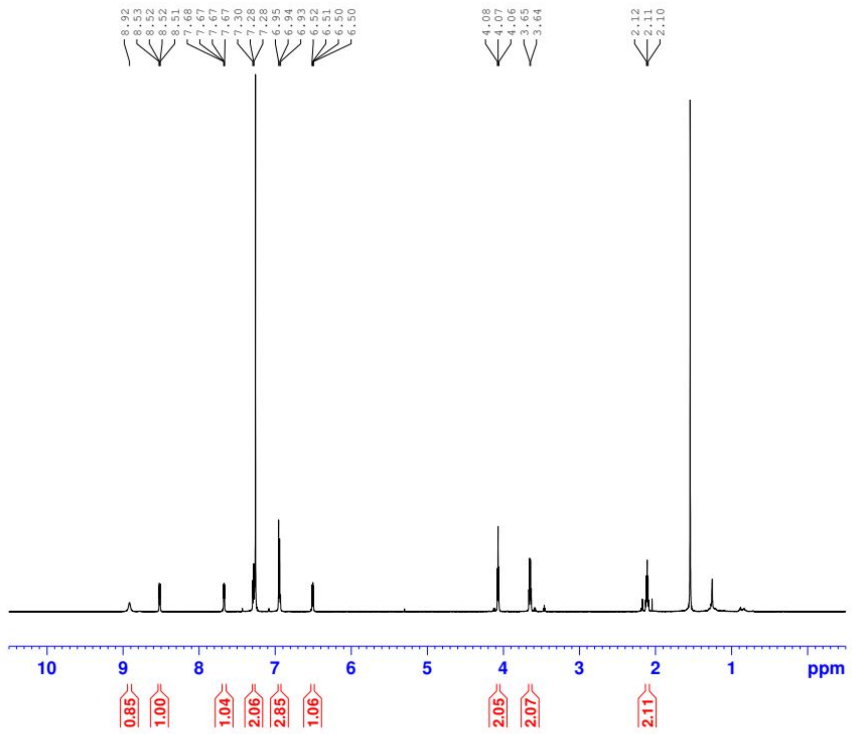

(B) ^{13}C NMR spectra of compound **6b** measured in $CDCl_3$ at 150 MHz

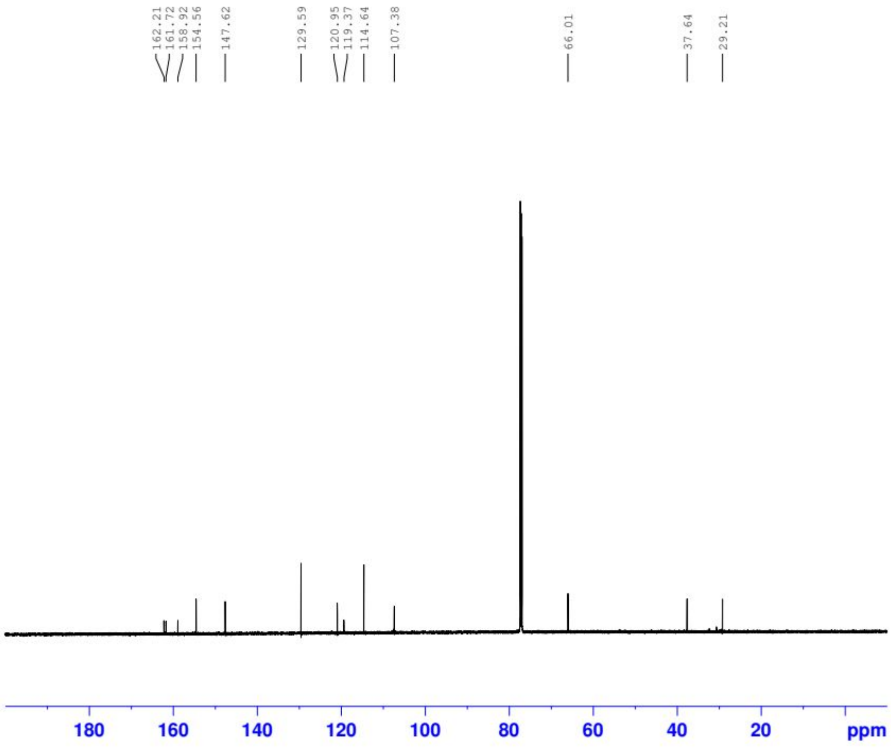

^{1}H NMR (A) and ^{13}C NMR (B) spectra of compound **6b**

(A) ^{1}H NMR spectra of compound **6c** measured in $CDCl_3$ at 600 MHz

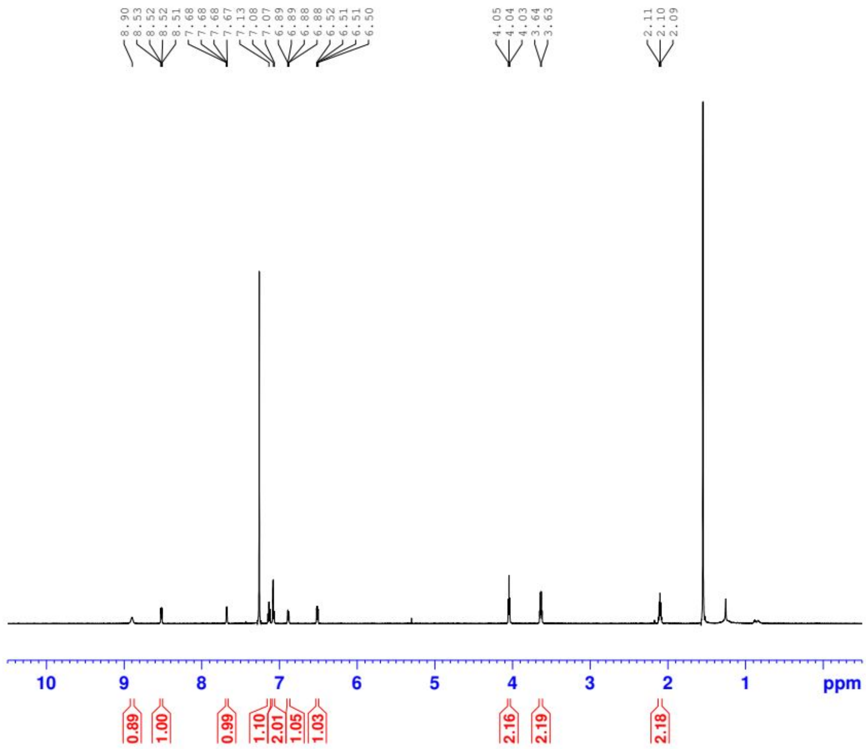

(B) ^{13}C NMR spectra of compound **6c** measured in $CDCl_3$ at 150 MHz

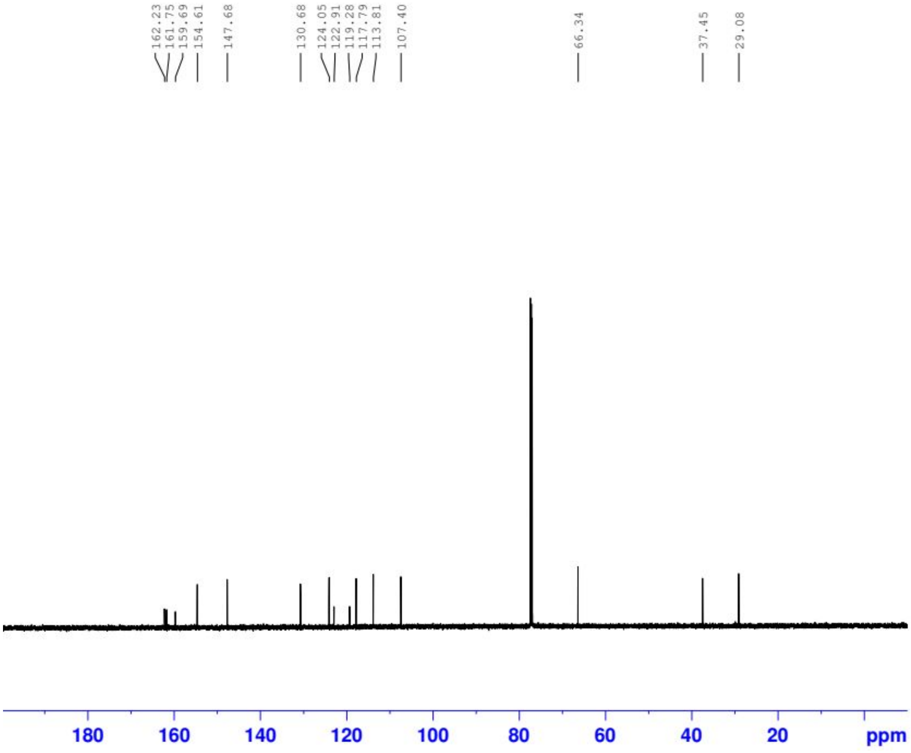

^{1}H NMR (A) and ^{13}C NMR (B) spectra of compound **6c**

^{1}H NMR spectra of compound **6c** measured in $CDCl_3$ at 600 MHzcontrol.

(A) ^1H NMR spectra of compound **6d** measured in $CDCl_3$ at 600 MHz

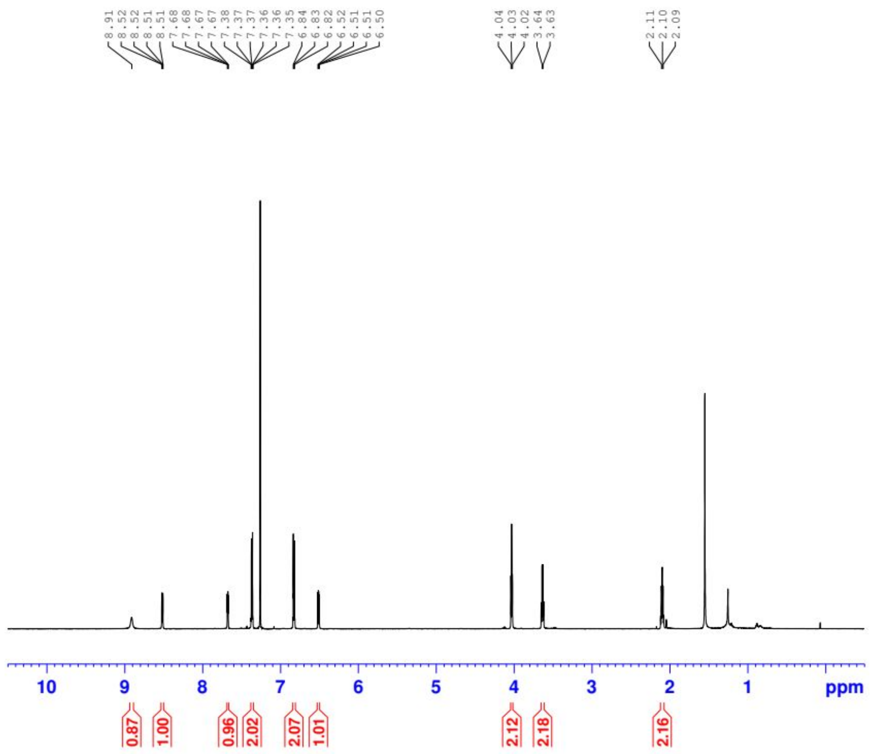

(B) ^{13}C NMR spectra of compound **6d**measured in $CDCl_3$ at 150 MHz

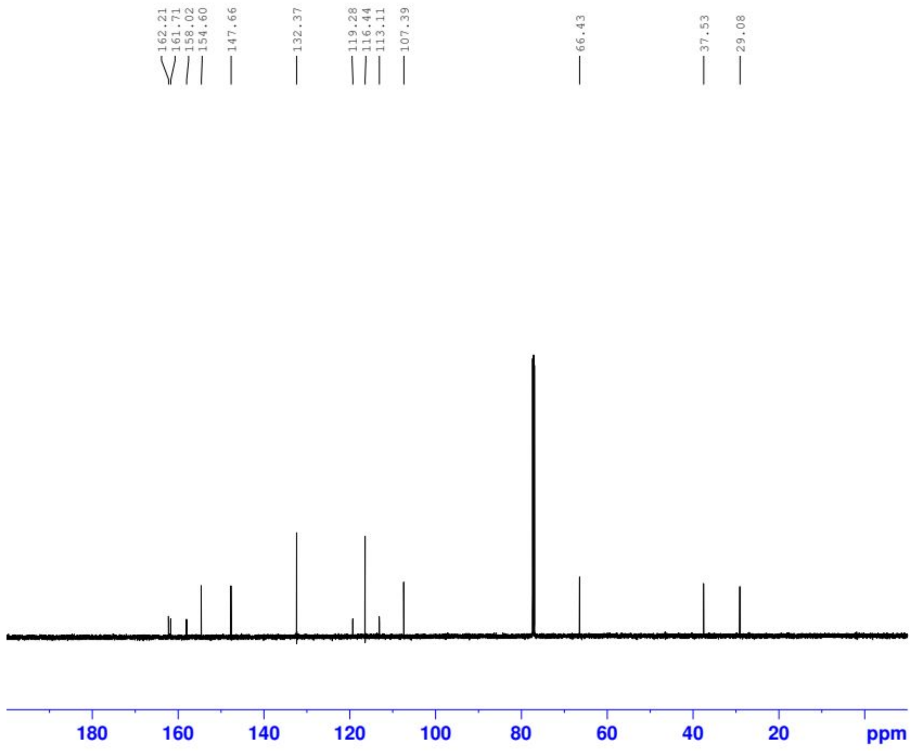

^1H NMR (A) and ^{13}C NMR (B) spectra of compound **6d**

^1H NMR spectra of compound **6d** measured in $CDCl_3$ at 600 MHzcontrol.

(A) ^{1}H NMR spectra of compound **6e** measured in $CDCl_3$ at 600 MHz

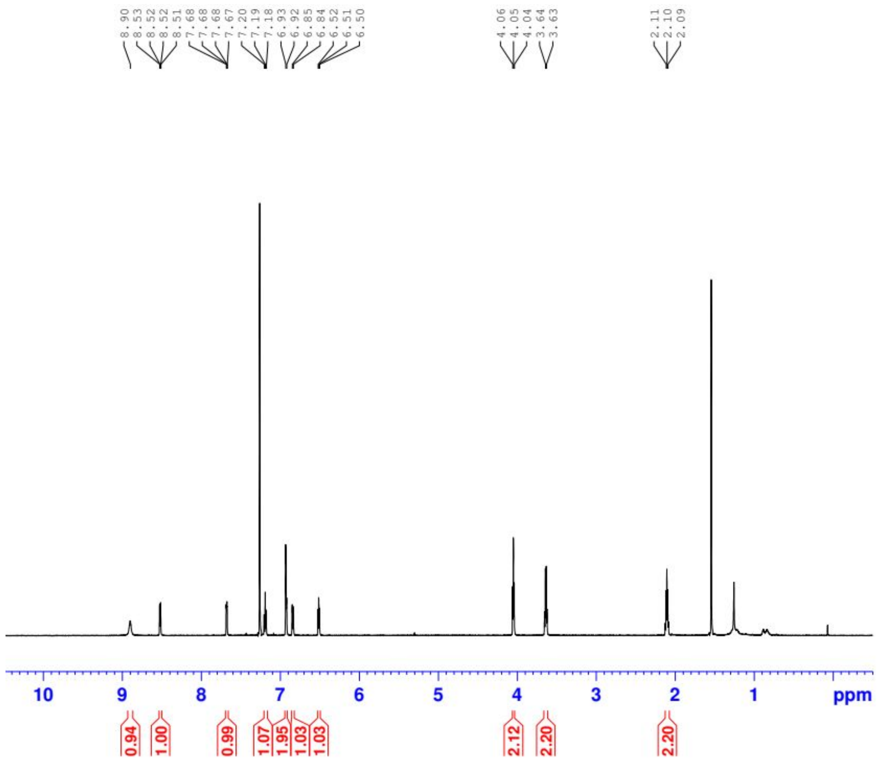

(B) ^{13}C NMR spectra of compound **6e** measured in $CDCl_3$ at 150 MHz

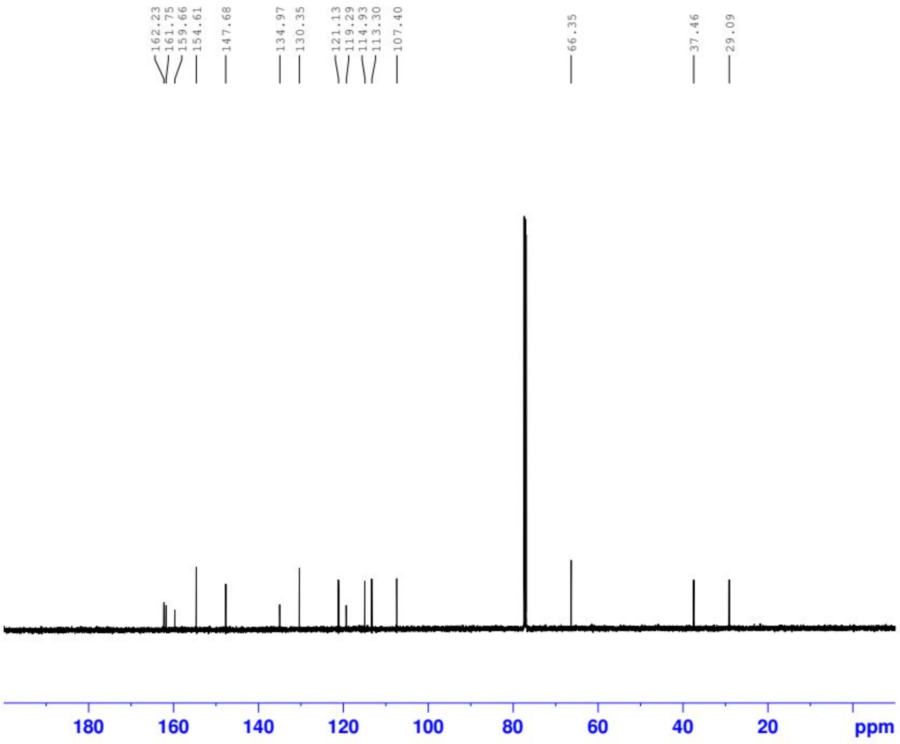

^{1}H NMR (A) and ^{13}C NMR (B) spectra of compound **6e**

^{1}H NMR spectra of compound **6e** measured in $CDCl_3$ at 600 MHzcontrol.

(A) ^{1}H NMR spectra of compound **6f** measured in $CDCl_3$ at 600 MHz

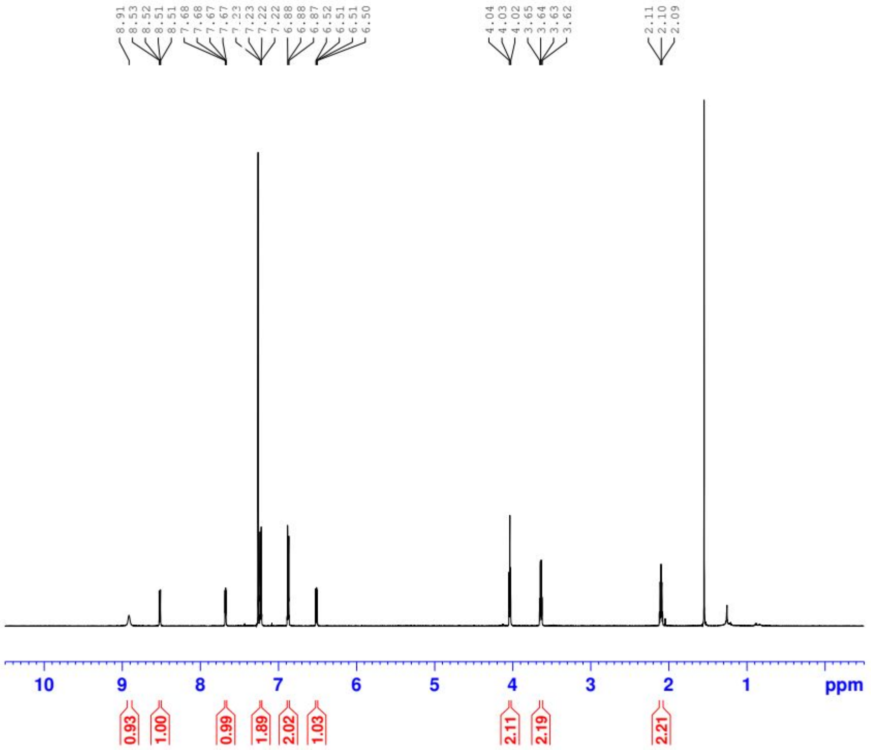

(B) ^{13}C NMR spectra of compound **6f** measured in $CDCl_3$ at 150 MHz

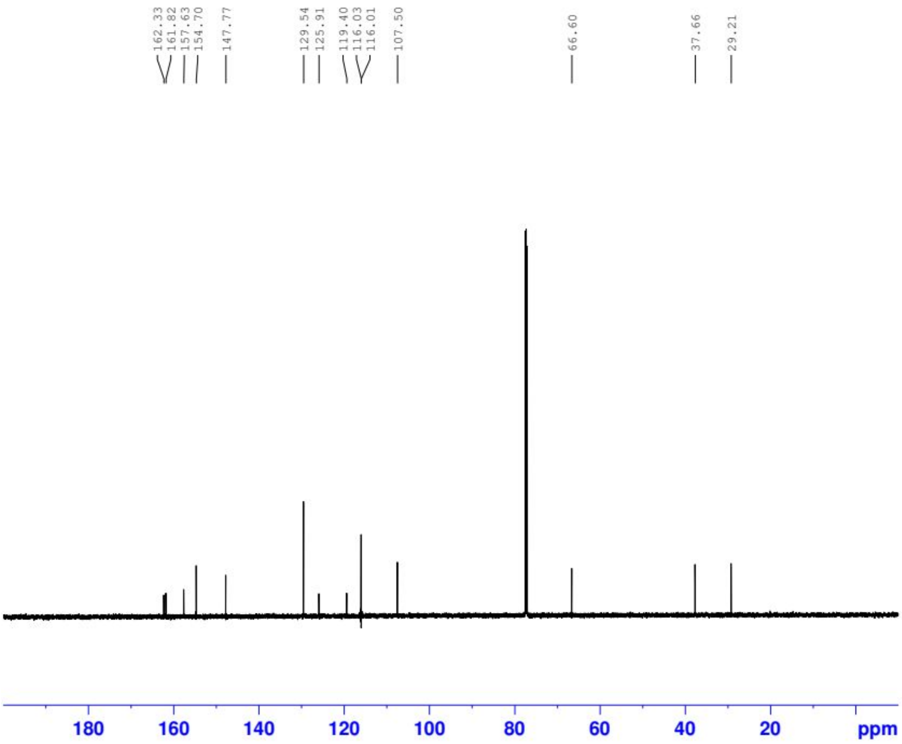

^{1}H NMR (A) and ^{13}C NMR (B) spectra of compound **6f**

^{1}H NMR spectra of compound **6f** measured in $CDCl_3$ at 600 MHzcontrol.

(A) ^{1}H NMR spectra of compound **6g** measured in $CDCl_3$ at 600 MHz

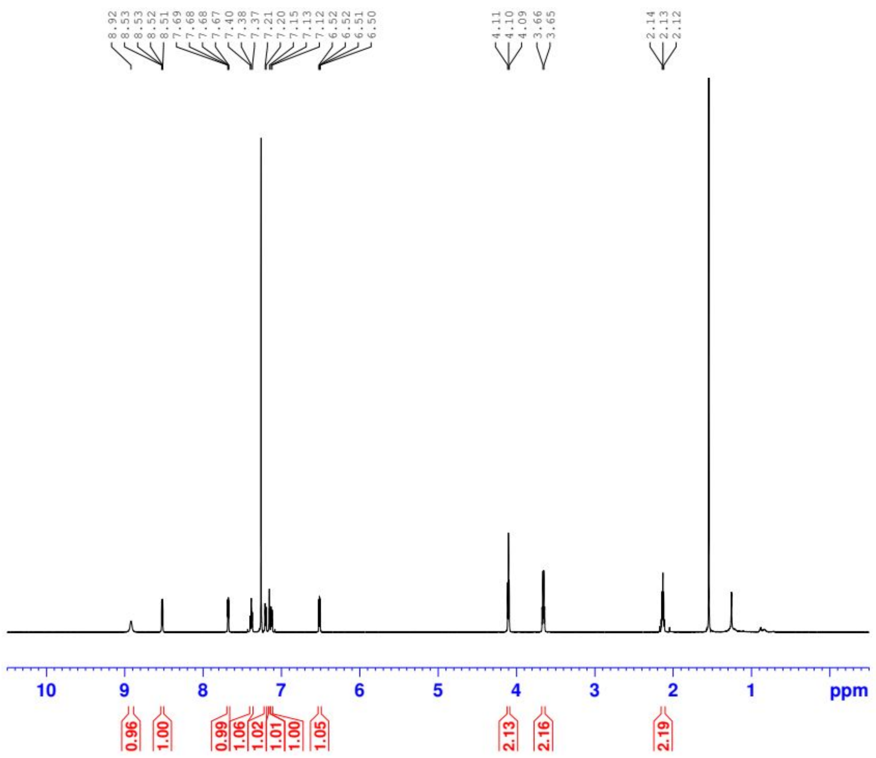

(B) ^{13}C NMR spectra of compound **6g** measured in $CDCl_3$ at 150 MHz

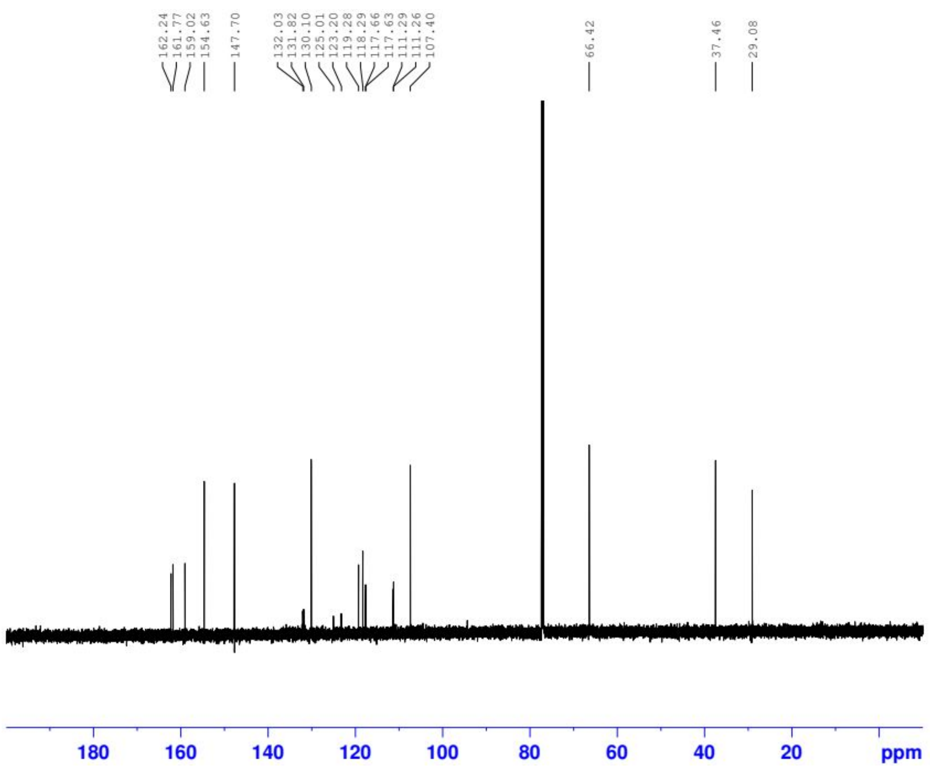

^{1}H NMR (A) and ^{13}C NMR (B) spectra of compound **6g**

^{1}H NMR spectra of compound **6g** measured in $CDCl_3$ at 600 MHzcontrol.

(A) ^{1}H NMR spectra of compound **6h** measured in $CDCl_3$ at 600 MHz

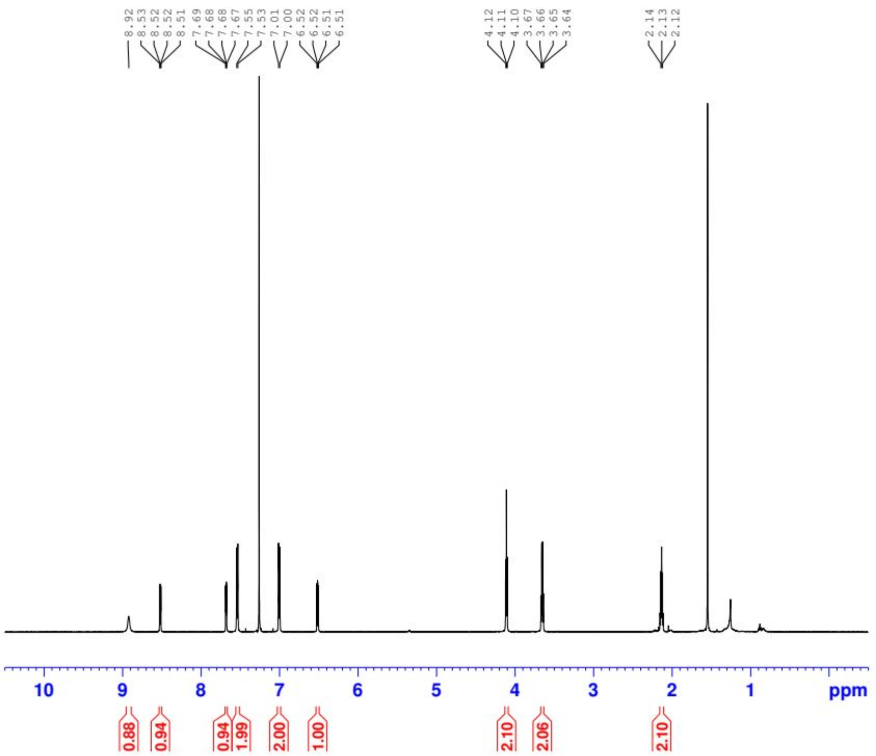

(B) ^{13}C NMR spectra of compound **6h** measured in $CDCl_3$ at 150 MHz

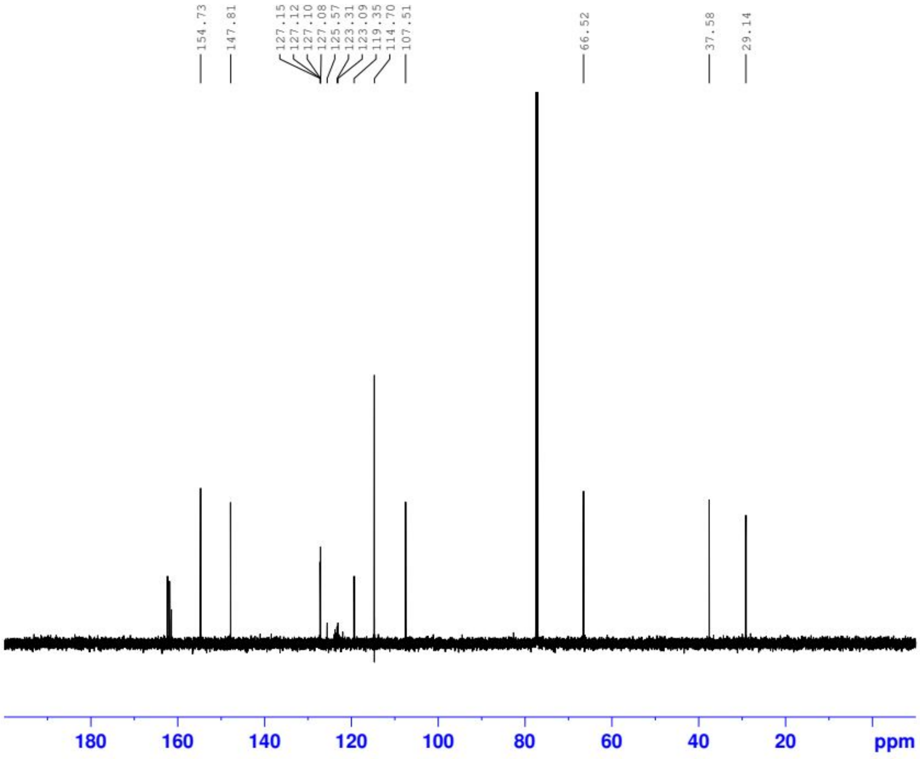

^{1}H NMR (A) and ^{13}C NMR (B) spectra of compound **6h**

^{1}H NMR spectra of compound **6h** measured in $CDCl_3$ at 600 MHzcontrol.

(A) ^{1}H NMR spectra of compound **6i** measured in $CDCl_3$ at 600 MHz

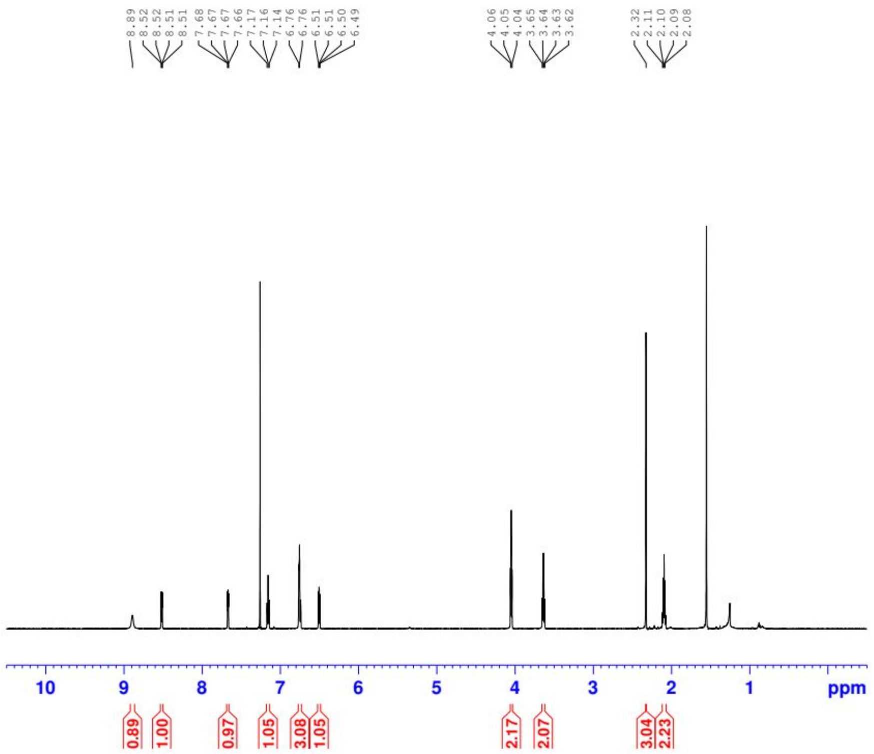

(B) ^{13}C NMR spectra of compound **6i** measured in $CDCl_3$ at 150 MHz

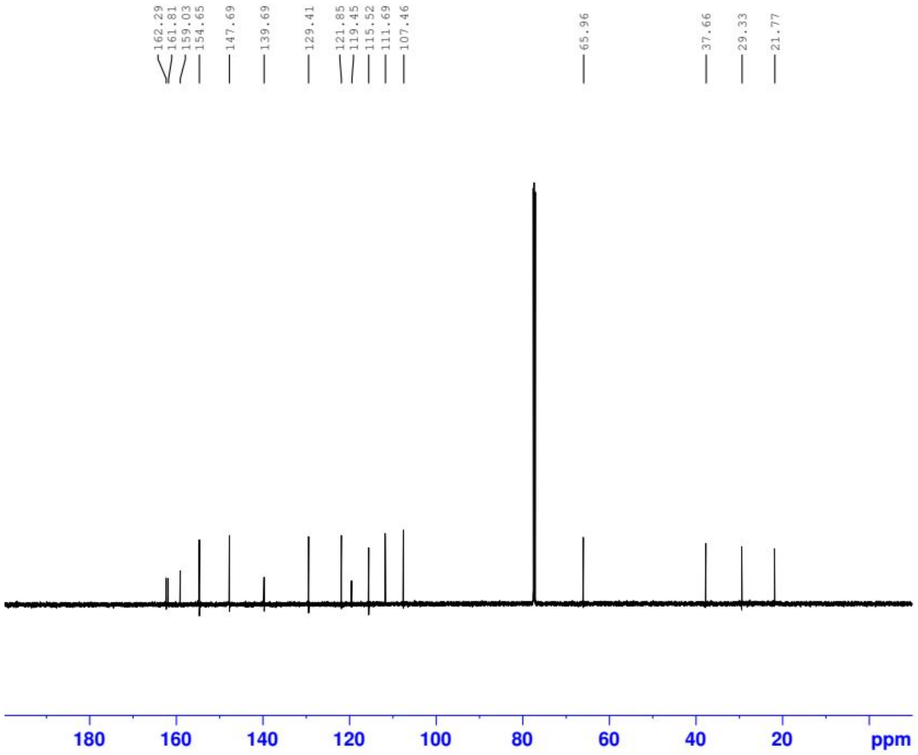

^{1}H NMR (A) and ^{13}C NMR (B) spectra of compound **6i**

^{1}H NMR spectra of compound **6i** measured in $CDCl_3$ at 600 MHzcontrol.

(A) ^{1}H NMR spectra of compound **6j** measured in $CDCl_3$ at 600 MHz

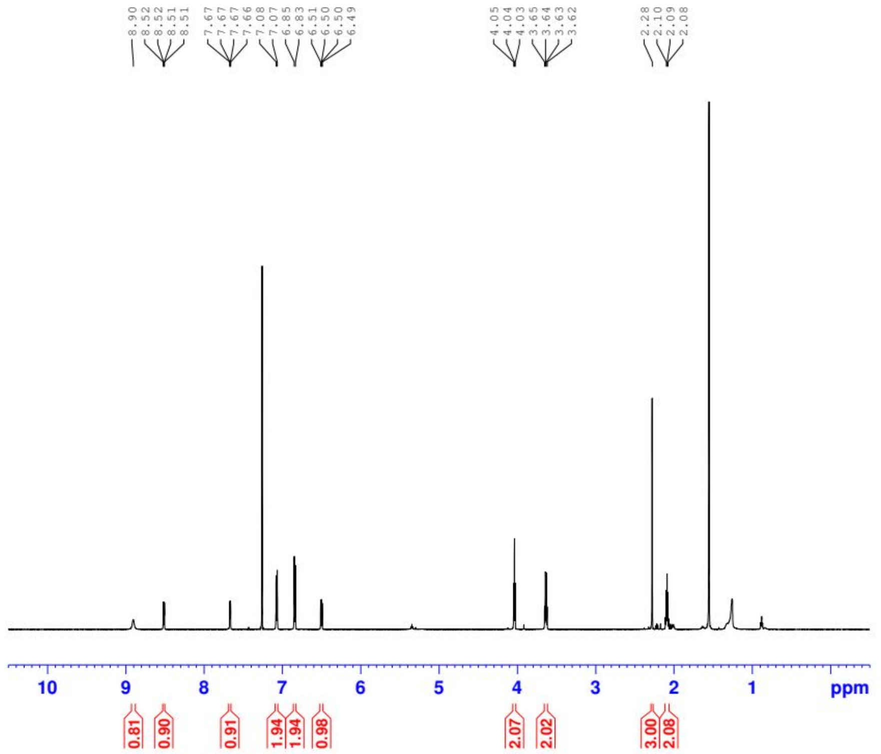

(B) ^{13}C NMR spectra of compound **6j** measured in $CDCl_3$ at 150 MHz

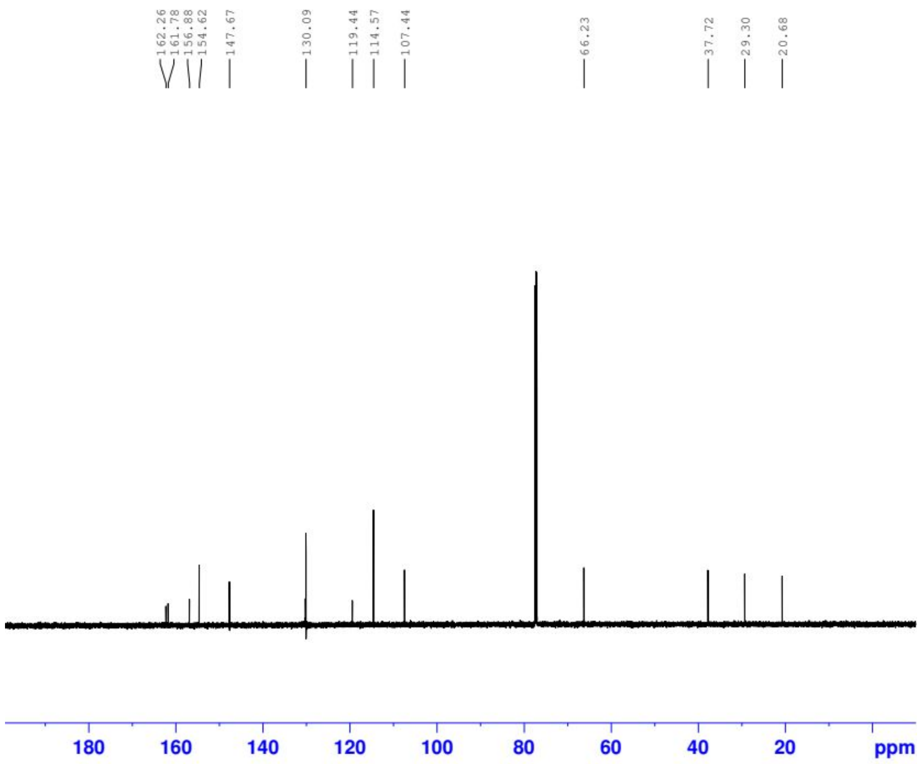

^{1}H NMR (A) and ^{13}C NMR (B) spectra of compound **6j**

^{1}H NMR spectra of compound **6j** measured in $CDCl_3$ at 600 MHzcontrol.

(A) ^{1}H NMR spectra of compound **6k** measured in $CDCl_3$ at 600 MHz

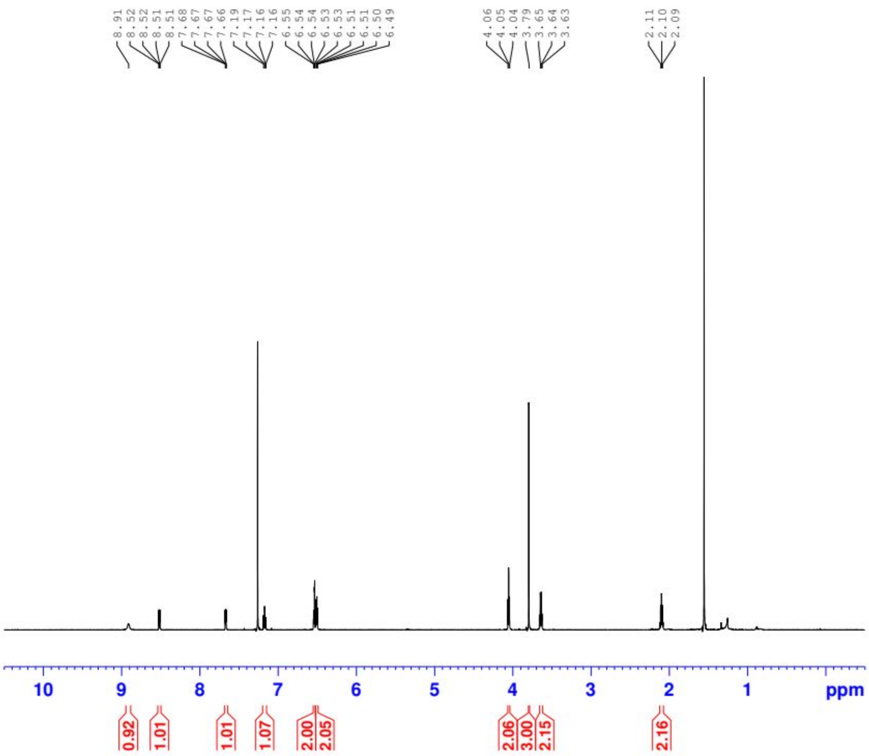

(B) ^{13}C NMR spectra of compound **6k** measured in $CDCl_3$ at 150 MHz

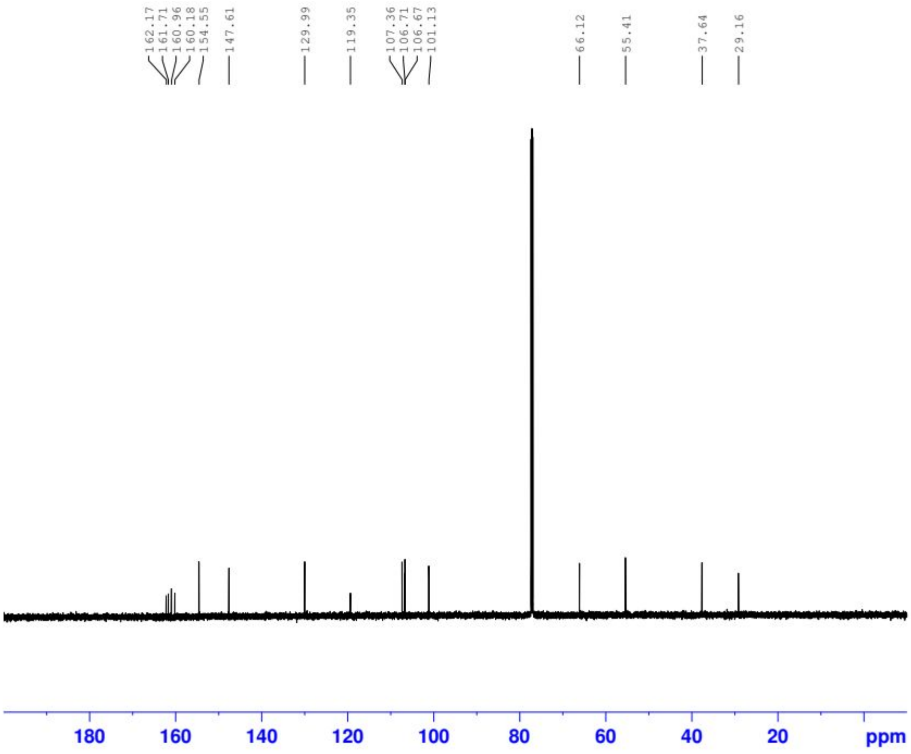

^{1}H NMR (A) and ^{13}C NMR (B) spectra of compound **6k**

^{1}H NMR spectra of compound **6k** measured in $CDCl_3$ at 600 MHzcontrol.

(A) ^{1}H NMR spectra of compound **6l** measured in $CDCl_3$ at 600 MHz

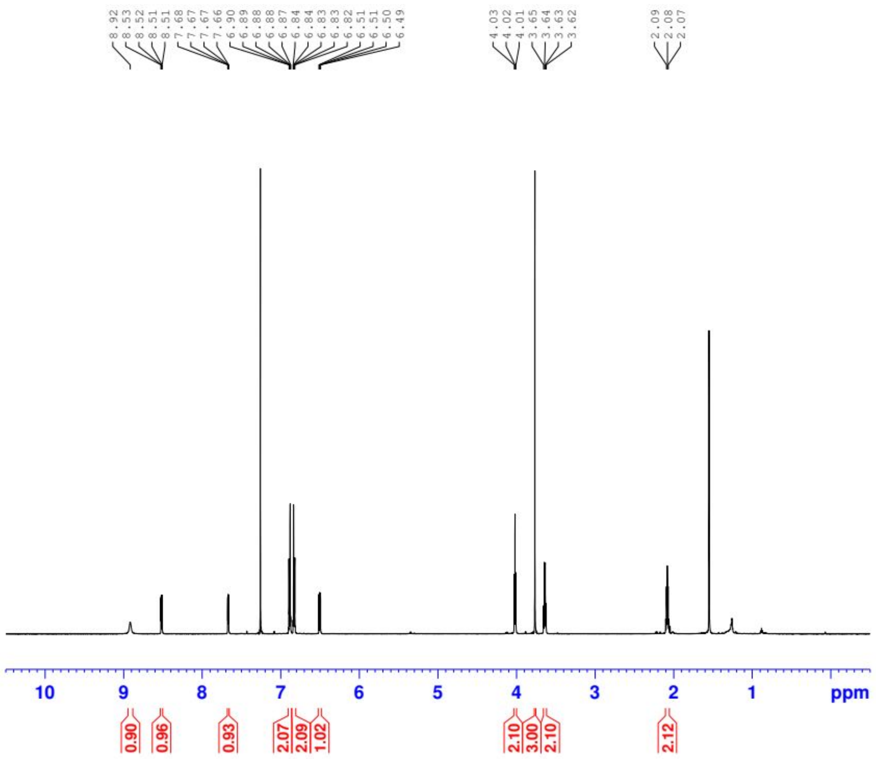

(B) ^{13}C NMR spectra of compound **6l** measured in $CDCl_3$ at 150 MHz

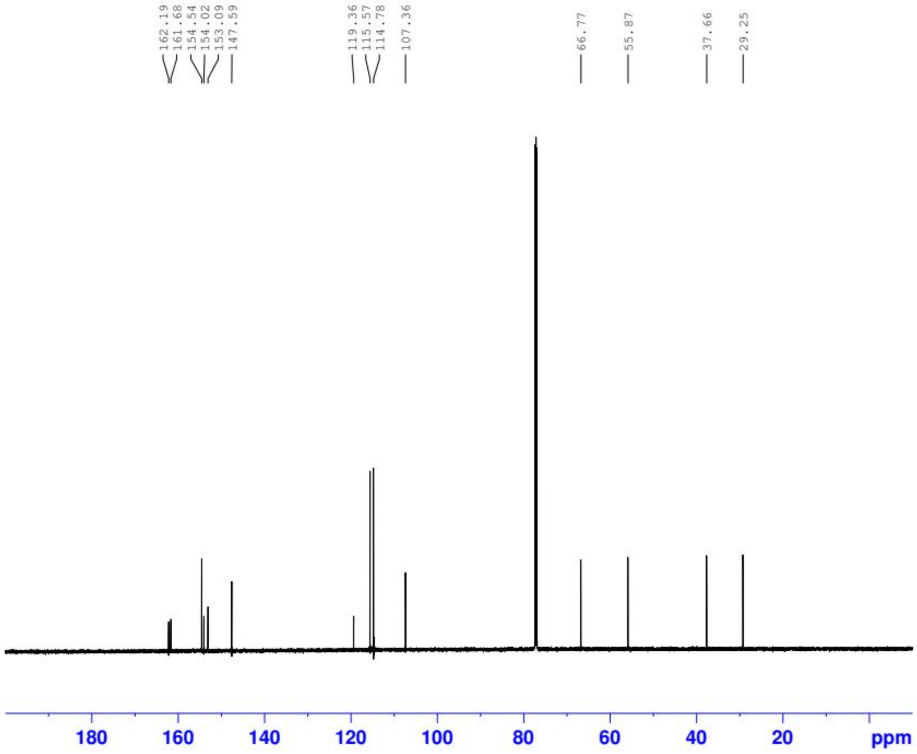

^{1}H NMR (A) and ^{13}C NMR (B) spectra of compound **6l**

^{1}H NMR spectra of compound **6l** measured in $CDCl_3$ at 600 MHzcontrol.

(A) ^{1}H NMR spectra of compound **6m** measured in $CDCl_3$ at 600 MHz

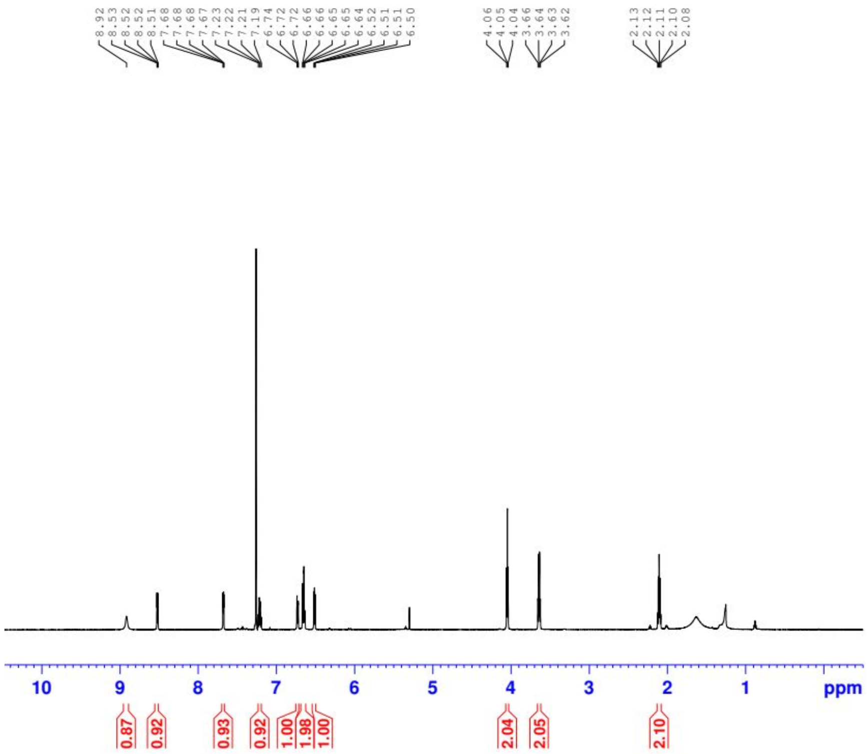

(B) ^{13}C NMR spectra of compound **6m** measured in $CDCl_3$ at 150 MHz

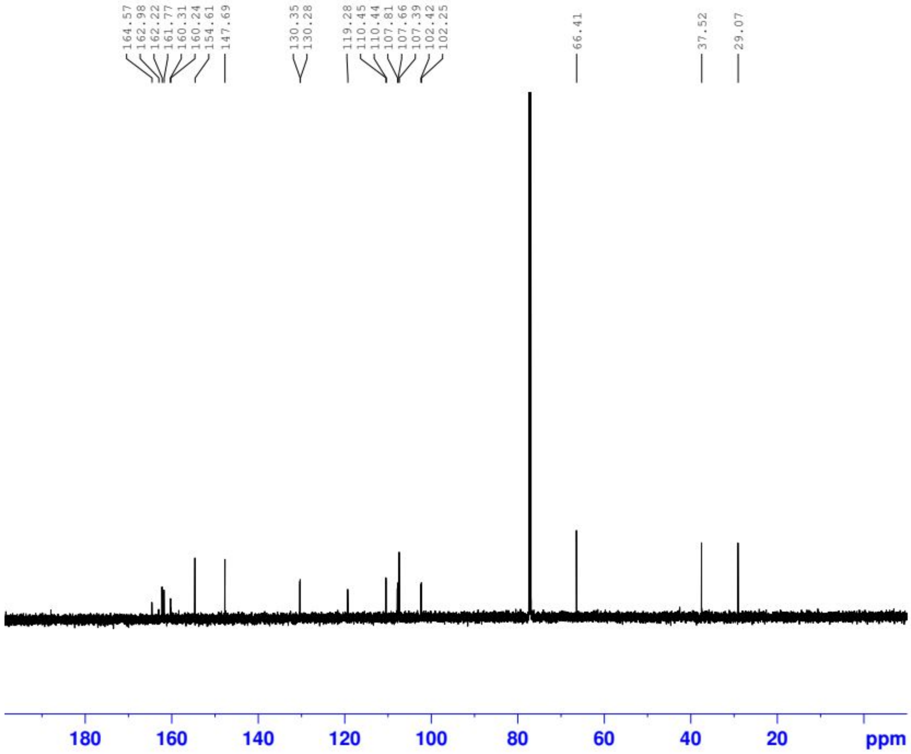

^{1}H NMR (A) and ^{13}C NMR (B) spectra of compound **6m**

^{1}H NMR spectra of compound **6m** measured in $CDCl_3$ at 600 MHzcontrol.

(A) ^{1}H NMR spectra of compound **6n** measured in $CDCl_3$ at 600 MHz

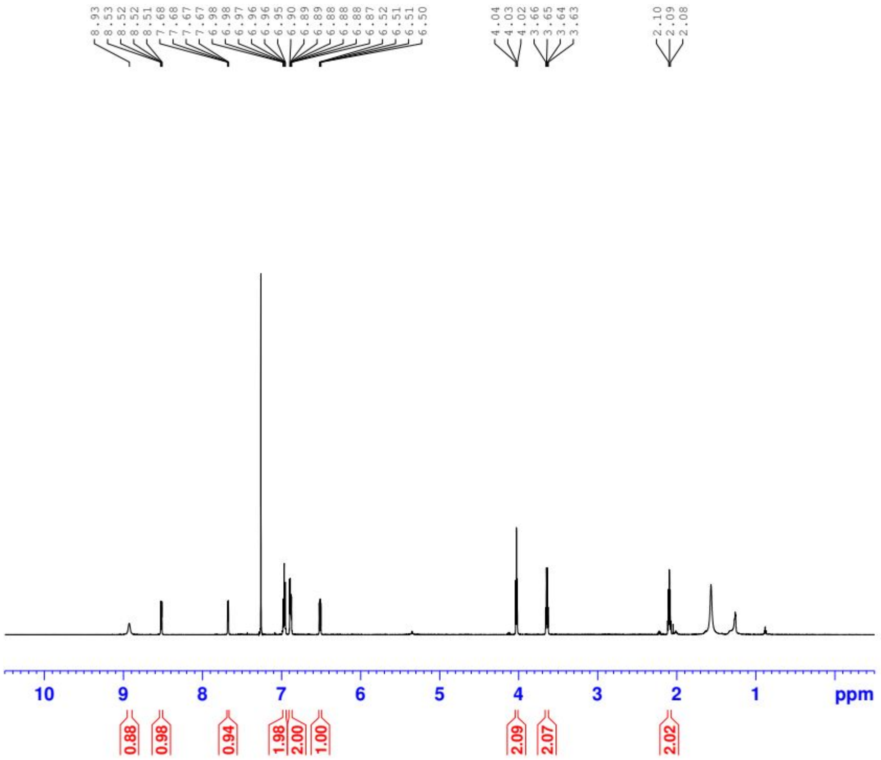

(B) ^{13}C NMR spectra of compound **6n** measured in $CDCl_3$ at 150 MHz

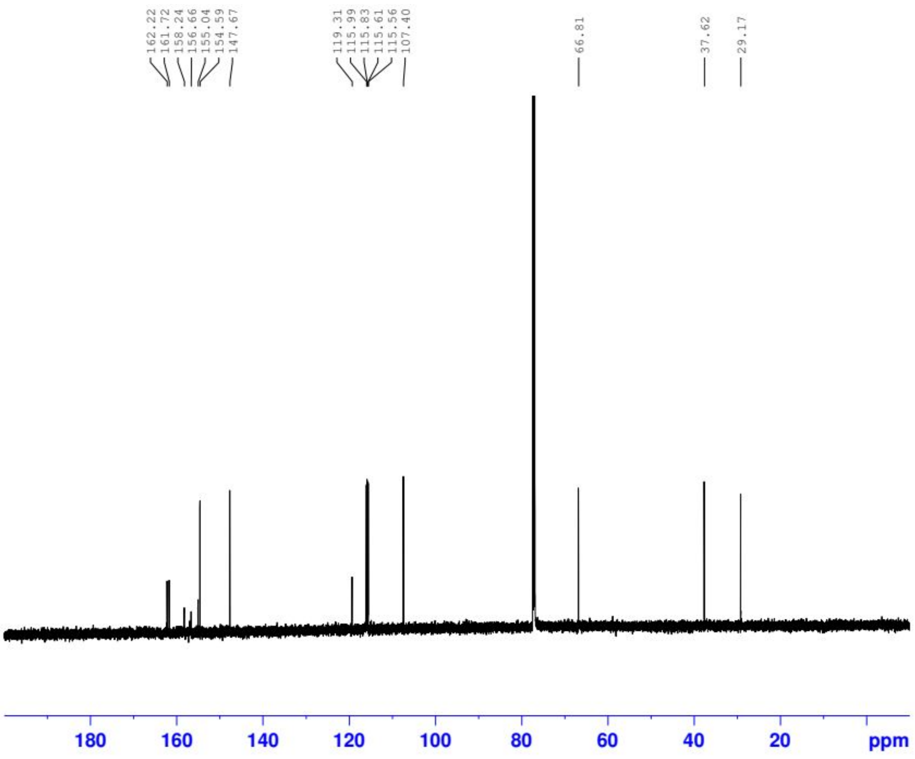

^{1}H NMR (A) and ^{13}C NMR (B) spectra of compound **6n**

^{1}H NMR spectra of compound **6n** measured in $CDCl_3$ at 600 MHzcontrol.

(A) ^{1}H NMR spectra of compound **6o** measured in $CDCl_3$ at 600 MHz

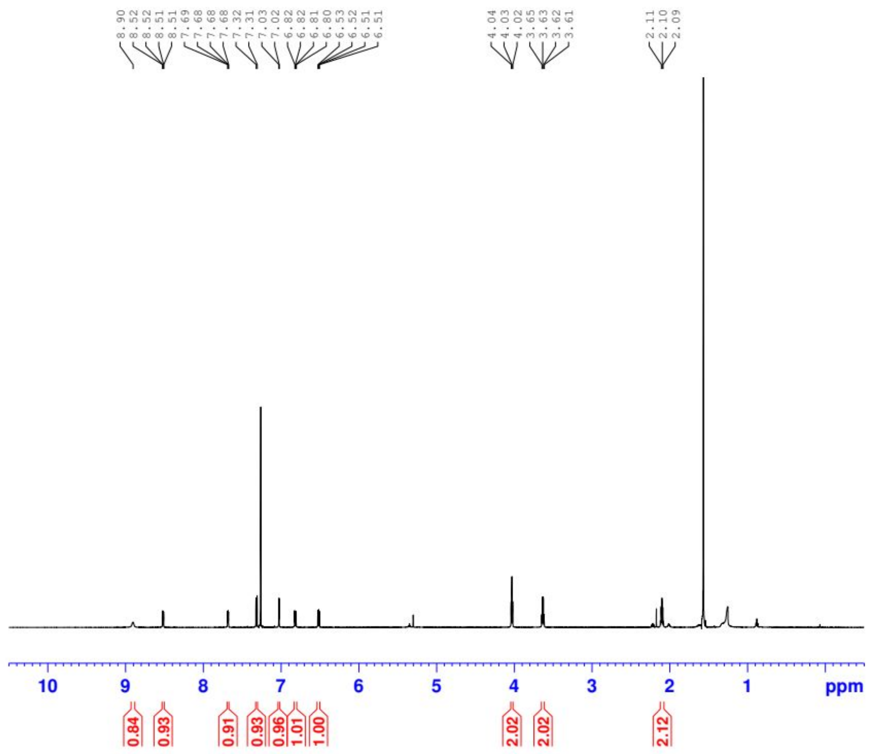

(B) ^{13}C NMR spectra of compound **6o** measured in $CDCl_3$ at 150 MHz

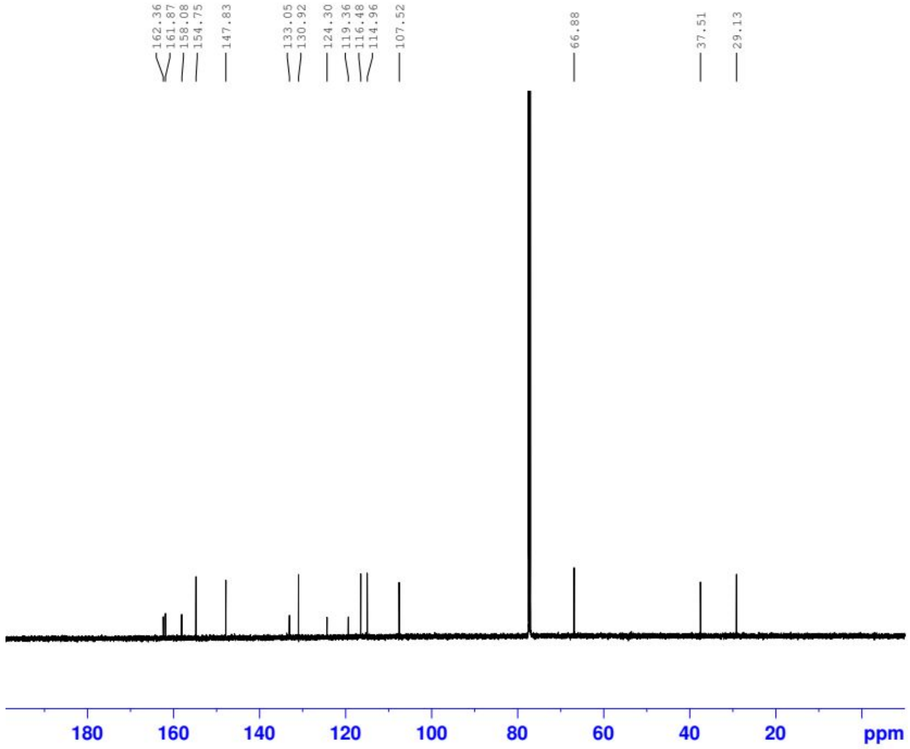

^{1}H NMR (A) and ^{13}C NMR (B) spectra of compound **6o**

^{1}H NMR spectra of compound **6o** measured in $CDCl_3$ at 600 MHzcontrol.

(A) ^{1}H NMR spectra of compound **6p** measured in $CDCl_3$ at 600 MHz

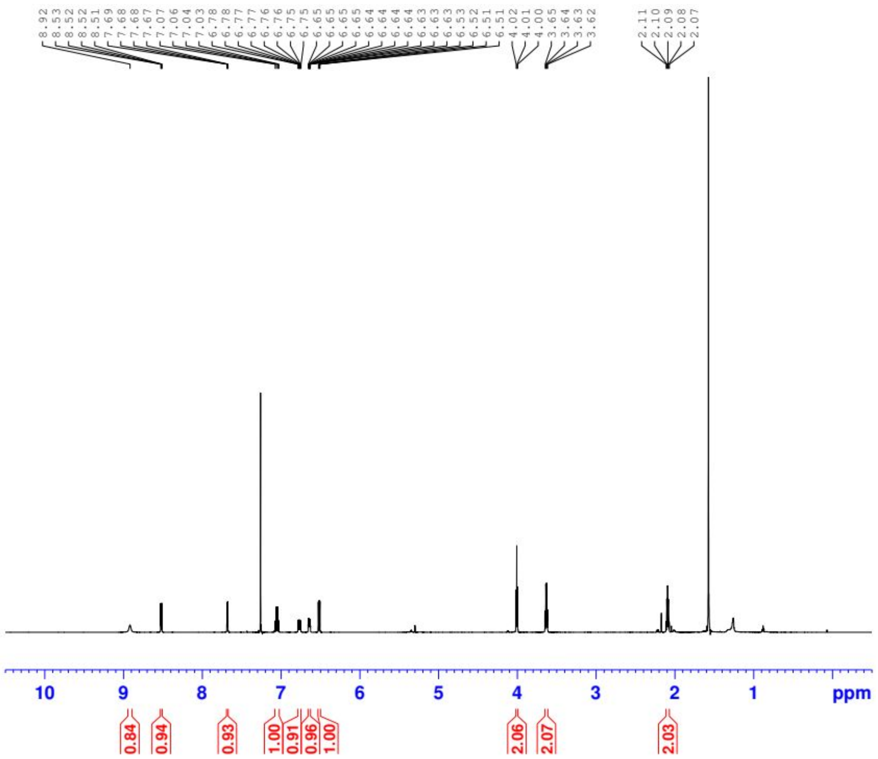

(B) ^{13}C NMR spectra of compound **6p** measured in $CDCl_3$ at 150 MHz

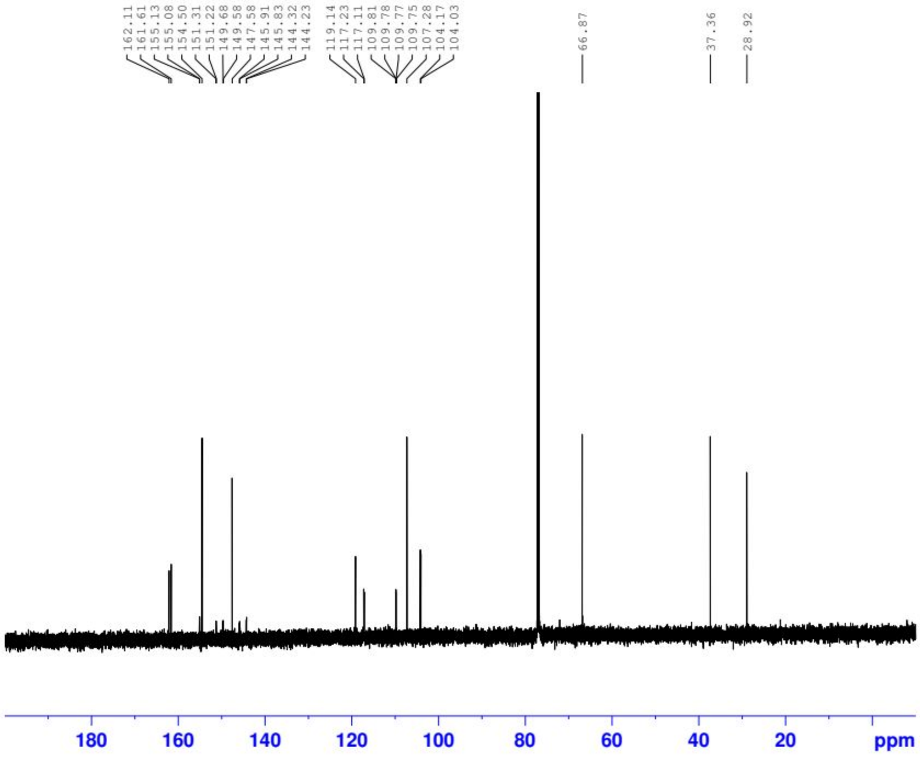

^{1}H NMR (A) and ^{13}C NMR (B) spectra of compound **6p**

^{1}H NMR spectra of compound **6p** measured in $CDCl_3$ at 600 MHzcontrol.

(A) ^{1}H NMR spectra of compound **6q** measured in $CDCl_3$ at 600 MHz

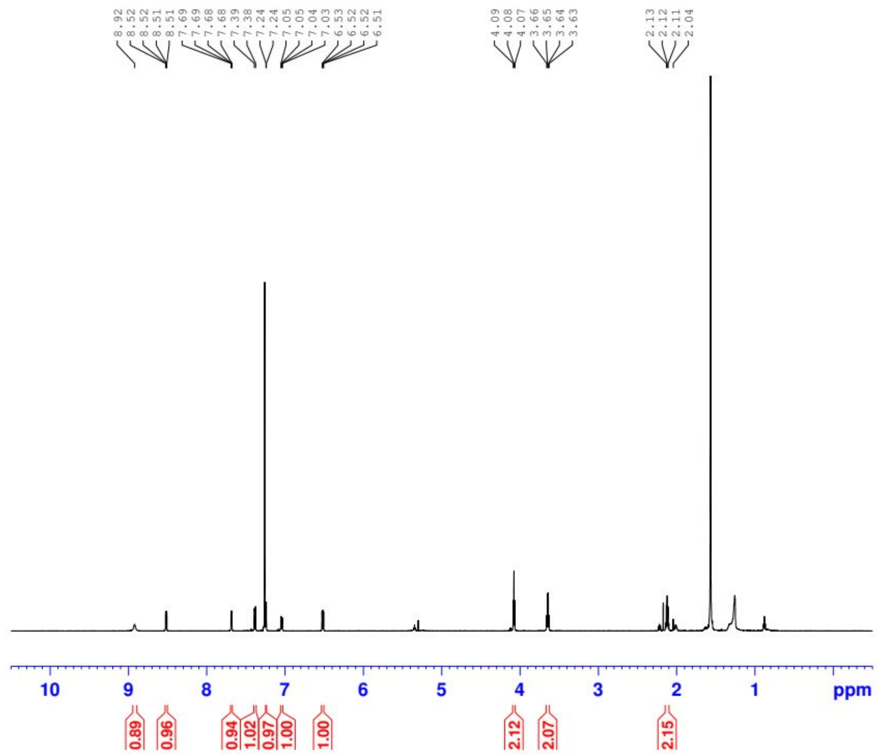

(B) ^{13}C NMR spectra of compound **6q** measured in $CDCl_3$ at 150 MHz

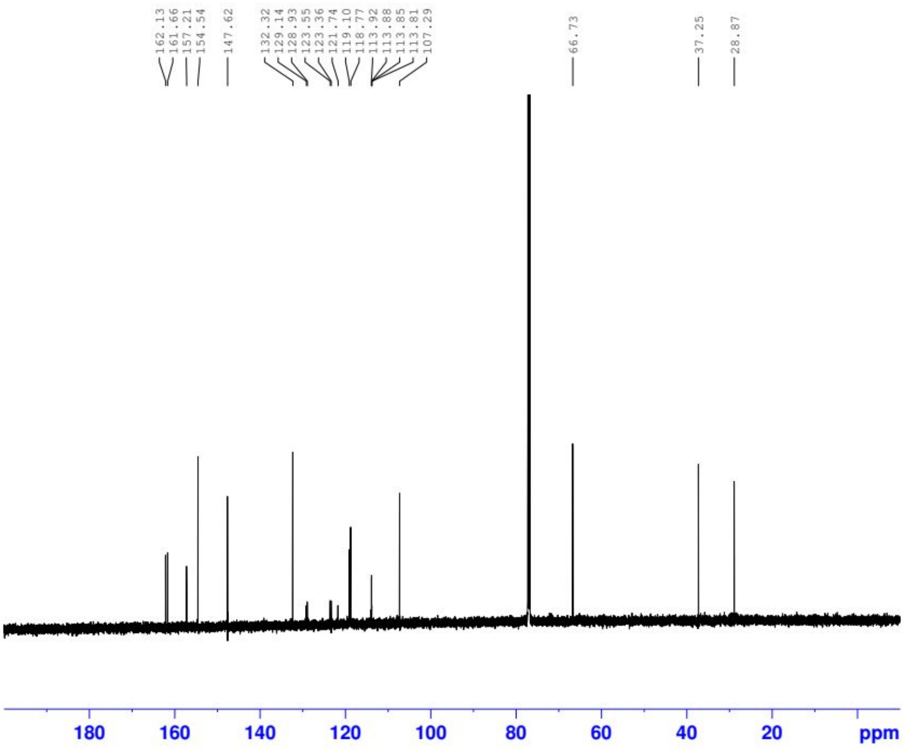

^{1}H NMR (A) and ^{13}C NMR (B) spectra of compound **6q**

^{1}H NMR spectra of compound **6q** measured in $CDCl_3$ at 600 MHzcontrol.

(A) ^{1}H NMR spectra of compound **6r** measured in $CDCl_3$ at 600 MHz

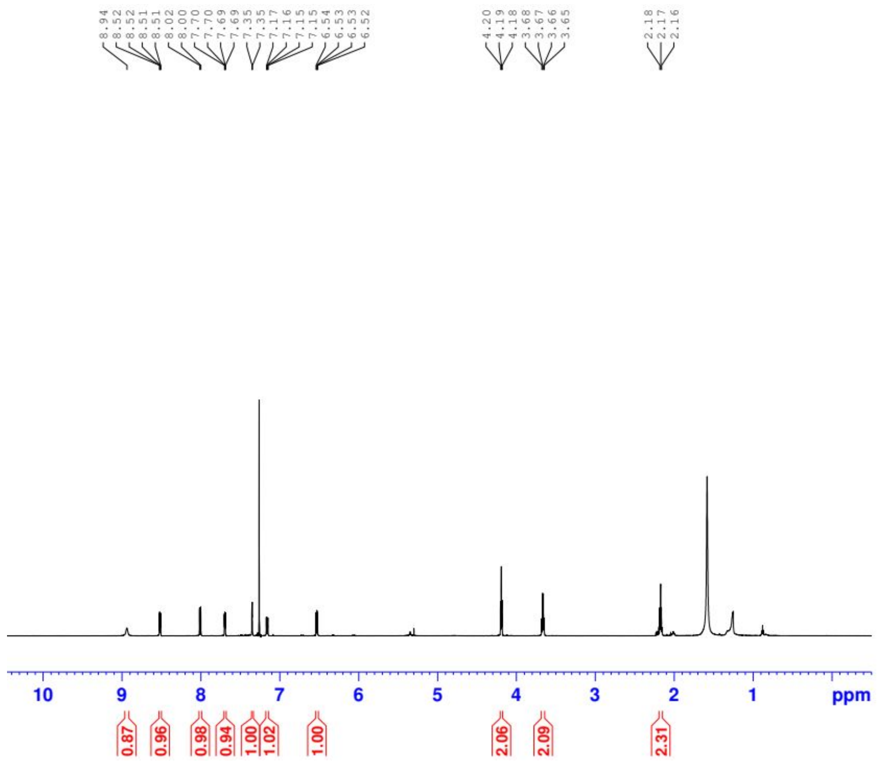

(B) ^{13}C NMR spectra of compound **6r** measured in $CDCl_3$ at 150 MHz

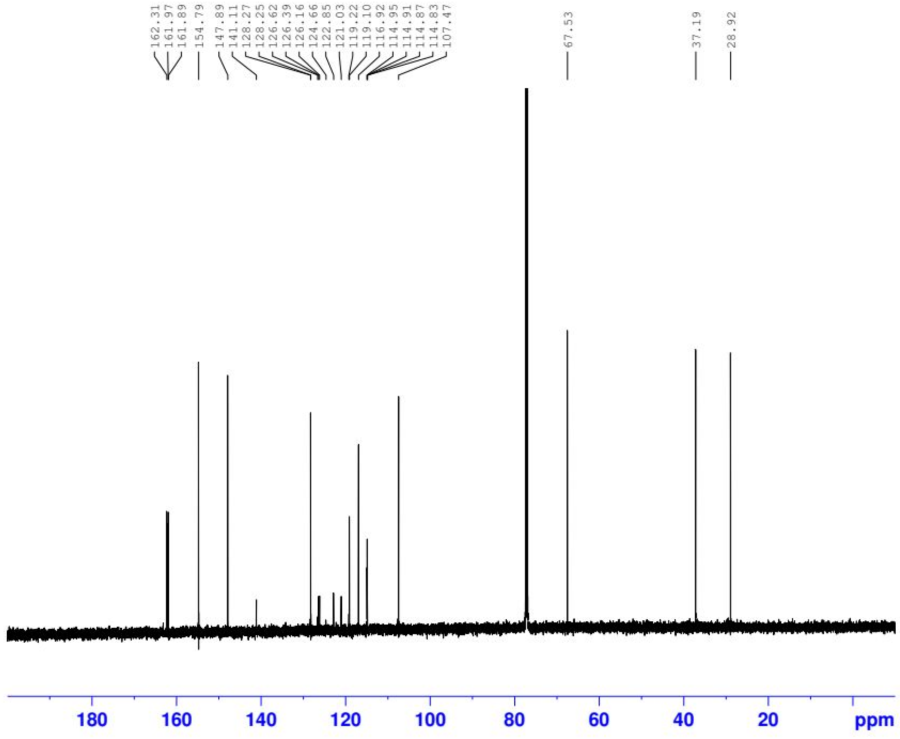

^{1}H NMR (A) and ^{13}C NMR (B) spectra of compound **6r**

^{1}H NMR spectra of compound **6r** measured in $CDCl_3$ at 600 MHzcontrol.

(A) ^{1}H NMR spectra of compound **6s** measured in $CDCl_3$ at 600 MHz

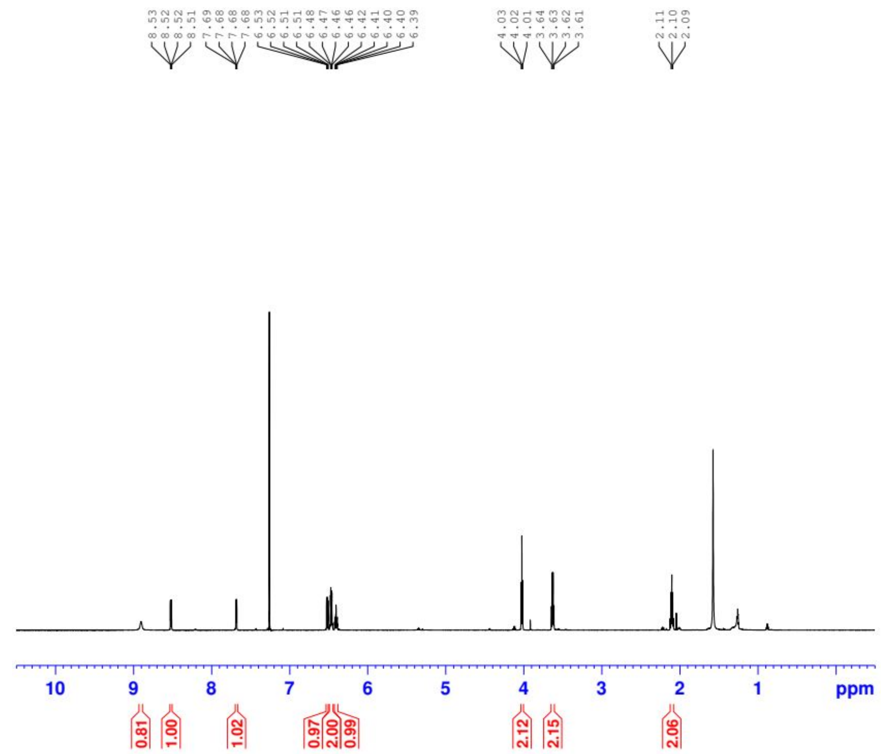

(B) ^{13}C NMR spectra of compound **6s** measured in $CDCl_3$ at 150 MHz

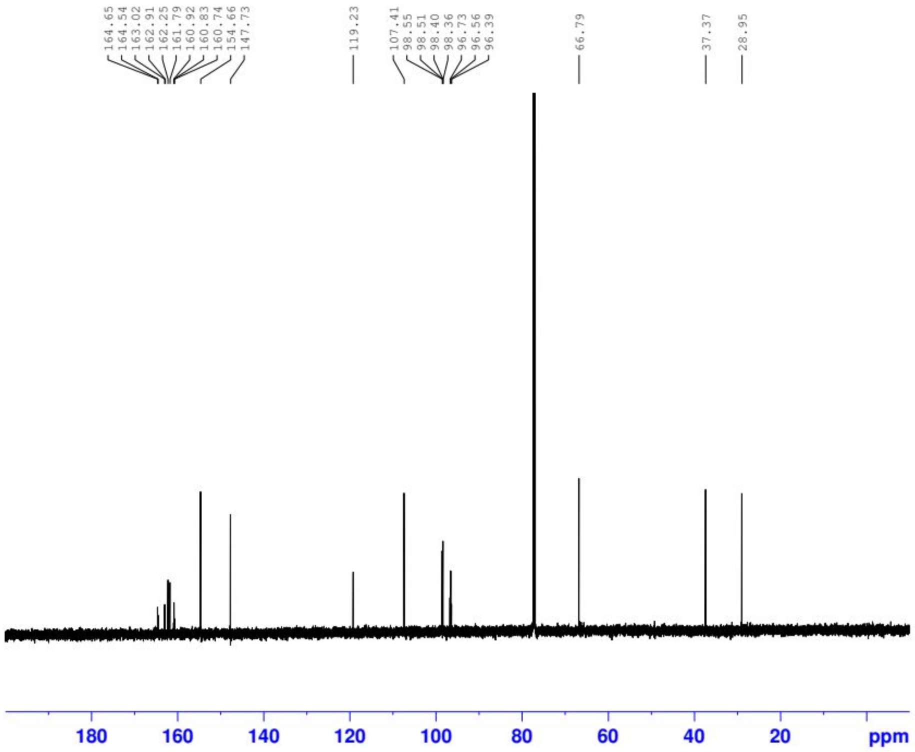

^{1}H NMR (A) and ^{13}C NMR (B) spectra of compound **6s**

^{1}H NMR spectra of compound **6s** measured in $CDCl_3$ at 600 MHzcontrol.

(A) ^{1}H NMR spectra of compound **6t** measured in $CDCl_3$ at 600 MHz

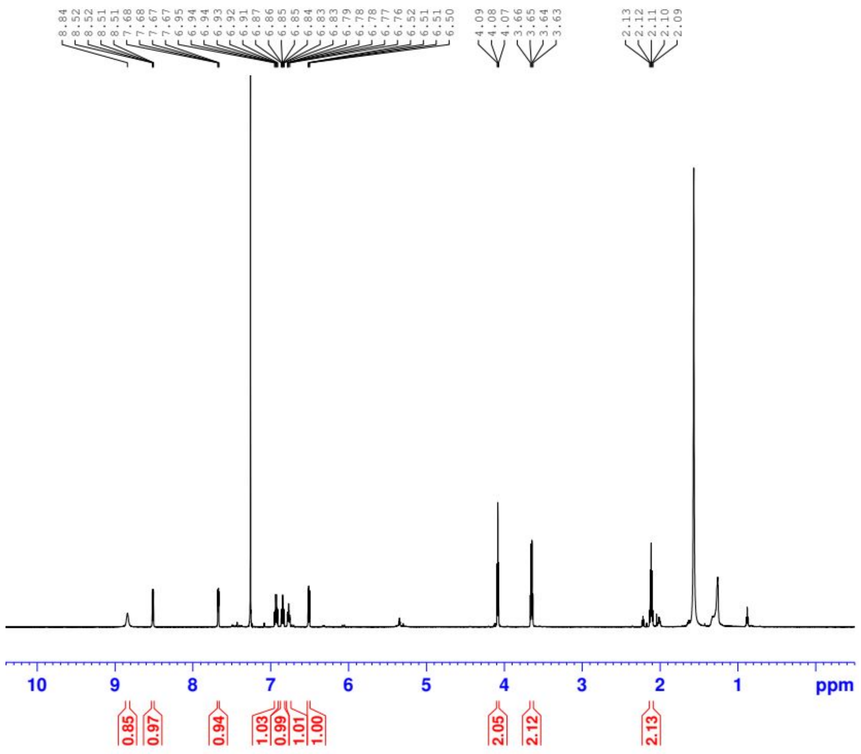

(B) ^{13}C NMR spectra of compound **6t** measured in $CDCl_3$ at 150 MHz

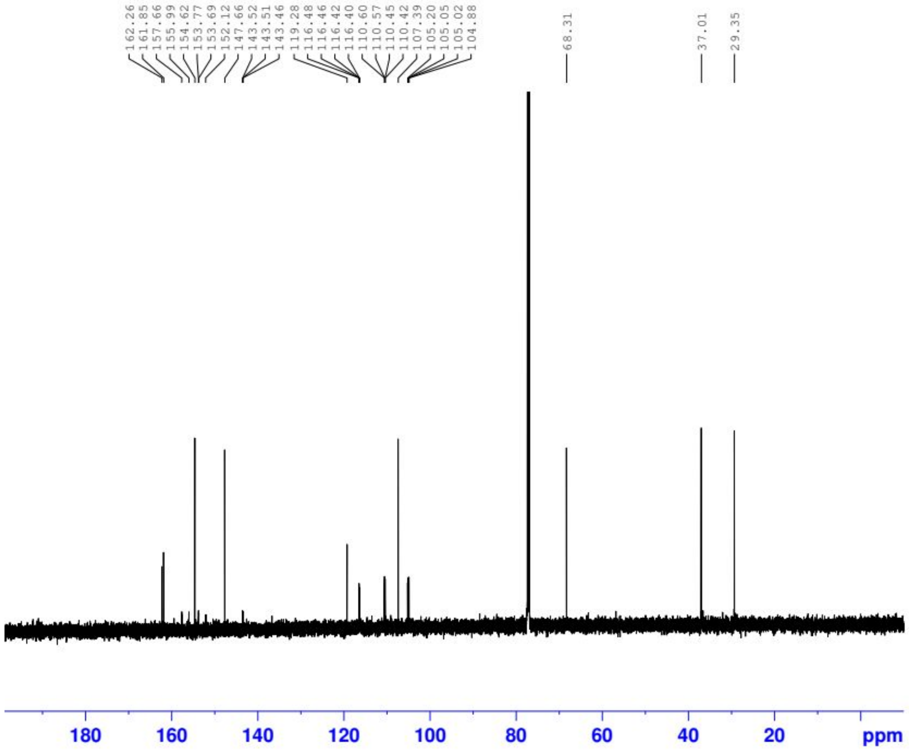

^{1}H NMR (A) and ^{13}C NMR (B) spectra of compound **6t**

^{1}H NMR spectra of compound **6t** measured in $CDCl_3$ at 600 MHzcontrol.

(A) HPLC trace of compound **6a**

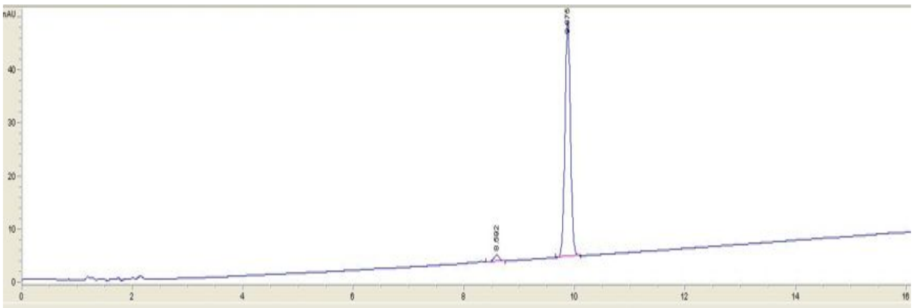

(B) HPLC trace of compound **6b**

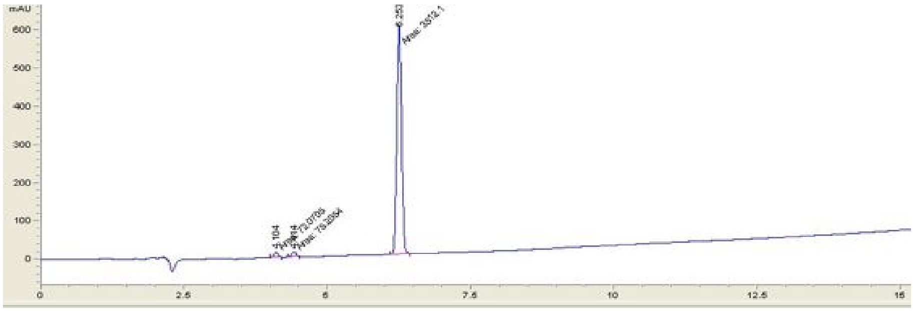

(C) HPLC trace of compound **6c**

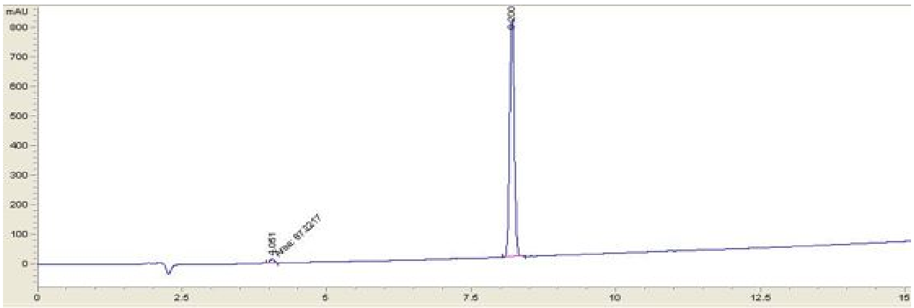

(D) HPLC trace of compound **6d**

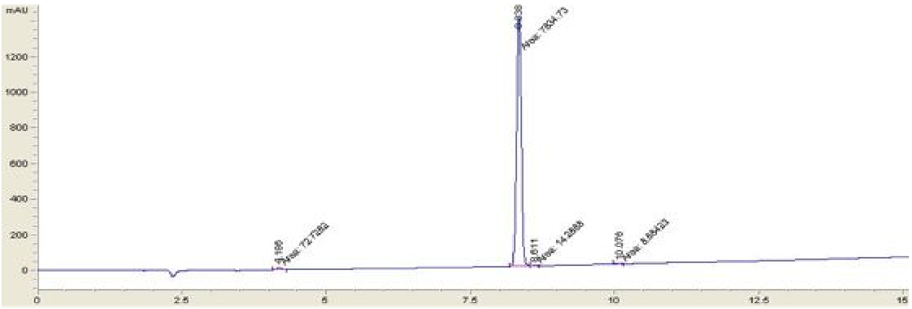

(A) HPLC trace of compound **6e**

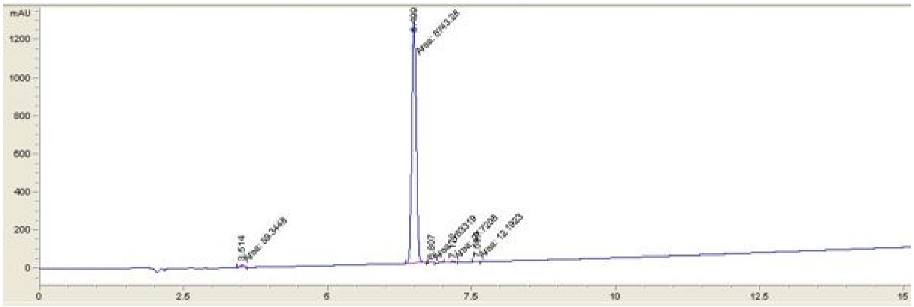

(B) HPLC trace of compound **6f**

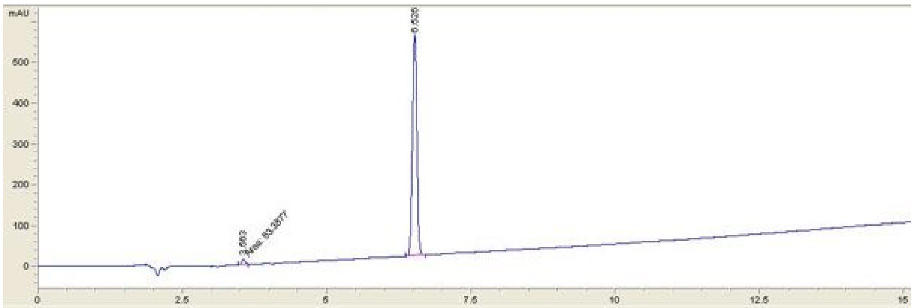

(C) HPLC trace of compound **6g**

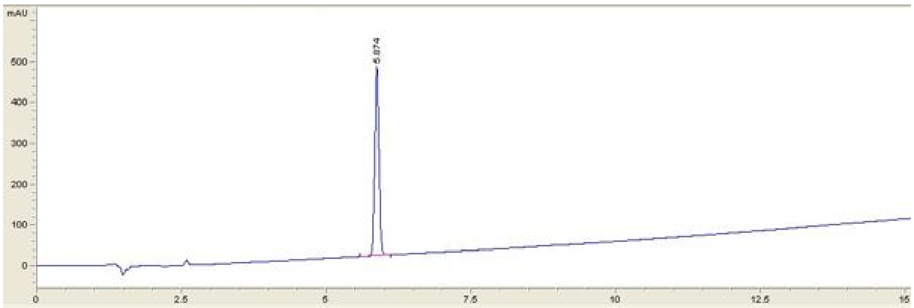

(D) HPLC trace of compound **6h**

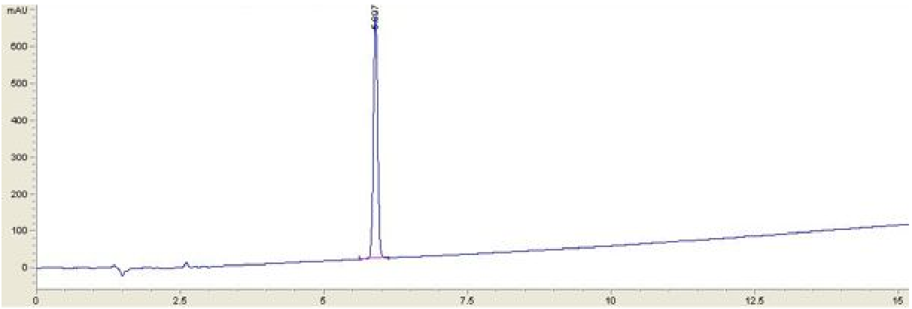

(A) HPLC trace of compound **6i**

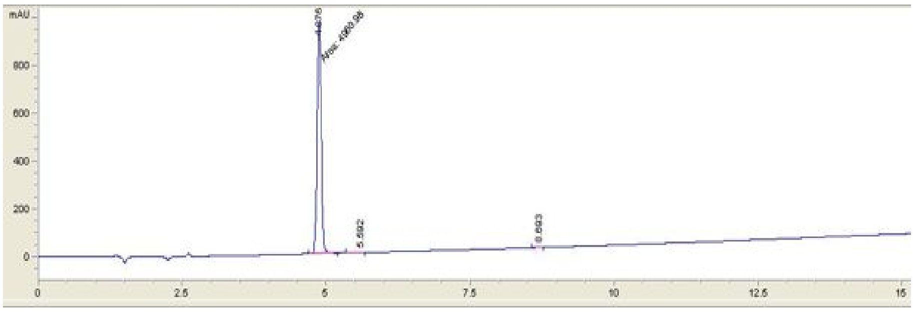

(B) HPLC trace of compound **6j**

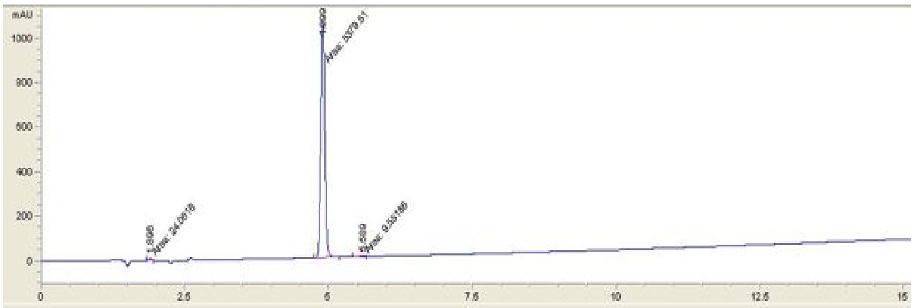

(C) HPLC trace of compound **6k**

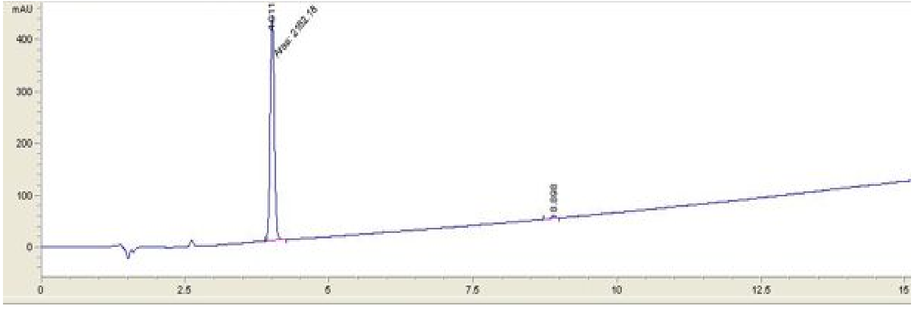

(D) HPLC trace of compound **6l**

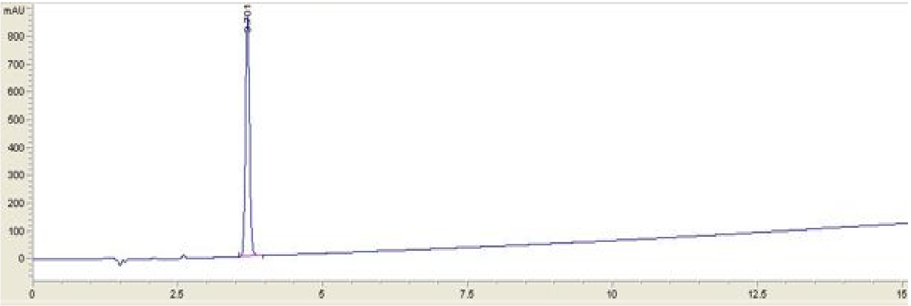

(A) HPLC trace of compound **6m**

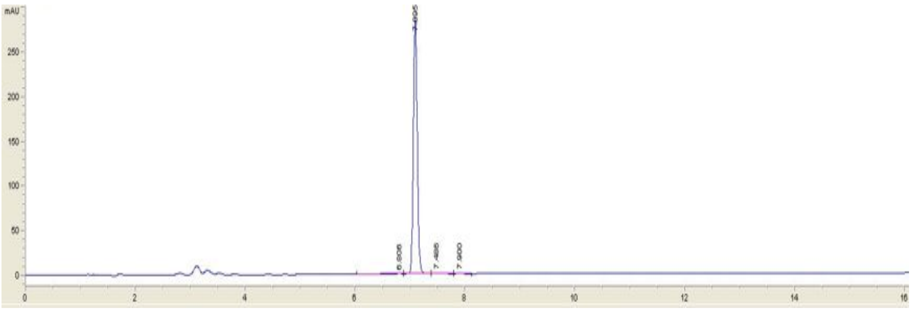

(B) HPLC trace of compound **6n**

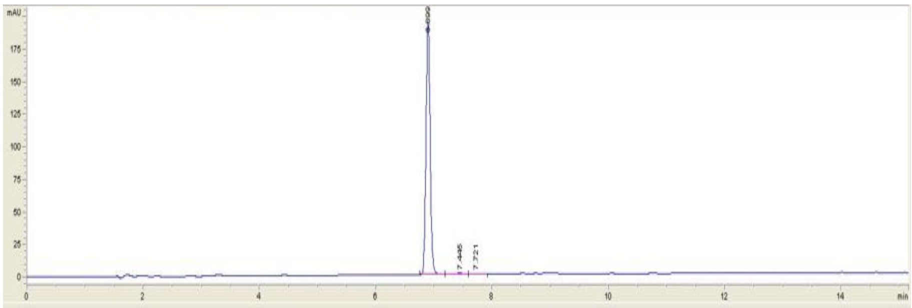

(C) HPLC trace of compound **6o**

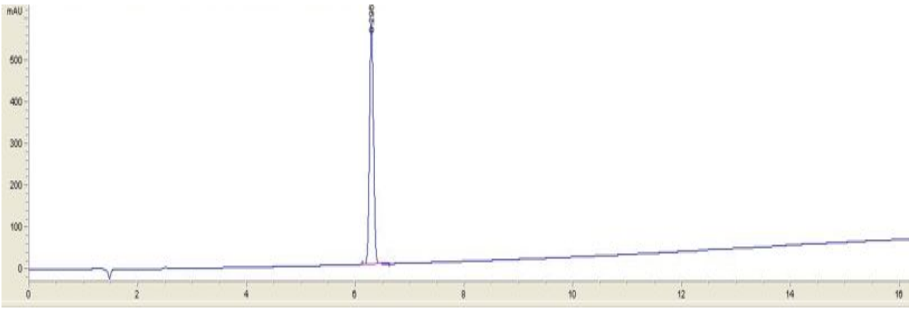

(D) HPLC trace of compound **6p**

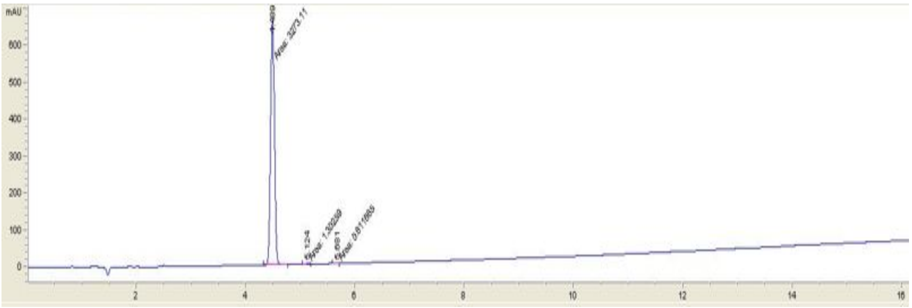

(A) HPLC trace of compound **6q**

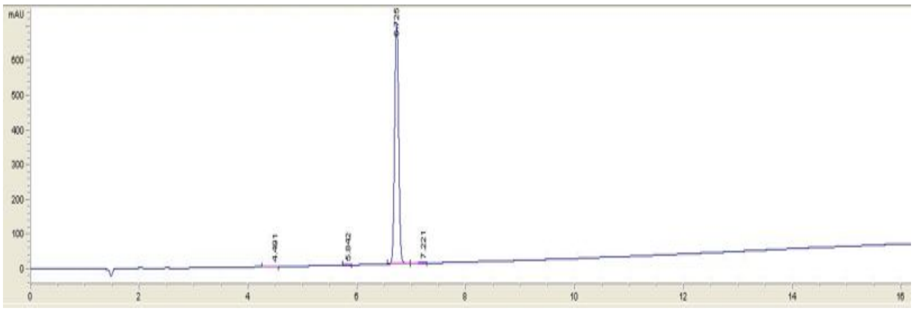

(B) HPLC trace of compound **6r**

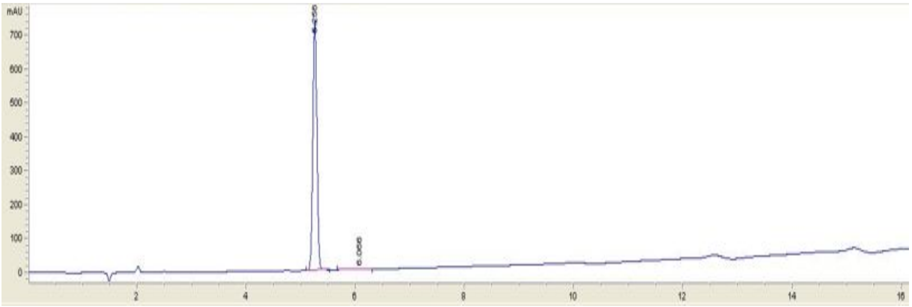

(C) HPLC trace of compound **6s**

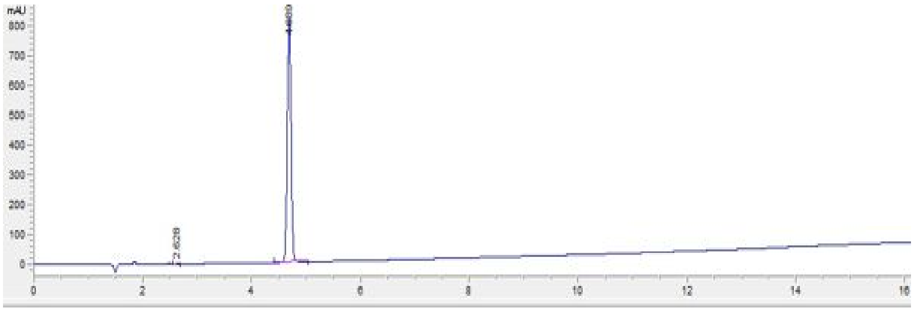

(D) HPLC trace of compound **6t**

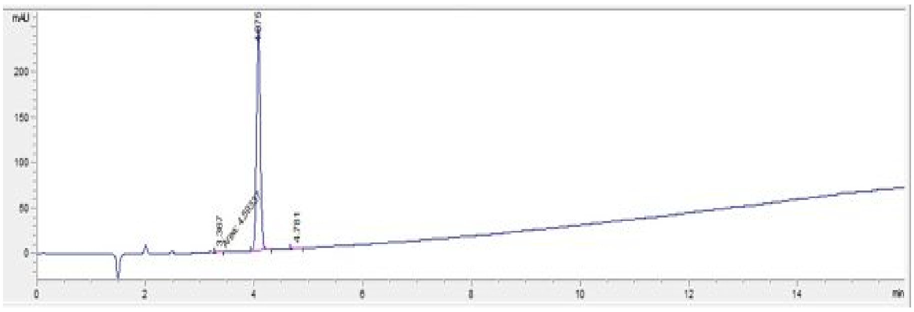

Supplement: Supplementary file 2 [file ao5c04793_si_002.pdf]
